# Diet-induced obesity promotes endothelial cell desensitization to VEGF-A and permanent islet vessel dysfunction in mice

**DOI:** 10.1172/JCI177601

**Published:** 2025-06-05

**Authors:** Yan Xiong, Andrea Dicker, Montse Visa, Erwin Ilegems, Per-Olof Berggren

**Affiliations:** 1 The Rolf Luft Research Center for Diabetes and Endocrinology, Karolinska Institutet, Stockholm, Sweden.; 2 Department of Endocrinology and Metabolism, Center for Diabetes and Metabolism Research, West China Hospital, Sichuan University, Chengdu, China.

**Keywords:** Endocrinology, Metabolism, Vascular biology, Diabetes, Endothelial cells, Molecular pathology

## Abstract

Pancreatic islet microvasculature is essential for optimal islet function and glucose homeostasis. However, islet vessel pathogenesis in obesity and its role in the manifestation of metabolic disorders remain understudied. Here, we depict the time-resolved decline of intra-islet endothelial cell responsiveness to VEGF-A and islet vessel function in a mouse model of diet-induced obesity. Longitudinal imaging of sentinel islets transplanted into mouse eyes revealed substantial vascular remodeling and diminished VEGF-A response in islet endothelial cells after 12 weeks of Western diet (WD) feeding. This led to islet vessel barrier dysfunction and hemodynamic dysregulation, delaying transportation of secreted insulin into the blood. Notably, islet vessels exhibited a metabolic memory of previous WD feeding. Neither VEGF-A sensitivity nor the other vascular alterations was fully restored by control diet refeeding, resulting in modest yet significant impairment in glucose clearance despite normalized insulin sensitivity. Mechanistic analysis implicated hyperactivation of atypical PKC under both WD and recovery conditions, which inhibited VEGFR2 internalization and blunted VEGF-A–triggered signal transduction in endothelial cells. In summary, prolonged WD feeding causes irreversible islet endothelial cell desensitization to VEGF-A and islet vessel dysfunction, directly undermining glucose homeostasis.

## Abstract 2

Long-term western diet feeding causes irreversible islet endothelial cell desensitization to VEGF-A, VEGFR2 internalization impairments and islet vessel dysfunction, which directly undermines glucose homeostasis.

## Graphical abstract

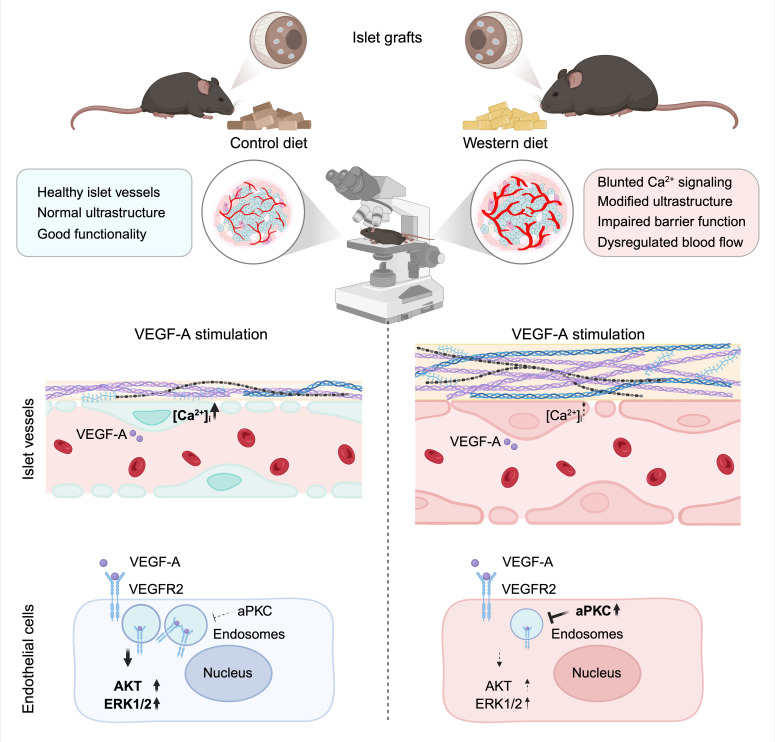

## Introduction

As an integral part of pancreatic islets, the rich microvascular network not only ensures oxygen and nutrition provision for islet survival, but also provides critical developmental cues during pancreatic organogenesis and modulates its endocrine function at later stages (1–3). Islet endothelial cells lining the luminal surface of the vessels are highly fenestrated and serve as gateways for efficient substance exchange between the interstitial fluid of islets and the bloodstream, which is imperative for the islet sensing of plasma nutrient fluctuations as well as for timely hormone outflow (4, 5). Obesity and diabetes are known to elicit morphological remodeling of islet architecture both in humans and rodent models, and suboptimal vessel density is detrimental to islet function and glucose metabolism (6–8). Increased oxidative stress concomitant with these metabolic disorders generally leads to reprogramming and functional modifications in endothelial cells (9, 10); however, due to technical limitations, it remains unclear whether intra-islet vessel functionality is undermined in obesity, which may influence the islet response to glucose.

VEGF-A plays a fundamental and indispensable role in vessel physiology and homeostasis. Upon binding to its principal signaling receptor, VEGFR2, it initiates rapid receptor dimerization and internalization, followed by phosphorylation of key downstream signaling molecules. VEGF-A thereby promotes angiogenesis, increases vascular permeability, and fine-tunes vasomotor activity (11, 12). In pancreatic islets, VEGF-A is constitutively expressed by all types of endocrine cells and signals through the endothelial membrane–bound VEGFR2. This intra-islet paracrine signal regulates islet vascular growth and patterning and coordinates innervation during pancreas development (13–15). Tightly controlled VEGF-A expression is not only essential for the maintenance of the dense and highly permeable islet microvasculature, but it is also crucial for islet adaptation to changes in metabolic demands in terms of insulin secretion and β cell mass (16–20). Moreover, VEGF-A is required for reestablishing blood flow in transplanted islets, which is imperative for graft survival (21). We have previously shown in a ciliopathy mouse model that primary cilia defects lead to VEGF-A/VEGFR2 signaling disruption in endothelial cells and consequently delay revascularization of transplanted islets (22).

Recently, VEGF-A has also been recognized as an underlying factor for metabolic diseases (23, 24). Elevated plasma VEGF-A concentrations were repeatedly reported in obese and diabetic individuals (25–27). Dysregulated VEGF-A expression and signaling activity have also been implicated in aberrant angiogenesis associated with diabetes in a tissue-specific manner (28–31). Upregulated VEGF-A signaling in diabetic retinal vessels leads to excessive angiogenesis, which results in vascular lesions (32), whereas insufficient VEGF-A signaling underlies impaired wound healing (33). In addition, exposure to high glucose concentration diminishes VEGF-A–triggered signal transduction in cultured endothelial cells through the induction of a ligand-independent noncanonical VEGFR2 signaling pathway (34). Here, by integrating state-of-the-art in vivo imaging techniques with multiple vessel function assays, we provide evidence that pancreatic islet endothelial cells develop resistance against VEGF-A under long-term Western diet (WD) feeding. Moreover, we demonstrate that dysregulated VEGFR2 internalization and obstructed downstream signaling underlie irreversible islet vessel function impairments, which compromise the efficiency of insulin transportation and glucose clearance.

## Results

### WD leads to body weight gain, glucose intolerance, and pancreatic islet vascular remodeling.

**Figure 1:**
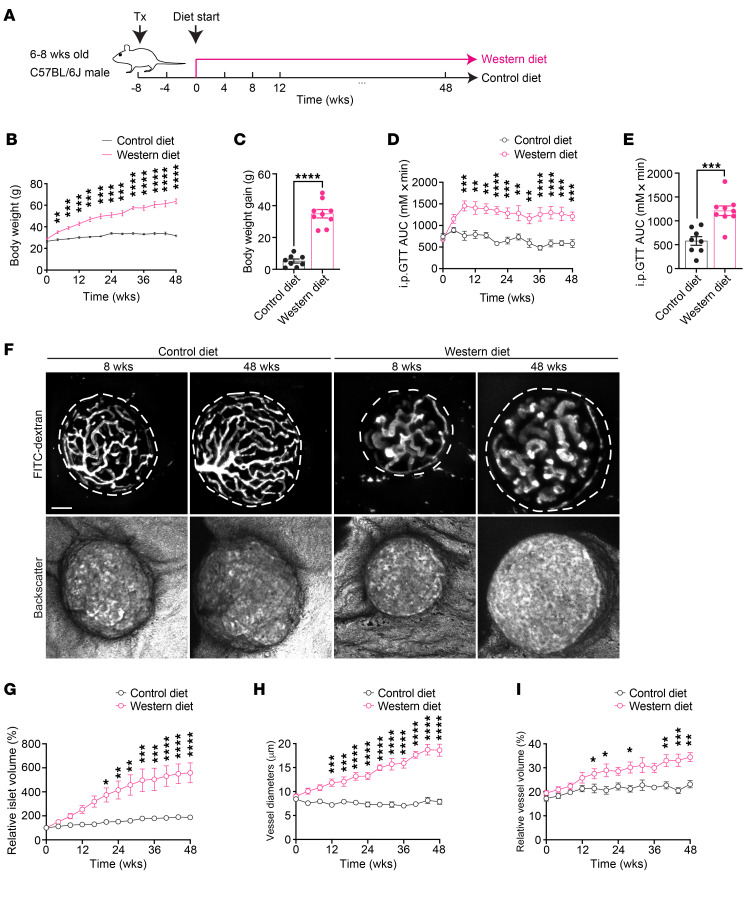
WD leads to body weight gain, glucose intolerance, islet hyperplasia, and islet vascular remodeling. (**A**) Schematic illustration of experimental timeline. Tx, transplantation. (**B** and **C**) Body weight changes during diet intervention (**B**) and average body weight gain in CD- (*n* = 8) and WD-fed (*n* = 9) animals at week 48 (**C**). (**D** and **E**) AUC for intraperitoneal glucose tolerance tests (IPGTTs) during diet intervention (**D**) and at week 48 (**E**) in CD- (*n* = 8) and WD-fed (*n* = 9) animals. (**F**) Morphological changes in islet grafts and vasculature over time in CD- and WD-fed animals. Representative confocal images are presented as maximum intensity projections. Scale bar: 50 µm. (**G**) Relative growth of islet grafts in CD- (*n* = 6) and WD-fed (*n* = 7) animals during diet intervention compared with week 0. (**H** and **I**) Vessel diameters (**H**) and relative vascular volume (**I**) in islet grafts of CD- (*n* = 7) and WD-fed (*n* = 9) animals during diet intervention. Data are shown as individual points (**C** and **E**) or mean ± SEM (the rest). Statistics are based on unpaired, 2-tailed Student’s *t* tests (**C** and **E**) or 2-way ANOVA (the rest). *P < 0.05, **P < 0.01, ***P < 0.001, ****P < 0.0001.

To delineate the progression of pancreatic islet vascular damage in a mouse model undergoing diet-induced obesity, we transplanted syngeneic islets into the anterior chamber of the eye (ACE) of male C57BL/6J mice. Following an 8-week period to allow for full islet engraftment and revascularization, the recipient mice were randomly assigned to either the control diet (CD) or WD group. The diet regimen lasted for 48 weeks, and mice in both groups were examined every 4 weeks (Figure 1A). As expected, WD-fed mice rapidly developed truncal obesity and showed slowing but continuous weight gain throughout the intervention, eventually gaining significantly more weight than the CD-fed mice from week 0 to the endpoint (Figure 1, B and C). Glucose tolerance also deteriorated rapidly in the WD group, although the level of intolerance stabilized after 8 weeks (Figure 1, D and E), verifying the emergence of metabolic disorders. Meanwhile, we monitored the vasculatures of individual islet grafts noninvasively and longitudinally in vivo, since they closely resemble characteristics of in situ pancreatic islet vasculature (35, 36). Through intravenous injection of FITC-labeled dextran, we were able to visualize the reestablished vessel networks in grafted islets, and backscatter signals were obtained simultaneously for the estimation of islet size (Figure 1F) (22). Quantitative analysis showed obvious islet hyperplasia in the WD group (Figure 1G), accompanied by remarkable vascular growth and remodeling. The vessels of islet grafts in WD-fed mice displayed irregularly enlarged diameters starting from 12 weeks after diet intervention (Figure 1H), and they constituted a higher percentage of total islet volume (Figure 1I). By contrast, islet vasculature in the CD group remained relatively stable, with limited growth and structural refinements (Figure 1, F, H, and I).

### Metabolic disorders are associated with increased islet expression and production of VEGF-A.

Vascular remodeling is usually accompanied by functional adaptations. To evaluate islet vascular functionality under WD feeding, we examined islet expression of VEGF-A and VEGFR2, which are indispensable for maintaining the morphological and functional architecture of islet vessels (13, 15). Immunofluorescence staining of VEGF-A and VEGFR2 on pancreatic sections exhibited similar patterns of protein expression between CD- and WD-fed mice after 8 weeks of diet intervention (Supplemental Figure 1, A and B; supplemental material available online with this article; https://doi.org/10.1172/JCI177601DS1). Similar to previous findings, VEGF-A was expressed by all islet endocrine cells, including non–β cells, at a much higher level than the surrounding exocrine cells in both groups (Supplemental Figure 1A). VEGFR2 expression was mostly restricted to the membrane of islet vascular endothelial cells, as labeled by the endothelial cell marker PECAM-1 (Supplemental Figure 1B). Of note, VEGFR2 was also expressed at a markedly higher level in islets than in the exocrine pancreas (Supplemental Figure 1B), consistent with a recent study in human pancreas (37). This indicates that islet endothelial cells may be more susceptible than exocrine endothelial cells to perturbations in VEGF-A/VEGFR2 signaling. Gene expression analysis showed that freshly isolated islets from the WD group expressed more *Vegfa* gene after 12 weeks of diet intervention (Supplemental Figure 1C). As a result, they released more VEGF-A than control islets ex vivo (Supplemental Figure 1D). The kinase insert domain receptor gene (*Kdr,* encoding VEGFR2) was also upregulated in the WD group after 12 weeks of diet intervention compared with the CD group (Supplemental Figure 1E). This may be partly attributed to a relatively augmented number of intra-islet endothelial cells, as reflected by elevated *Pecam1* expression at the same time points (Supplemental Figure 1F), which also supports our observation of increased islet vascular volume in the WD-fed mice (Figure 1I). To determine whether the same pattern holds true in human islets, we conducted gene expression analysis in islets from healthy, obese, prediabetic, and type 2 diabetic (T2D) donors (Supplemental Table 1). Consistent with our mouse data, both *VEGFA* and *KDR* gene expression exhibited a clear upward trend in obesity, while *VEGFA* was significantly upregulated in prediabetes/T2D, showing a strong correlation with hemoglobin A1c (HbA1c) levels (Supplemental Figure 2, A and B). To further validate these findings, we extracted data from a public pancreatic single-cell RNA sequencing dataset, The Human Pancreas Analysis Program, and confirmed that *VEGFA* transcripts in β cells and *KDR* transcripts in islet endothelial cells were indeed increased in T2D donors compared with the healthy controls (Supplemental Figure 2, C–F).

### WD diminishes VEGF-A–triggered islet endothelial cell Ca^2+^ mobilization in vivo.

The binding of VEGF-A ligand to VEGFR2 initiates rapid mobilization of intracellular Ca^2+^, which serves as a good indicator for VEGF-A/VEGFR2 signaling activity (38). Therefore, we generated a mouse line expressing a fluorescent indicator for intracellular Ca^2+^ concentration ([Ca^2+^]_i_) specifically in endothelial cells. By crossing the *Cdh5-Cre* mouse line to the Ai38 line that carries a *loxP* flanked STOP cassette in front of a Ca^2+^ biosensor, GCaMP3 (GC3), we obtained EC-GC3 offspring that were positive for both transgenes and expressed GC3 in all endothelial cells (Supplemental Figure 3A) (39, 40). EC-GC3 animals performed similarly to WT mice in intraperitoneal glucose tolerance tests (Supplemental Figure 3B) and responded to WD with a swift and steady body weight gain similar to C57BL/6J mice (Supplemental Figure 3C). Immunostaining of pancreatic islet sections from EC-GC3 animals verified that GC3 primarily labeled the entire islet vascular network, which was marked by PECAM-1 staining (Supplemental Figure 3D). When EC-GC3 animals were used as recipients for transplantation, islet grafts in the ACE were primarily revascularized by GC3-expressing recipient endothelial cells (Supplemental Figure 3E), making them ideal for monitoring the dynamics of [Ca^2+^]_i_ triggered by VEGF-A in vivo.

**Figure 2:**
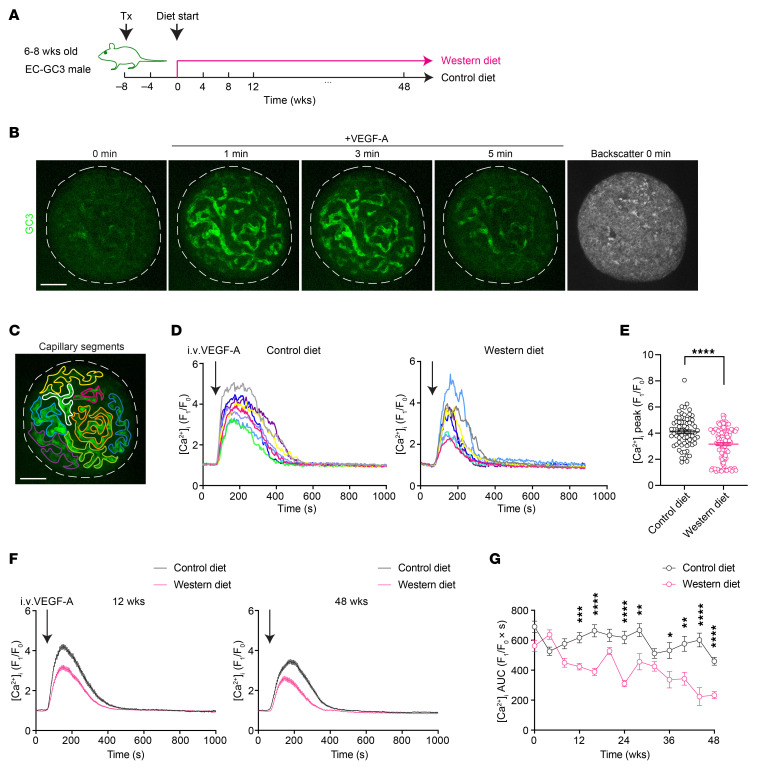
WD diminishes VEGF-A–triggered islet endothelial cell Ca^2+^ mobilization in vivo. (**A**) Schematic illustration of the experimental timeline. (**B**) Representative intra-islet vessel Ca^2+^ response to VEGF-A bolus in the CD-fed animals. (**C**) An example of vessel segment selection within an islet graft. Scale bars: 50 µm. (**D**) Representative Ca^2+^ traces from individual segments within a single islet of CD- (left) and WD-fed (right) animals at week 12. (**E**) Peak Ca^2+^ response in islet vessels of CD- (*n* = 8) and WD-fed (*n* = 12) animals at week 12. (**F**) Averaged Ca^2+^ traces in islet vessels of CD- and WD-fed animals at week 12 (left; CD: *n* = 8; WD: *n* = 12) and week 48 (right; CD: *n* = 6; WD: *n* = 6). (**G**) Average AUC for Ca^2+^ traces in CD- (*n* = 6–12) and WD-fed (*n* = 6–13) animals throughout the diet regimen. Data are shown as individual points (**E**) or mean ± SEM (the rest). Statistics are based on unpaired, 2-tailed Student’s *t* tests (**E**) or 2-way ANOVA (**G**). *P < 0.05, **P < 0.01, ***P < 0.001, ****P < 0.0001.

We followed the previous timeline of investigation and subjected EC-GC3 mice to the same diet regimen 8 weeks after transplantation (Figure 2A). VEGF-A–induced [Ca^2+^]_i_ dynamics in islet endothelial cells was then examined every 4 weeks to obtain a temporal overview of intra-islet VEGF-A/VEGFR2 signaling profiles in the CD and WD groups and capture the emergence of any signaling disturbance induced by the diet. Baseline GC3 fluorescence in islet endothelial cells was generally low prior to stimulation, which remained stable and of comparable intensity in the CD and WD groups over time (Supplemental Figure 3, F and G). When VEGF-A was administered intravenously, we observed an instant increase in GC3 fluorescence, which peaked at about 1.5 minutes after injection and remained elevated for another 4 minutes before returning to baseline level (Figure 2B). To quantitatively analyze this response, all visible intra-islet vascular segments were selected, and each [Ca^2+^]_i_ trace was plotted individually as fold increase in GC3 fluorescence over baseline intensity (Figure 2C). After 12 weeks of diet intervention, vessel segments within the same islet in the CD group exhibited relatively uniform responses to VEGF-A, in the form of a distinct single raise in GC3 intensity (Figure 2D and Supplemental Video 1). However, although no signaling delay was detected, vessel segments in the WD group showed diverse responses to VEGF-A (Figure 2D and Supplemental Video 2). While a few vessel segments in this group showed similar traces to the control segments, others had smaller peaks and/or shorter duration of response. A closer inspection of the peak Ca^2+^ responses revealed a distinct subgroup of islet vessel segments with reduced sensitivity to VEGF-A stimulation in WD-fed mice after 12 weeks of diet consumption (Figure 2E). When we averaged all [Ca^2+^]_i_ traces from individual islet vessel segments during the course of diet regimen, VEGF-A–induced Ca^2+^ mobilization in the WD group became visibly less than that in the CD group at 12 weeks, and the difference grew more pronounced by the end time point (Figure 2F). Using AUC as a measure of total Ca^2+^ mobilization, we detected a modest aging-related decline in the CD-fed mice towards 48 weeks (Figure 2G). By contrast, the WD group displayed a sharper downward trend throughout the diet regimen, although there were signs of adaptation until week 32, as evidenced by the small fluctuations along the curve (Figure 2G).

### WD undermines VEGF-A regulation of islet hemodynamics.

**Figure 3:**
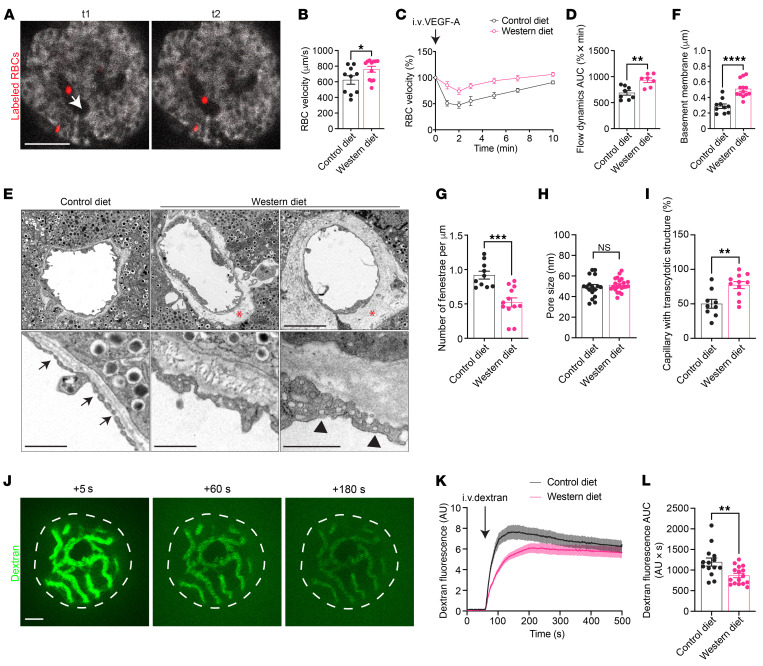
WD undermines VEGF-A regulation of islet hemodynamics and islet vascular barrier function. (**A**) Movement tracking of labeled individual RBCs (red) in the bloodstream in two consecutive time-series images (time interval between t1 and t2 is 18 ms). Arrow indicates the direction of blood flow. Scale bar: 50 mm. (**B**) Baseline RBC velocity in CD- (*n* = 10) and WD-fed (*n* = 12) animals at week 12. (**C**) Average RBC velocity dynamics upon VEGF-A injection and (**D**) AUC for RBC velocity excursions in CD- (*n* = 8) and WD-fed (*n* = 7) animals at week 12. (**E**) Electron micrographs of islet grafts dissected from the ACE of CD- and WD-fed animals at week 12. Asterisks indicate basement membrane, arrows indicate fenestrae, and arrowheads indicate transcytotic structures. Scale bars: 5 mm (upper), 1 mm (lower). (**F–I**) Basement membrane thickness (**F**), number of fenestrae (**G**), pore sizes of fenestrae (**H**), and percentage of capillaries with transcytotic structure (**I**) in islet vessels of CD- (*n* = 9) and WD-fed (*n* = 11) animals at week 12. (**J**) Representative images showing distribution of FITC-dextran inside and around islet grafts 5 (left), 60 (middle), and 180 s (right) after intravenous injection. Scale bar: 50 µm. (**K** and **L**) Average dextran fluorescence intensity dynamics outside islet grafts upon injection in CD- (*n* = 7) and WD-fed (*n* = 8) animals at week 12 (**K**) and AUC of dextran leakage within the first 3 minutes after injection (**L**). Data are shown as mean ± SEM (**C** and **K**) or individual points (the rest). Statistics are based on unpaired, 2-tailed Student’s *t* tests (the rest). *P < 0.05, **P < 0.01, ***P < 0.001, ****P < 0.0001, and NS (*P* > 0.05).

Islet perfusion is coupled to the overall metabolic demand and is thus pivotal for optimal glycemic control (41–43). VEGF-A–triggered intracellular Ca^2+^ mobilization elicits endothelium-dependent vasodilation through rapid production of nitric oxide (44–46), which is counteracted by endothelin-1 release and consequent vasoconstriction (47, 48). In this respect, we investigated if the vasoactive effects of VEGF-A were also undermined by WD feeding. We used RBC flux as a direct measure for the speed of islet blood flow and examined the real-time fluctuations caused by VEGF-A application in vivo. A small number of RBCs in each mouse were prelabeled by a lipophilic fluorescent dye (49), and the movement of individual RBCs was traced in fast time-series scans for estimating the velocity of local blood flow (Figure 3A and Supplemental Video 3). Baseline blood flow velocity was moderately higher in WD-fed mice after 12 weeks of feeding (Figure 3B), which may be attributed to elevated systemic blood pressure (50). An instant decrease in islet RBC flow velocity was observed in CD-fed mice following intravenous VEGF-A administration, which reached its lowest level 2 minutes later and gradually recovered to baseline levels within 10 minutes (Figure 3C). Notably, the sharp dip in flow velocity was markedly attenuated in WD-fed mice after 12 weeks of feeding, and VEGF-A–induced change in islet blood flow was minimal in comparison with the control group (Figure 3, C and D).

### WD modifies islet vessel ultrastructure and alters vessel permeability.

Apart from the dynamic islet blood flow, another vascular element that affects the efficiency of islet action is the barrier function, which relies on the unique ultrastructure and permeability of islet vessels. The deposition of the basement membrane that separates islet endocrine and vascular cells is modulated by VEGF-A (18, 51), and the high degree of fenestration is primarily maintained by active VEGF-A signaling (52–54). To investigate these properties, we dissected islet grafts from mice fed with CD and WD at specific time points and analyzed islet vessel ultrastructure using transmission electron microscopy. Images obtained from the CD group showed a thin layer of basement membrane between endothelial cells and granulated endocrine cells. On the contrary, this layer was significantly and often unevenly thickened in the WD group at 12 weeks (Figure 3, E and F), which not only increased the travel distance for molecules between islet endocrine cells and the bloodstream, but also added to the stiffness of islet vascular walls, undermining their elasticity (55). The endothelial cell lining of islet vessels was densely fenestrated in control islet grafts, whereas the number of fenestrae was notably reduced in the WD group (Figure 3, E and G), although the pore sizes in both groups remained comparable (Figure 3H; approximately 50 nm on average). In addition, we observed an increased prevalence of caveolae-like transcytotic structures in islet vessels from the WD group compared with the CD group (Figure 3, E and I), likely serving as a compensatory mechanism for the loss of fenestration. To corroborate these findings in situ, we conducted the same analysis on pancreatic samples from CD- and WD-fed mice after 12 weeks of diet consumption and detected parallel ultrastructure remodeling in pancreatic islet vessels (Supplemental Figure 4).

The fenestrated islet endothelium is highly permeable and allows quick equilibration between islet interstitial fluid and the bloodstream, facilitating islet glucose sensing and insulin disposal. Considering the ultrastructural alterations in WD-fed mice, we investigated if the islet vessel permeability and barrier function in these mice were compromised. Adopting a similar approach as previously described (22), we gave mice in the CD and WD groups a single intravenous injection of FITC-labeled dextran with a molecular weight of 3–5 kDa and measured its diffusion from islet grafts into the aqueous humor over time (Figure 3J). Upon dextran injection, there was an immediate increase of FITC fluorescence outside the islet grafts, followed by a plateau that was established 1–2 minutes later, as a result of the active drainage of aqueous humor, renal excretion, and reequilibration of dextran (56, 57). The initial dextran leakage rate was markedly reduced in islet grafts of the WD-fed mice, and they reached the fluorescence plateau later than the CD-fed mice (Figure 3K). Consequently, the amount of dextran leakage during the imaging period, as measured by AUC, was lower in the WD group in comparison with the CD group (Figure 3L), suggesting that islet vessels of WD-fed mice were less permeable to molecules of this size.

### Dysfunctional islet vessels hinder insulin transportation in vivo despite intact secretory capacity of islet β cells.

**Figure 4:**
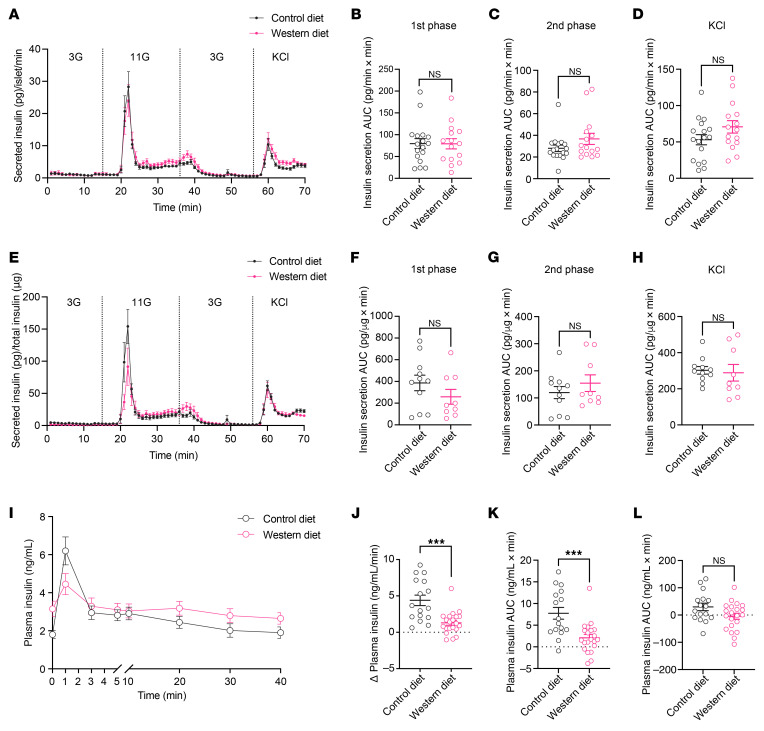
Dysfunctional islet vessels hinder insulin outflow in vivo. (**A**–**D**) Dynamic insulin secretion from freshly isolated islets in a perifusion system, normalized to islet number (**A**), and AUC for glucose-induced first-phase (**B**), second-phase (**C**) and KCl-induced (**D**) insulin secretion. Islets were obtained from CD- (*n* = 4–7) and WD-fed animals (*n* = 3–6) at week 12. (**E**–**H**) Same analysis for dynamic insulin secretion as above but normalized to total insulin content. (**I**) Plasma insulin excursions during IVGTT in CD- (*n* = 16) and WD-fed (*n* = 21) animals at week 12. (**J**) Increase in plasma insulin concentrations during the first minute of IVGTT in CD- (*n* = 16) and WD-fed (*n* = 21) animals at week 12. (**K** and **L**) AUCs for insulin excursions during the first 3 minutes (**K**) and by the end of the tests (**L**) in CD- (*n* = 16) and WD-fed (*n* = 21) animals at week 12. Data are shown as mean ± SEM (**A**, **E**, and **I**) or individual points (the rest). Statistics are based on unpaired, 2-tailed Student’s *t* tests. ***P < 0.001 and NS (*P* > 0.05).

To determine the actual impact of islet vascular barrier dysfunction on glucose homeostasis, we compared insulin secretion and distribution kinetics of islets from CD- and WD-fed mice. Freshly isolated islets from mice fed with CD or WD for 12 weeks were cultured for 2 hours before being packed in columns that were connected to an ex vivo perifusion system. The short cultivation period minimizes changes in β cell gene and protein expression so that the glucose sensing and insulin secretory machinery resemble the in situ condition as closely as possible. Packed islets were subsequently perifused with different concentrations of glucose and KCl, and the perfusate was continuously collected for insulin measurement. In this case, solutions containing secretagogues reached β cells via passive diffusion, and secreted insulin was released from islets directly into the perfusate, both without the need for islet vessels. Dynamic insulin secretion per islet in response to 11 mM glucose and KCl was identical in islets from CD- and WD-fed mice (Figure 4, A–D). Considering the potential impact of obesogenic diets on islet β cell ratio and insulin content, we also normalized the amount of secreted insulin against total insulin content. In this case, we observed a trend for slightly decreased glucose-induced, first-phase insulin secretion in islets from WD-fed mice, although not statistically significant (Figure 4, E–H). Overall, after 12 weeks of WD feeding, β cell response to glucose per se remained intact when they were separated from the unhealthy body environment caused by diet-induced obesity. We then examined insulin release kinetics in vivo during an intravenous glucose tolerance test (IVGTT), where well-controlled glucose delivery to β cells and insulin outflow into the bloodstream rely on the native islet vasculature. As predicted, WD-fed animals displayed an upshifted blood glucose excursion after 4 weeks of diet consumption (Supplemental Figure 5A), and they remained significantly intolerant as the diet intervention continued (Supplemental Figure 5B). During an intravenous glucose challenge at week 12, plasma insulin levels peaked shortly after glucose injection and declined almost to the baseline level by 3 minutes in both groups (Figure 4I). Nonetheless, the initial rise in plasma insulin levels induced by glucose injection was sharp in CD-fed mice but blunted in WD-fed mice, and the average plasma insulin concentration at 1 minute was markedly lower in WD-fed mice despite higher baseline concentration (Figure 4, I and J). Moreover, the amount of secreted insulin that crossed the endothelial barrier into the bloodstream during the first 3 minutes of glucose challenge (measured as incremental AUC) was also smaller in WD-fed mice (Figure 4K). The total amount of insulin that reached the systemic circulation during the entire test period, however, was indistinguishable between the two groups, as evidenced by similar AUCs (Figure 4L). Considering the factors that may influence insulin response in obesity, such as efficiency of insulin clearance by hepatocytes and functionality of peripheral vessels, we further compared the circulating c-peptide levels during intravenous glucose tests on CD- and WD-fed mice. Consistent with our insulin measurements, the acute c-peptide release upon glucose challenge was diminished in the WD group, although similar amounts of c-peptide were released into the blood in both groups by the end of the test (Supplement Figure 5, C–F).

### Islet pathogenesis elicited by WD is only partly reversible by diet normalization.

**Figure 5:**
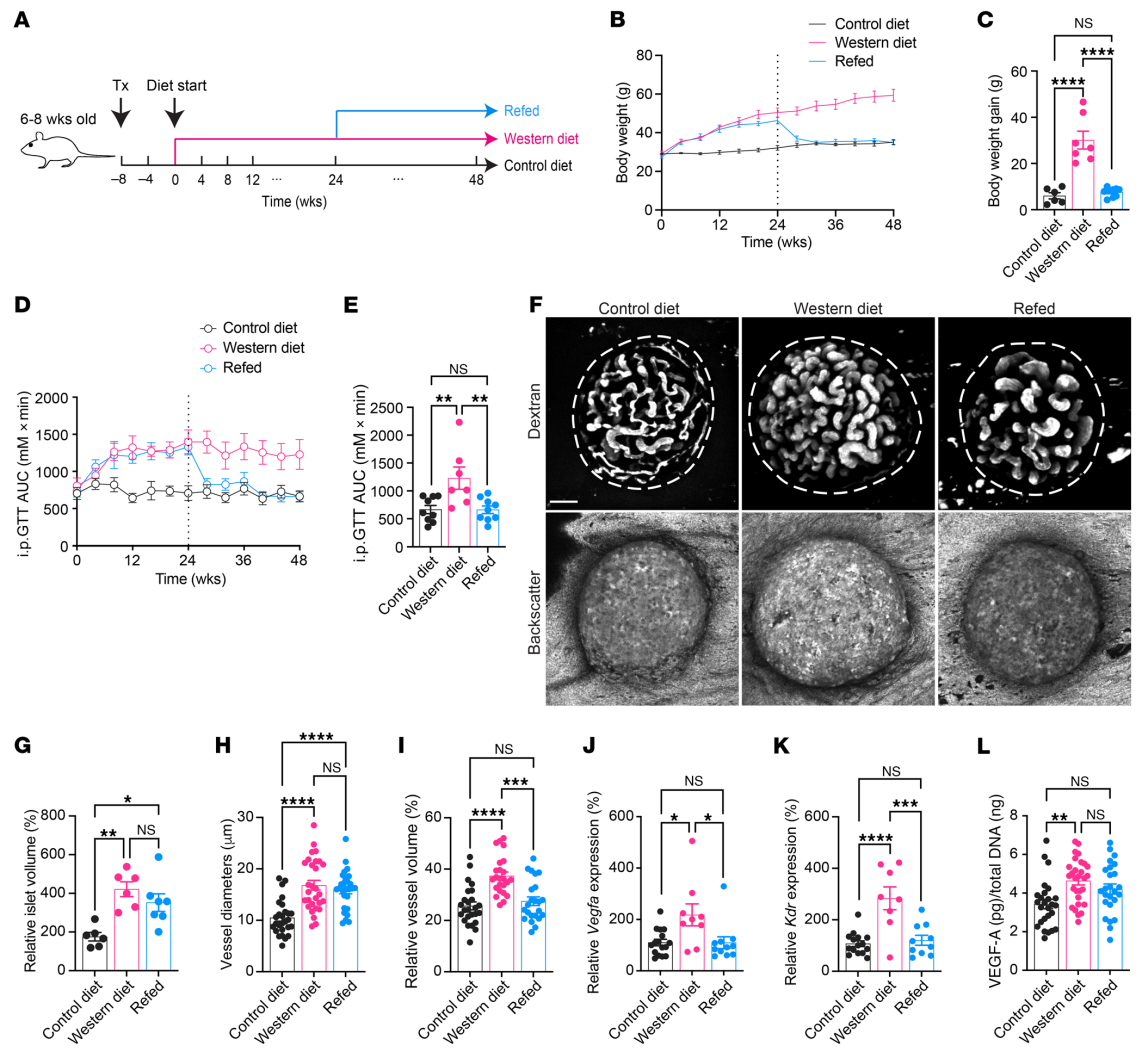
Diet reversal partly restores islet pathogenesis. (**A**) Schematic illustration of the experimental timeline. (**B** and **C**) Body weight changes during diet intervention (**B**) and the average body weight gain in animals from CD (*n* = 6), WD (*n* = 7), and refed (*n* = 9) groups at week 48 (**C**). (**D** and **E**) AUC for IPGTT during diet intervention (**D**) and at week 48 (**E**) in animals from CD (*n* = 9), WD (*n* = 7), and refed (*n* = 9) groups. (**F**) Morphology of islet grafts and their vasculature in CD, WD, and refed groups of animals at week 48. Representative confocal images are presented as maximum intensity projections. Scale bar: 50 µm. (**G**) Relative growth of islet grafts in animals from CD (*n* = 6), WD (*n* = 6), and refed (*n* = 7) groups at week 48 compared with week 0. (**H** and **I**) Vessel diameters (**H**) and relative vascular volume (**I**) in islet grafts of animals from CD (*n* = 7), WD (*n* = 6), and refed (*n* = 9) groups at week 48. (**J** and **K**) Gene expression levels of *Vegfa* (**J**) and *Kdr* (**K**) in freshly isolated islets from CD (*n* = 15–16), WD (*n* = 8–9), and refed (*n* = 10–11) groups of animals at week 48. (**L**) VEGF-A production in cultured islets from CD (*n* = 6), WD (*n* = 6), and refed (*n* = 8) groups of animals at week 48. Data are shown as mean ± SEM (**B** and **D**) or individual points (the rest). Statistics are based on 1-way ANOVA. *P < 0.05, **P < 0.01, ***P < 0.001, ****P < 0.0001, and NS (*P* > 0.05).

Islet β cells exhibit a remarkable level of plasticity in their mass and functionality to meet the demands for insulin under various conditions (58, 59). To investigate the potential reversibility of islet endothelial cell damage by WD, we introduced another diet group that was initially fed with WD for 24 weeks and then switched to CD for the remaining 24 weeks (Figure 5A, Refed). Mice in the refed group quickly lost the excess body weight gained while they were on WD and became as lean as control mice after 4 weeks (Figure 5, B and C). Consequently, their intraperitoneal glucose tolerance was promptly improved (Figure 5, D and E). Nevertheless, islet graft expansion throughout the entire time course remained more pronounced in refed mice than CD-fed mice, although the difference was not as striking as that between the CD and WD groups (Figure 5, F and G). Furthermore, after 24 weeks of refeeding, the vasculature of islet grafts still resembled that of WD-fed mice (Figure 5F). The average vessel diameter in the refed group approximated that of the WD group and remained notably larger than the CD group (Figure 5H). Intriguingly, the relative vascular volume of the refed group became indiscernible from that of the CD group and smaller than the WD group (Figure 5I), which may be attributed to decelerated growth of islet vessels in refed mice. Gene expression analysis of islets derived from all the three groups showed that *Vegfa* and *Kdr* expression was normalized by removal of the dietary stressors (Figure 5, J and K), and islet VEGF-A production was also restored to that of the control level (Figure 5L).

### VEGF-A desensitization and ultrastructure-related barrier function impairment persist in islet endothelial cells after diet reversal.

**Figure 6:**
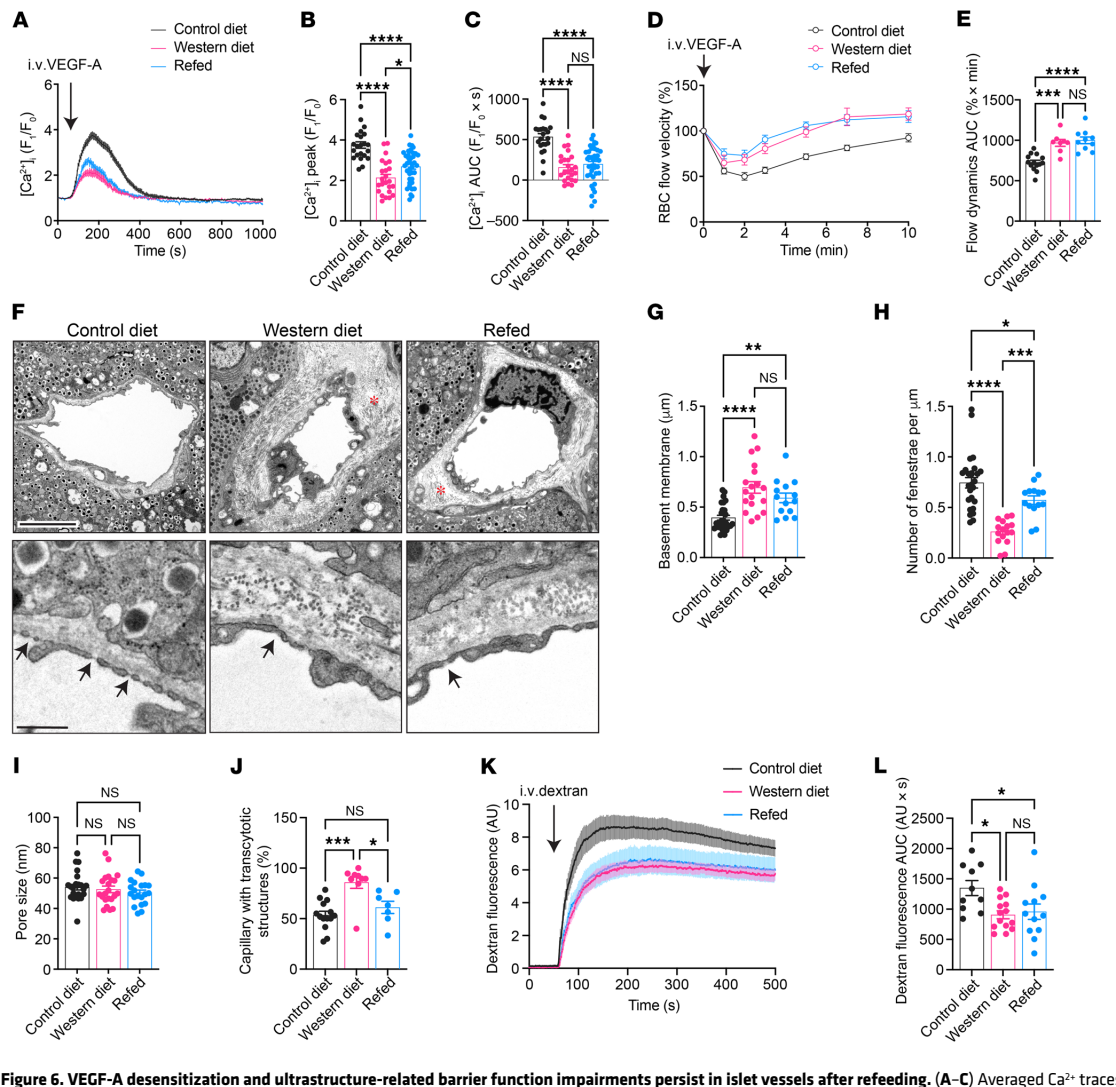
VEGF-A desensitization and ultrastructure-related barrier function impairments persist in islet vessels after refeeding. (**A**–**C**) Averaged Ca^2+^ traces (**A**), peak Ca^2+^ responses (**B**), and AUC for Ca^2+^ traces (**C**) in islet vessels of animals from CD (*n* = 4), WD (*n* = 4), and refed (*n* = 6) groups at week 48. (**D**) Average RBC velocity dynamics upon VEGFA injection and (**E**) AUC for RBC velocity excursions in islet vessels of animals from CD (*n* = 14), WD (*n* = 8), and refed (*n* = 10) groups at week 48. (**F**) Electron micrographs of islet grafts dissected from the ACE of CD, WD, and refed groups of animals at week 48. Scale bars: 5 µm (upper), 1 µm (lower). Asterisks indicate basement membrane, and arrows indicate fenestrae. (**G**–**I**) Basement membrane thickness (**G**), number of fenestrae (**H**), pore sizes of fenestrae (**I**), and percentage of capillaries with transcytotic structure (**J**) in islet vessels of CD (*n* = 14), WD (*n* = 9), and refed (*n* = 7) groups of animals at week 48. (**K** and **L**) Average dextran fluorescence intensity dynamics outside islet grafts upon injection in animals from CD (*n* = 5), WD (*n* = 7), and refed (*n* = 6) groups at week 48 (**K**) and AUC of dextran leakage within the first 3 minutes after injection (**L**). Data are shown as mean ± SEM (**A**, **D**, and **K**) or individual points (the rest). Statistics are based on 1-way ANOVA. *P < 0.05, **P < 0.01, ***P < 0.001, ****P < 0.0001, and NS (*P* > 0.05).

We proceeded with evaluation of islet vessel VEGF-A responsiveness and barrier function in the refed mice after 24 weeks of refeeding. Surprisingly, VEGF-A–triggered Ca^2+^ mobilization in islet endothelial cells of the refed mice remained almost as blunted as in the WD-fed mice (Figure 6A). Both peak amplitude of [Ca^2+^]_i_ and total Ca^2+^ response were considerably lower than those in CD-fed mice (Figure 6, B and C). Similarly, VEGF-A–induced islet blood flow fluctuations were also diminished in the refed group, with no significant improvement compared with the WD group (Figure 6, D and E). We then investigated islet vessel ultrastructural and barrier properties at the same time point. Electron micrographs derived from microdissected islet grafts showed drastic thickening of basement membrane due to excessive deposition of matrix proteins in both WD and refed groups of mice at 48 weeks (Figure 6, F and G). Islet vessels of the refed group had notably more fenestration than that of the WD group, but still less than the controls (Figure 6, F and H), while the sizes of the pores were indiscernible among all three groups (Figure 6I). Moreover, the compensatory formation of transcytotic vesicles was reduced by refeeding, possibly due to an increased degree of fenestration (Figure 6J). Consistent with our observations in islet grafts, in situ pancreatic islet vessels in the refed group also resembled the WD group, with a thickened basement membrane and reduced fenestration in comparison with the CD group (Supplemental Figure 6). In agreement with the above findings, islet vessel permeability to 3–5 kDa FITC-dextran in the refed group was slightly higher but not significantly different from that of the WD group, both of which were lower than the CD group (Figure 6, K and L).

### Irreversible islet vessel dysfunction impedes insulin outflow despite preserved β cell secretory capacity.

**Figure 7:**
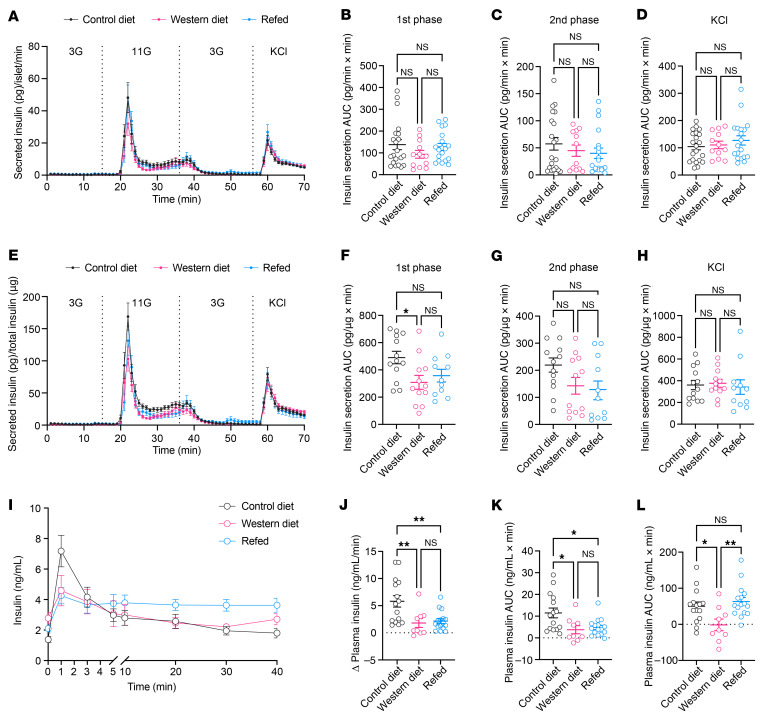
Irreversible islet vessel dysfunction impedes insulin release in vivo. (**A**–**D**) Dynamic insulin secretion from freshly isolated islets in a perifusion system, normalized to islet number (**A**), and AUC for glucose-induced first-phase (**B**), second-phase (**C**), and KCl-induced (**D**) insulin secretion. Islets were obtained from CD- (*n* = 5–9), WD-fed (*n* = 4), and refed animals (*n* = 4–8) at week 48. (**E**–**H**) Same analysis for dynamic insulin secretion as above but normalized to total insulin content. (**I**) Plasma insulin excursions during IVGTT in CD (*n* = 15), WD (*n* = 9), and refed (*n* = 16) groups of animals at week 48. (**J**) Increase in plasma insulin concentrations during the first minute of IVGTT in CD (*n* = 15), WD (*n* = 9), and refed (*n* = 16) groups of animals at week 48. (**K** and **L**) AUCs for insulin excursions during the first 3 minutes (**K**) and by the end of the tests (**L**) in CD (*n* = 15), WD (*n* = 9), and refed (*n* = 16) groups of animals at week 48. Data are shown as mean ± SEM (**A**, **E**, and **I**) or individual points (the rest). Statistics are based on 1-way ANOVA. *P < 0.05, **P < 0.01, and NS (*P* > 0.05).

We have demonstrated that islet vessel barrier dysfunction alone hinders insulin transport, aggravating glucose homeostasis in the WD-fed mice. To examine the impacts of dysfunctional islet vessels on glucose handling in the lean refed mice, we examined their insulin secretion dynamics ex vivo and in vivo. Freshly isolated islets from all three groups of mice were subjected to the same perifusion system as described before for measuring dynamic insulin release under glucose and KCl challenge. By the end of the diet regimen, average glucose- and KCl-induced insulin secretion per islet was comparable among all three groups (Figure 7, A–D). When normalized to total insulin content, however, islets from the WD-fed mice exhibited significantly decreased glucose-induced, first-phase insulin secretion in comparison with the control mice, while second-phase insulin secretion was also slightly lower. By contrast, islets from the refed mice secreted moderately, yet not significantly, less insulin under 11 mM d-glucose compared with the control mice, having largely preserved their secretion capacity (Figure 7, E–H). We then tested peripheral insulin sensitivity in all three groups of animals by conducting intraperitoneal and intravenous insulin tolerance tests. The refed mice performed in a similar way to the controls and exhibited a much higher insulin sensitivity than the WD-fed mice (Supplemental Figure 7, A–D). However, they were slightly less efficient in glucose clearance than the control mice during IVGTTs (Supplemental Figure 7, E and F). This was in line with their blunted in vivo insulin release following glucose injection, which resembled the WD-fed mice instead of the controls (Figure 7I). The elevation of plasma insulin concentration at 1 minute was considerably lower in the refed mice than the control mice (Figure 7J), and the amount of insulin that reached the bloodstream within the first 3 minutes was also markedly less (Figure 7K). The total amount of insulin that crossed the islet vessel barrier during the test was similar between the control and refed mice (Figure 7L). Finally, to eliminate the confounding peripheral factors that may obscure our interpretation of the insulin response in refed mice, we measured plasma c-peptide levels in another set of IVGTTs. Consistent with the insulin measurements, acute but not total c-peptide release was reduced in the refed mice compared with the controls (Supplemental Figure 7, G–J), suggesting that the sustained islet vessel barrier dysfunction indeed delayed the outflow of islet-derived hormones into the blood stream.

### Excessive glucose and free fatty acids impair VEGF-A–triggered VEGFR2 internalization and downstream signaling in endothelial cells through atypical PKC overactivation.

To identify the mechanisms behind the loss of VEGF-A responsiveness and pathogenesis of islet vessel dysfunction during WD feeding, we examined several factors that may affect the strength of VEGF-A signaling in islet endothelial cells. The soluble form of VEGF receptor 1 (sFlt-1) acts as a decoy receptor and binds the ligand at a much higher affinity than VEGFR2 (60). It is thus generally considered a prominent and natural antagonist to VEGF-A signaling. Average plasma sFlt-1 concentration in mice fed with WD for 24 weeks didn’t differ from that of mice fed with CD (Supplemental Figure 8A). Similarly, plasma sFlt-1 levels were also indistinguishable among CD, WD, and refed groups of mice by week 48 (Supplemental Figure 8B). We then excluded the possibility that a lack of VEGF-A sensitivity in the WD and refed groups of mice is due to excessive neutralization of VEGF-A ligand by circulating sFlt-1.

**Figure 8:**
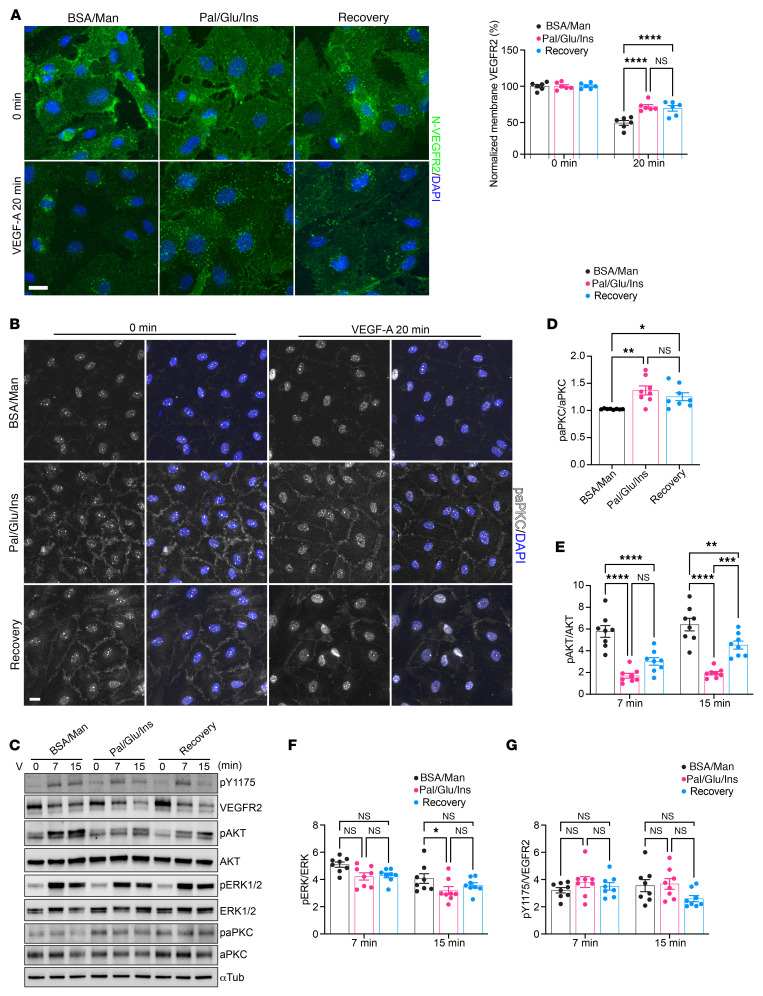
Components of WD increase endothelial cell aPKC activity and undermine VEGFR2 internalization and downstream signal transduction. (**A**) Left: cell surface immunofluorescence staining of HDMECs cultured under indicated conditions, showing membrane VEGFR2 abundance before and 20 minutes after VEGF-A stimulation. Right: quantification of relative fluorescence intensity from cell surface VEGFR2 staining in the above experiment (*n* = 3). (**B**) Immunofluorescence staining showing phosphorylated aPKC signals in HDMECs cultured under indicated conditions before and 20 minutes after VEGF-A stimulation. Scale bars: 20 µm. (**C**) Western blots showing VEGF-A–induced phosphorylation of downstream signaling molecules in HDMECs cultured under indicated conditions. (**D**) Normalized aPKC phosphorylation at baseline level before stimulation in HDMECs (*n* = 4). (**E**–**G**) Normalized AKT (**E**), ERK1/2 (**F**), and VEGFR2Y1175 (**G**) phosphorylation levels during VEGF-A stimulation in HDMECs cultured under indicated conditions (*n* = 4). Data are shown as individual points. Statistics are based on 1-way ANOVA (**D**) or 2-way ANOVA (**E**–**G**). *P < 0.05, **P < 0.01, ***P < 0.001, ****P < 0.0001, and NS (*P* > 0.05).

VEGFR2 internalization following ligand binding is essential for full activation of its kinase activity (12). This process involves the association of VEGFR2 to ephrin-B2 via the clathrin-associated sorting protein Disabled 2 (Dab2) and the cell polarity partitioning defective protein-3 (PAD-3) (61, 62). Formation of this complex is negatively regulated by atypical PKCs (aPKCs; PKCι and PKCζ) through direct phosphorylation of Dab2 (62). To model the vascular effects of overnutrition in WD-fed mice, we cultured human dermal microvascular endothelial cells (HDMECs) under diet-mimicking conditions for 6 days, using a mixture of 100 µM palmitate, 25 mM d-glucose, and 1 µM insulin (Pal/Glu/Ins). Control HDMECs were cultured with fatty acid free BSA and mannitol (BSA/Man) during the same period. The recovery group of cells was cultured under a diet-mimicking condition for 3 days followed by reversal to the control condition for another 3 days. We then set out to examine the pattern of VEGFR2 activation and signal transduction in these cells. Before VEGF-A stimulation, all cells displayed diffuse membrane-localized VEGFR2 staining. After 20 minutes of incubation with VEGF-A, VEGFR2 fluorescence in control cells became more punctate and the total membrane fluorescence intensity decreased markedly, indicative of receptor dimerization and internalization (Figure 8A). However, in the Pal/Glu/Ins and recovery groups, although receptor dimerization after VEGF-A stimulation was also evident, the decline in cell surface VEGFR2 fluorescence was not as striking, suggesting reduced VEGFR2 internalization upon activation (Figure 8A).

To elucidate the molecular basis for these observations, we accessed gene expression levels of *EFNB2*, *PARD3*, *DAB2*, *PRKCI*, and *PRKCZ* (encoding ephrin-B2, PAR-3, Dab2, PKCι, and PKCζ, respectively) in HDMECs. Pal/Glu/Ins treatment did not alter the expression of any of these genes compared with control conditions (Supplemental Figure 8C). We next asked whether aPKC hyperactivity was responsible for impaired VEGFR2 internalization under diet-mimicking conditions. Using immunofluorescence, we detected a low basal level of aPKC phosphorylation (paPKC) in control HDMECs, which was mainly confined to the nuclei. After 20 minutes of VEGF-A stimulation, the nuclear paPKC signal increased slightly (Figure 8B). By contrast, HDMECs in the Pal/Glu/Ins and recovery groups exhibited a much stronger paPKC signal at the plasma membrane and junctional region, both before and after VEGF-A stimulation, while nuclear aPKC activity remained comparable with that of control cells (Figure 8B). Thus, Pal/Glu/Ins treatment increased aPKC activity in specific subcellular compartments, which persisted even after stressor removal, potentially hampering VEGFR2 internalization. We further examined VEGF-A–induced phosphorylation of protein kinase B (AKT) and mitogen-activated protein kinase ERK1/2 (Figure 8C). In agreement with the above findings, the Pal/Glu/Ins and recovery groups showed elevated baseline aPKC phosphorylation at 0 minutes (Figure 8, C and D). As a result, VEGF-A–induced AKT phosphorylation was substantially reduced in both groups compared with control cells, while ERK1/2 phosphorylation was affected to a lesser extent (Figure 8, C, E, and F). Autophosphorylation of VEGFR2 at Tyr1175 was similar under all three culture conditions (Figure 8G).

**Figure 9:**
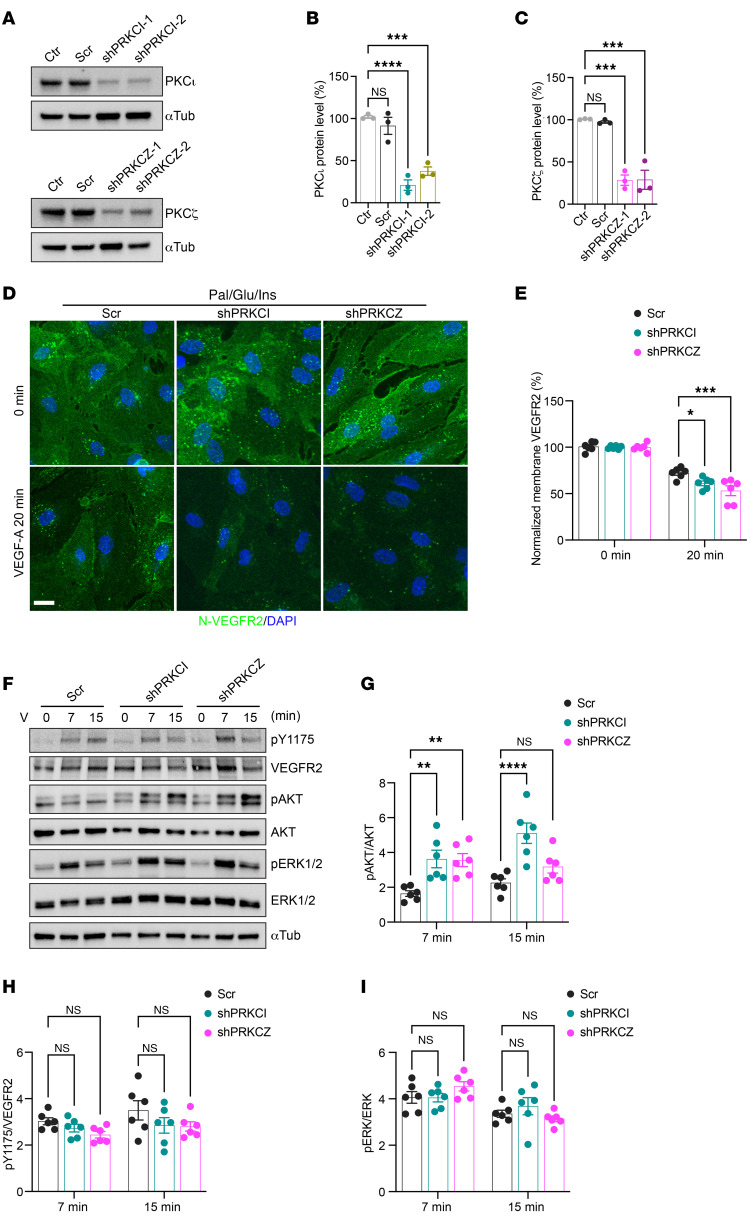
aPKC knockdown restores VEGF-A–induced VEGFR2 internalization and AKT phosphorylation under diet-mimicking conditions. (**A**) Specific knockdown of aPKC isoforms by shRNAs in HDMECs. (**B** and **C**) Quantification of aPKC silencing efficiency in comparison with nontransduced cells (Ctr) and cells transduced with scrambled sequence (Scr) (*n* = 3). (**D**) Immunofluorescence staining of shRNA-transduced HDMECs cultured under diet-mimicking conditions, showing cell surface VEGFR2 abundance before and 20 minutes after VEGF-A stimulation. Scale bar: 20 µm. (**E**) Quantification of relative fluorescence intensity from cell surface VEGFR2 staining (*n* = 3). (**F**) Western blots showing VEGF-A–induced phosphorylation of downstream signaling molecules in shRNA-transduced HDMECs cultured under diet-mimicking conditions. (**G**–**I**) Normalized AKT (**G**), VEGFR2Y1175 (**H**), and ERK1/2 (**I**) phosphorylation levels during VEGF-A stimulation in shRNA-transduced HDMECs cultured under diet-mimicking conditions (*n* = 3). Data are shown as individual points. Statistics are based on 1-way ANOVA (**B** and **C**) or 2-way ANOVA (the rest). *P < 0.05, **P < 0.01, ***P < 0.001, ****P < 0.0001, and NS (*P* > 0.05).

To verify the role of aPKC in VEGF-A signaling impairment, we selectively silenced aPKC isoforms by shRNAs in HDMECs and subjected them to the diet-mimicking conditions (Figure 9, A–C). In comparison with control cells transduced with scrambled shRNA, aPKC-silenced cells exhibited significantly enhanced VEGF-A–induced receptor internalization under the Pal/Glu/Ins condition (Figure 9, D and E). Consistently, AKT phosphorylation was also markedly higher in aPKC-silenced cells than in scrambled controls (Figure 9, F and G), while VEGFR2Y1175 and ERK1/2 phosphorylation were unaffected (Figure 9, H and I).

Prolonged WD feeding is known to induce a buildup of ROS in various cell types, which can disrupt intracellular signaling (63). We thus tested whether elevated ROS levels under the diet-mimicking conditions contributed to VEGF-A/VEGFR2 signaling impairments in HDMECs. Cells cultured under the Pal/Glu/Ins condition accumulated a markedly increased level of ROS, as indicated by the fluorescent ROS sensor carboxy-H_2_DFFDA. This was effectively blocked by the ROS scavenger *N*-acetyl-d-cysteine (NAC) (Supplemental Figure 8, D and E). We then examined the effect of ROS inhibition on VEGF-A–triggered VEGFR2 signaling under the same condition. While VEGFR2 internalization remained diminished in the presence of NAC, it was restored by a specific inhibitor to aPKCs, 2-acetyl-cyclopentane-1,3-dione (ACPD) (Supplemental Figure 9, A and B). Accordingly, aPKC hyperactivity was reversed, and AKT phosphorylation was rescued by ACPD but not NAC (Supplemental Figure 9, C–E). Neither NAC nor ACPD had any effect on VEGFR2 or ERK1/2 phosphorylation (Supplemental Figure 9, F and G). These results argue that aPKC hyperactivity, rather than increased intracellular ROS levels, is a primary driver of the impaired VEGFR2 internalization and downstream signaling observed in our model.

### VEGFR2 downstream signaling is compromised in pancreatic islet endothelial cells of WD-fed and refed mice.

**Figure 10:**
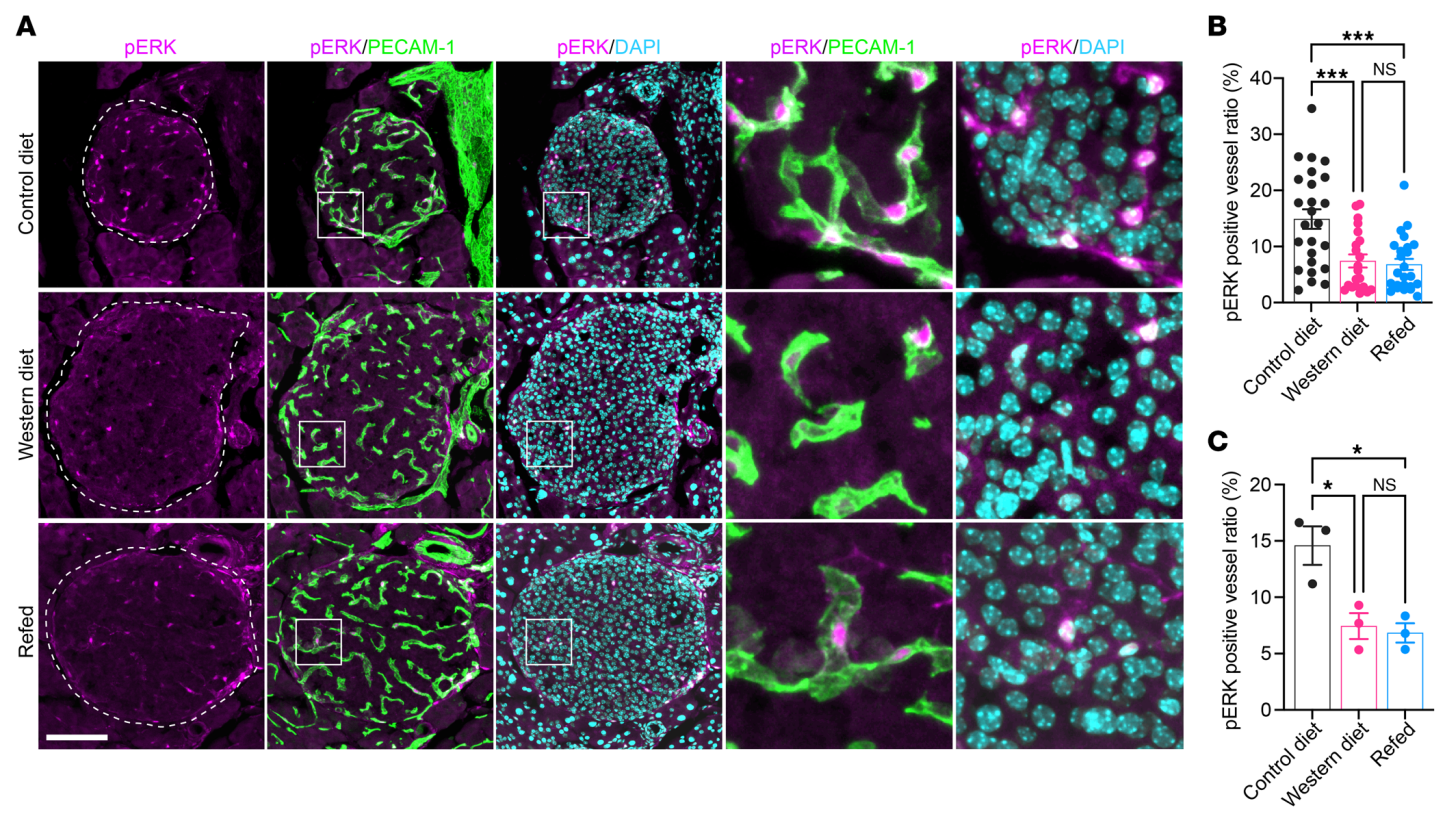
VEGF-A–induced ERK1/2 activation is blunted in pancreatic islet endothelial cells of WD-fed and refed mice. (**A**) Immunofluorescence staining of pancreatic sections from CD, WD, and refed groups of animals at week 48, showing ERK1/2 phosphorylation (magenta) in islet vessels triggered by intravenous VEGF-A injection. PECAM-1 signals are shown in green and DAPI in cyan. Squares indicate areas magnified in the respective panels on the right side. Scale bar: 100 µm. (**B** and **C**) Relative area of islet vessels that are positive for pERK1/2 staining in pancreatic sections from CD, WD, and refed groups of animals at week 48 (*n* = 3 for animals). (**B**) shows quantifications from all vessel segments, and (**C**) shows the average ratio per mouse. Data are shown as individual points, and statistics are based on 1-way ANOVA. *P < 0.05, ***P < 0.001, and NS (*P* > 0.05).

To validate our findings in situ, whole pancreata of mice in CD, WD, and refed groups at week 48 were harvested 6 minutes after an intravenous injection of VEGF-A and fixed immediately to capture the phosphorylation status of various signaling molecules downstream of VEGFR2. Immunofluorescence staining of pancreatic sections from CD-fed mice exhibited robust ERK1/2 phosphorylation and nuclear localization, whereas pERK1/2 fluorescence intensity and the percentage of pERK1/2 positive islet vessel area were visibly reduced in both WD-fed and refed mice (Figure 10, A–C). Thus, long-term WD consumption leads to irreversible VEGF-A desensitization and diminished downstream signal transduction in native pancreatic islet vessels, consistent with our observations in the islet grafts transplanted into the eye.

## Discussion

Vascular cells are highly susceptible to oxidative stress caused by disrupted energy metabolism. The consequential damage in various vascular beds manifests as tissue-specific vascular diseases that have become the leading cause of morbidity and mortality among individuals with metabolic disorders. While diabetes-related vessel abnormalities in insulin-sensitive tissues have been well documented (28, 64), the impact of obesity on islet vessels remains relatively unappreciated, with existing knowledge mostly limited to morphological characterizations. Previous studies using different animal models have reported extensive islet vascular remodeling associated with metabolic disorders (6, 7). Here, we described a progressive perturbation of islet vessel homeostasis in a WD-induced obese mouse model, identifying not only morphological alterations, but also islet endothelial cell desensitization to VEGF-A, compromised vascular barrier function, and hemodynamic dysregulation in vivo. Importantly, we also provided evidence for the adverse effects of dysfunctional islet vessels on glucose homeostasis by limiting insulin outflow into the systemic circulation.

In addition to the emergence of metabolic disorders in WD-fed mice, we detected substantial morphological alterations and functional disorders in islet vessels after 12 weeks of WD feeding, which deteriorated as the diet intervention continued. However, as islet vessels are not among the earliest responders to WD, there may be a critical period for early intervention to curb the pathogenesis of obesity-related vascular complications. Intriguingly, our investigations also revealed considerable heterogeneity among islet vascular cells under metabolic stress, as evidenced by uneven islet vessel enlargement and variable Ca^2+^ responses from individual capillary segments in WD-fed animals. While the phenotypic and functional heterogeneity across vascular beds in different organs is established (65, 66), and genetic signatures in capillary endothelial cells are shown to exhibit a clear tissue-specific pattern (37, 67, 68), further analysis is required to elucidate the molecular basis for such intra-islet vessel heterogeneity to identify potential subpopulations of vascular cells that are more susceptible to obesogenic diets.

Sustained islet vessel dysfunction was observed not only in the WD-fed mice, but also in the refed mice. Although replacing WD with CD rapidly alleviated their metabolic disorders, islet vasculature in refed mice still resembled that of the WD-fed mice, characterized by enlarged vessel diameters. In addition, islet vessels in the refed mice exhibited only partial recovery of fenestration and minimal improvement in permeability, while diminished intracellular Ca^2+^ mobilization in response to VEGF-A, increased basement membrane thickness, and hemodynamic dysregulation persisted after 24 weeks CD feeding. By the end of the study, none of the measured parameters were fully restored to the control levels, implying a persistent memory effect of the dietary stressors on islet vessels. The concept of metabolic memory, whereby vascular endothelial cells retain cellular imprints generated by previous metabolic stress despite removal of the stressors, has been reported in macro- and microvessels of T2D patients (69–71). Mechanisms proposed to explain this memory in endothelial cells include intracellular accumulation of ROS and advanced glycation end products, both of which can lead to increased oxidative stress, mitochondria damage, and epigenetic modifications (72, 73). Although our results rule out a direct role of ROS in VEGF-A desensitization, we observed markedly elevated ROS in endothelial cells under diet-mimicking conditions, suggesting potential broader implications for vascular dysfunction. Given the cumulative nature of this memory effect, our findings underscore the importance of early intervention to preserve islet vessel function under pathological conditions such as obesity. It is thus likely that the reversibility of vessel pathogenesis would have been higher if we had conducted the diet switch at an earlier point during the time course. Additionally, in light of reported sex differences in their response to dietary stressors (74, 75), it would be valuable to investigate whether female mice display greater resilience in islet vessel function following WD exposure or increased reversibility of VEGF-A desensitization and related vascular dysfunction.

Anatomically interposed between the endocrine cells and the bloodstream, islet vessels are not merely passive conduits of nutrients and oxygen. Instead, they actively contribute to nutrient sensing and hormone secretion, readily adapting the barrier functions and vascular tone in response to islet metabolic activity (76, 77). This functional coupling may become disrupted in metabolic disorders due to the morphological and functional modifications in islet vessels. Indeed, although β cell secretory capacity remained intact in mice fed with WD for 12 weeks, plasma insulin and c-peptide excursions following an intravenous glucose bolus were blunted in the same groups of animals. In intraperitoneal glucose tolerance tests (IPGTTs), glucose sensing by β cells may be influenced by inadvertent injection into visceral fat, variable glucose diffusion rates, and absorption rates by peritoneal capillaries (78). Intravenous administration of glucose ensures rapid and accurately controlled delivery of glucose to islets directly through their native vasculature, which allows us to address the role of islet vessels in glucose homeostasis. A dysfunctional islet vessel barrier in WD-fed mice impeded the acute release of insulin and c-peptide into circulation, although they had a mildly increased intra-islet vascular density. Of note, these secreted peptides were not retained in the islets of WD-fed mice, as the cumulative amounts released by the end of the test were similar between the two groups. Furthermore, the lean refed mice continued to display mild glucose intolerance and reduced plasma insulin/c-peptide peaks compared with control mice during IVGTTs, even though their body weight, insulin sensitivity, and insulin secretion capacity had normalized. Collectively, these results demonstrate the adverse impact of disrupted islet vessel function on glucose homeostasis. If islet vascular dysfunction is not addressed during the early phase of pathogenesis, it may exacerbate the metabolic outcomes at later stages, such as β cell failure in diabetes.

VEGF-A/VEGFR2 signaling is a key modulator of islet vascular morphology and function. Both hypo- and hypervascularization of islets caused by manipulation of islet VEGF-A expression level undermine glucose metabolism (13, 15, 16). It is not only noteworthy that islet endocrine cells produce VEGF-A at a higher level than the exocrine pancreas, but also that the expression level of VEGFR2 in islet vessels is higher than that of the surrounding acinar vessels, consistent with previous documentation both in mice and human (13, 37). This implies that islet vessel functionality may be highly susceptible to perturbations in VEGF-A/VEGFR2 signaling. Our studies revealed that islet expression and production of VEGF-A increased after 8 to 12 weeks of WD feeding, whereas islet endothelial cell responsiveness to VEGF-A declined almost simultaneously. Gene expression analysis and immunofluorescence staining confirmed the abundance of VEGFR2 on the islet endothelial cell membrane, suggesting that continuous WD feeding compromised receptor signaling rather than its expression. Along the same line, loss of islet endothelial cell VEGF-A sensitivity persisted after 24 weeks of refeeding, although islet expression and production of VEGF-A was lowered to the control levels. Encouragingly, recent human studies also identified disruptions in the islet VEGF-A/VEGFR2 pathway in T2D (37), providing a potential target for therapeutic strategies. Meanwhile, it is important to recognize that disruptions in VEGF-A/VEGFR2 signaling in diabetes exhibit tissue-specific patterns, and VEGF-A resistance is not uniformly present across all vascular beds. For example, in proliferative diabetic retinopathy, excessive angiogenesis and vascular leakage are driven by hyperactive VEGF-A signaling as a result of increased ligand production (32).

Previous studies have demonstrated that aPKCs negatively regulate VEGFR2 signaling by inhibiting its internalization (62). aPKCs are implicated in metabolic disorders due to their unique activation by anionic phospholipids and sphingolipids, such as phosphatidylinositol, phosphatidic acid, and ceramide (79–82). Additionally, insulin also activates aPKCs in peripheral tissues, and dysregulated aPKC activities occur in a tissue-specific manner in T2D (83–85). In our model, prolonged WD feeding likely promotes intracellular accumulation of these lipids and alters the membrane lipid profile in endothelial cells (86, 87). Supporting this, we detected enhanced aPKCs activity specifically at cell membranes and junctions in HDMECs under diet-mimicking conditions. This led to diminished VEGFR2 internalization and reduced downstream AKT phosphorylation, which were effectively reversed by genetic ablation or pharmacological inhibition of aPKCs. Intriguingly, elevated aPKC phosphorylation and impaired VEGFR2 signaling remained evident in the recovered HDMECs after the removal of dietary stressors, potentially explaining the sustained endothelial desensitization to VEGF-A observed in vivo. Given that lipid-induced aPKC activation is normally acute (79, 88), it is plausible that intracellular lipid abnormalities and potential modifications of other upstream signals persisted and continued to overactivate aPKC even after diet normalization. Nevertheless, we acknowledge that assessing basal phosphorylation level of aPKC in intact pancreatic islets is technically challenging. As the detailed mechanisms underlying aPKC dysregulation in islet endothelial cells remain undefined in our study, further investigations are necessary to delineate the upstream signaling components modulating aPKC activity in response to WD consumption. Additionally, it is essential to determine whether metabolic disorder–associated aPKC hyperactivity observed in our mouse model is recapitulated in human islet vessels, which will be critical for the development of targeted strategies for islet vascular protection.

Besides the high dietary sugar and fat intake, other risk factors may also account for islet vessel dysfunction in our model of long-term WD feeding. Aging, for instance, is associated with endothelial cell senescence, dysfunction, and declined VEGF-A signaling activity. Recent evidence demonstrates reduced VEGF-A signaling activity in multiple organs of aged mice due to an increased production of sFlt-1 (89). In our study, we observed a modest, time-dependent decrease in VEGF-A–triggered Ca^2+^ mobilization in the CD-fed mice, suggesting aging-related impairment. Therefore, ageing may have acted synergistically with metabolic stress to exacerbate islet VEGF-A desensitization and vessel dysfunction in WD-fed mice. Future studies using our versatile transplantation platform will allow isolation of the aging factor and clarify the sole impacts of islet vascular ageing on its function, e.g., by mismatching the ages of islet donors and recipients.

While we focused on alterations in islet endothelial VEGF-A signaling activity and related vessel function, accessing the plasticity and heterogeneity of other vascular cell types under WD feeding could provide further insights into its overall vascular impacts. Islet pericyte dysfunction, for instance, may contribute to WD-induced islet vessel stiffness and ultrastructural modifications, as they participate in the deposition of certain matrix proteins in the islet basement membrane and constitute another element in the fine-tuning of islet blood flow (90, 91). In addition, although we have demonstrated that the vascular network of islets transplanted into the ACE closely mirrors native pancreatic islet vessels, as evidenced by their morphological similarities and parallel ultrastructural modifications under dietary stress (36, 58), this transplantation site does not fully recapitulate the pancreatic microenvironment, as it lacks direct exocrine and gastrointestinal inputs that also influence islet function.

Another limitation of our study is the use of a mixture of fentanyl, fluanisone, and midazolam as anesthesia, which have moderate blood pressure–lowering effects and thus may potentially influence our vivo measurements of islet blood flow (92). Nevertheless, compared with other commonly used anesthetics such as isoflurane, this combination exerts minimal effects on insulin secretion and has been validated for various types of vascular measurements (93, 94).

In summary, we characterized the progressive impairment of islet vascular function and its metabolic consequences in a WD-induced obese mouse model and identified intra-islet VEGF-A/VEGFR2 signaling obstruction as a key underlying mechanism. By examining the reversibility of islet vascular defects following diet normalization, we revealed the role of islet vessel dysfunction in the manifestation of diabetic phenotypes in obesity. This emphasized the necessity of maintaining physiological activity of intra-islet VEGF-A signaling for optimal islet vessel function and glucose homeostasis.

## Methods

### Sex as a biological variable.

Male mice were used for all experiments because of their susceptibility to diet-induced obesity and generally stronger phenotypes. Our findings are expected to be relevant for both sexes. Data from both male and female human donors were included in the gene expression assay.

### Mouse experiments.

Detailed mouse experiments and in vitro experimental procedures are described in Supplemental Methods.

### Statistics.

All results are presented as mean ± SEM (with individual data points for bar graphs). Conventional 2-way ANOVA or 2-way repeated-measure ANOVA tests were used for time-course analysis where appropriate. One-way ANOVA tests were used for comparison of multiple groups. Two-tailed Student’s *t* tests or Mann-Whitney tests were used for 2-group comparisons where appropriate. A *P* value of less than 0.05 was considered to indicate significance (GraphPad Prism 9).

### Study approval.

Experimental procedures involving live animals and human islets were carried out in accordance with the Karolinska Institutet’s guidelines for the care and use of animals in research and were approved by the institute’s Animal Ethics Committee (ethical permit numbers 19462-2017 and 18526-23).

### Data availability.

Values for data points in all graphs are reported in the Supporting Data Values file.

## Author contributions

YX coordinated and designed the study. YX, EI, and POB conceived the study. EI and POB supervised the study. YX, AD, MV, and EI performed the experiments and analyzed the results. YX wrote the manuscript. EI and POB revised and edited the manuscript. All authors reviewed the results and approved the final version of the manuscript.

## Supplementary Material

Supplemental data

Unedited blot and gel images

Supplemental video 1

Supplemental video 2

Supplemental video 3

Supporting data values

## Version 1 06/09/2025

In-Press Preview

## Version 2 08/01/2025

Electronic publication
