# Supplementary material for: Diet-induced obesity promotes endothelial cell desensitization to VEGF-A and permanent islet vessel dysfunction in mice
Source: J Clin Invest. 2025 Jun 5;135(15):e177601. doi: 10.1172/JCI177601 (PMC12321391; doi:10.1172/JCI177601)
Supplement: Supplemental data [file jci-135-177601-s147.pdf]

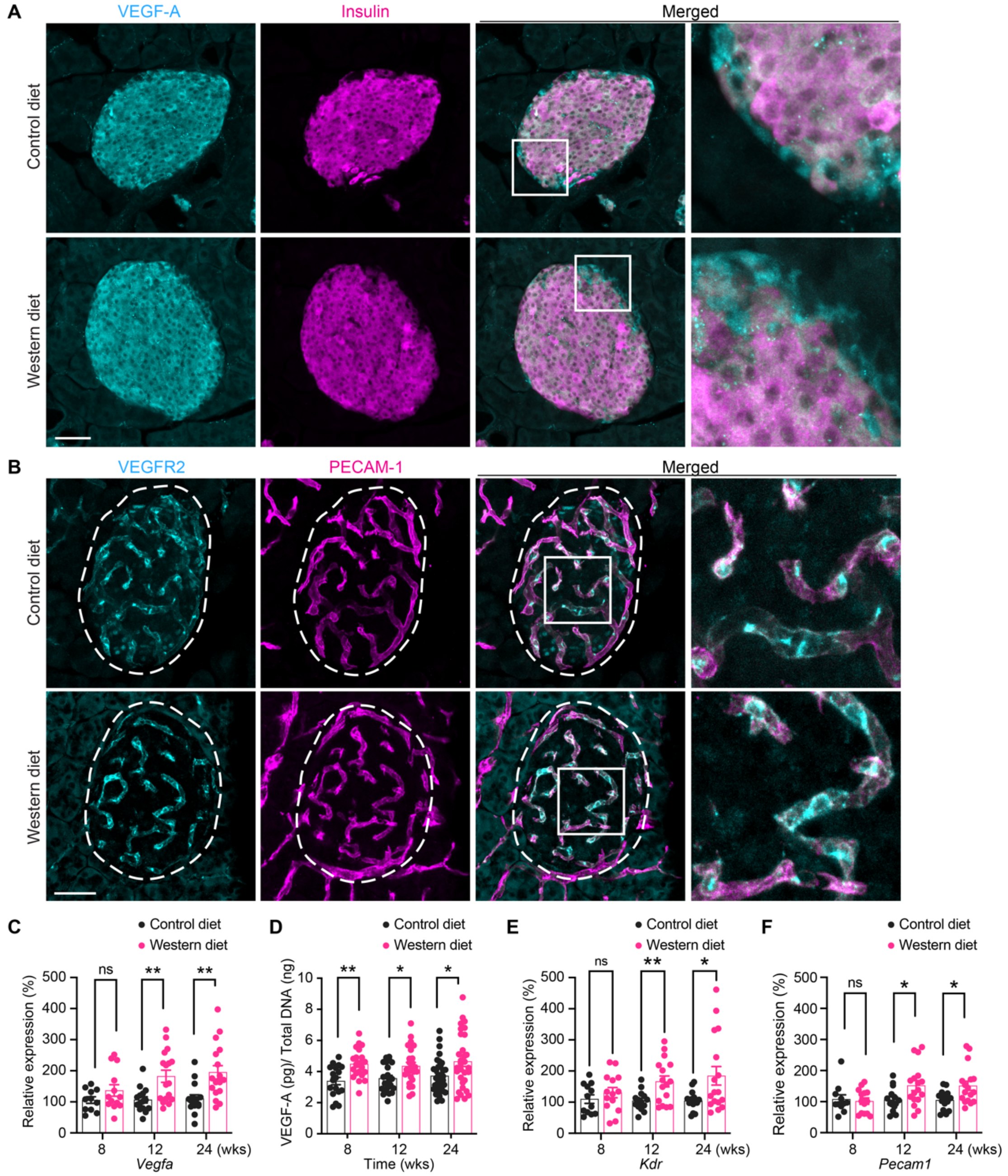

Supplemental Figure 1. **WD increases islet expression and production of VEGF-A.**
(**A-B**) Representative confocal images are presented as maximum intensity projections, showing immunofluorescence staining of pancreatic sections from CD- and WD-fed animals after 8 weeks of diet intervention. (**A**) VEGF-A staining is shown in cyan and insulin in magenta. (**B**) VEGFR2 is shown in cyan and PECAM-1 in magenta. Squares indicate areas magnified in the right panels. Scale bars: 50 µm. (**C**) Relative gene expression level of *Vegfa* in freshly isolated islets from CD- and WD-fed animals after 8, 12 and 24 weeks of diet intervention (CD: n=11, 15, 15; WD: n=13, 17, 17). (**D**) Ex vivo VEGF-A production in islets from CD- and WD-fed animals after 8, 12 and 24 weeks of diet intervention (CD: n=6, 6, 8; WD: n=6, 8, 8). (**E-F**) Relative gene expression levels of *Kdr* (**E**) and *Pecam1* (**F**) in freshly isolated islets from CD- and WD-fed animals after 8, 12 and 24 weeks of diet intervention (CD: *Kdr*, n=12, 14, 15; *Pecam1*, n=11, 15, 14; WD: *Kdr,* n=14, 16, 17; *Pecam1*, n=14, 17, 17). Data are shown as individual points. Statistics are based on multiple unpaired, two-tailed student's *t*-tests, *$p<0.05$, **$p<0.01$ and ns (*$p>0.05$).

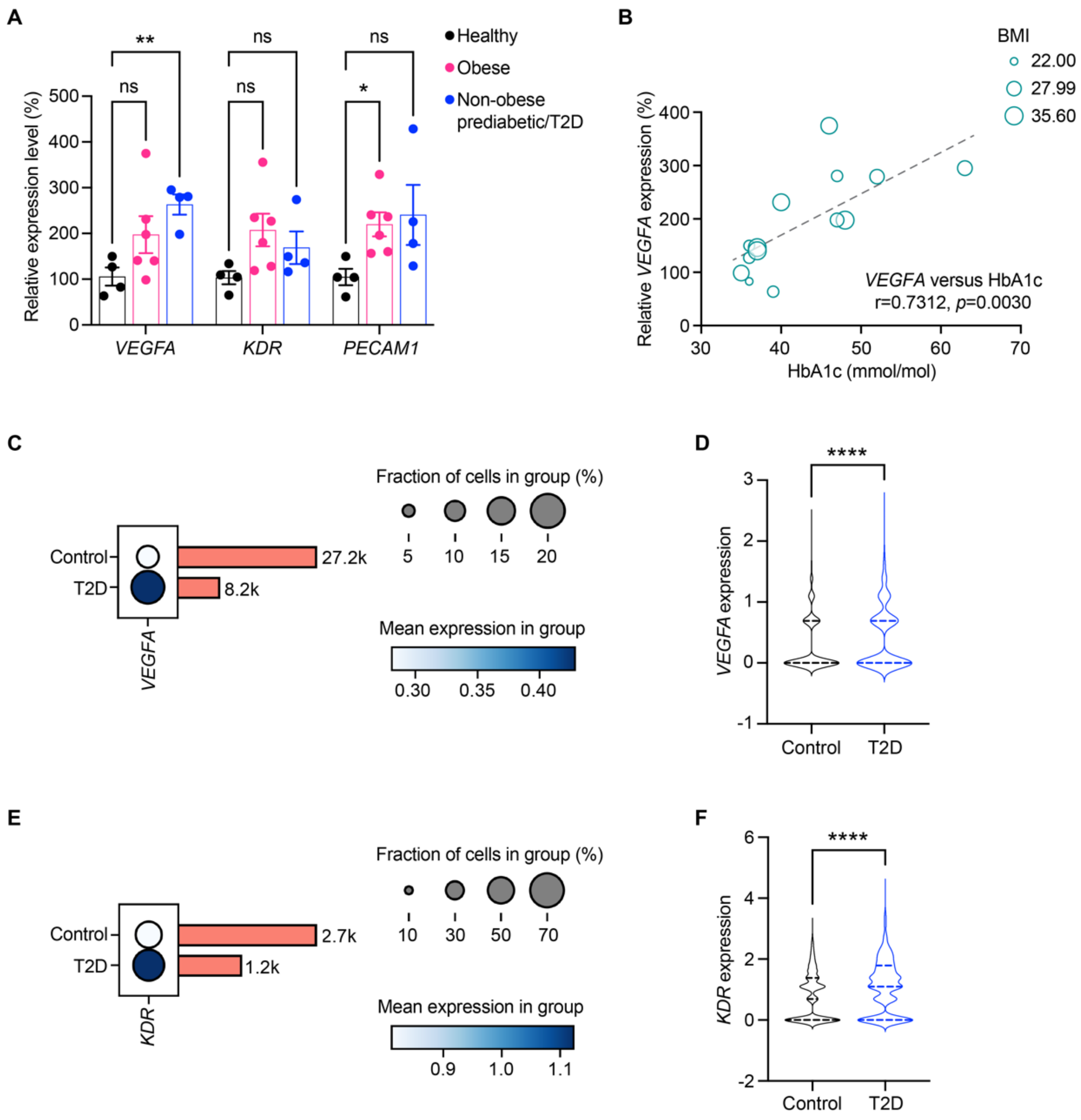

Supplemental Figure 2. ***VEGFA* and *KDR* are dysregulated in human pancreatic islets in metabolic disorders.**
(**A**) Relative gene expression levels of *VEGFA, KDR* and *PECAM1* in human islets from healthy (n=4), obese (n=6), prediabetic (n=3) and T2D (n=1) donors. (**B**) Correlation between the levels of *VEGFA* gene expression and plasma HbA1c in all human donors. Bubble sizes are relative to individual BMIs. (**C-D**) Extracted HPAP gene expression data of *VEGFA* in pancreatic β cells from healthy control (n=27.2k) and T2D donors (n=8.2k), showing fraction of cells, mean expression levels (**C**) and individual expression levels (**D**). (**E-F**) Extracted HPAP gene expression data of *KDR* in pancreatic islet endothelial cells from healthy control (n=2.7k) and T2D donors (n=1.2k), showing fraction of cells, mean expression levels (**E**) and individual expression levels (**F**). Data are shown as individual points (**A** and **B**) or violin plots with quartiles indicated by dashed lines in each group (**D** and **F**). Statistics are based on one-way ANOVA (**A**) or Mann-Whitney tests (**D** and **F**), *$p<0.05$, **$p<0.01$, ****$p<0.0001$ and ns (*$p>0.05$).

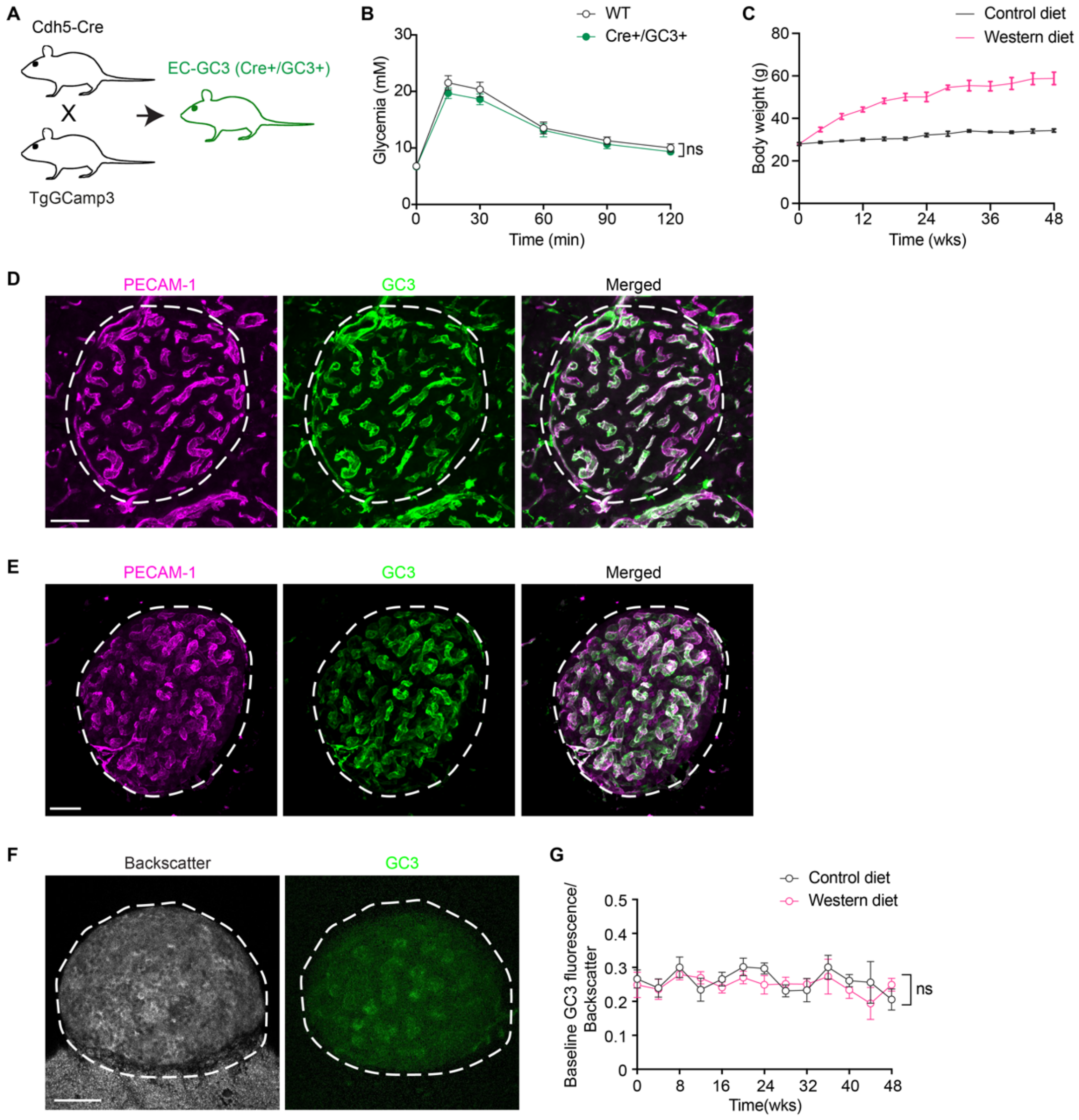

Supplemental Figure 3. Characterization of the EC-GC3 reporter mouse line.
(**A**) Generation of the EC-GC3 reporter mouse. (**B**) IPGTT of EC-GC3 (n=14) and their wild type littermates (WT, n=15). (**C**) Body weight changes during diet intervention in CD-(n=9-15) and WD-fed (n=9) EC-GC3 mice. (**D**-**E**) Representative confocal images are presented as maximum intensity projections, showing immunofluorescence staining of pancreatic sections (**D**) and whole-mount islet grafts (**E**) dissected from EC-GC3 animals. PECAM-1 staining is shown in magenta and GC3 (GFP) is shown in green. (**F**) Representative confocal images are presented as maximum intensity projections, showing backscatter signal (left) and baseline GC3 fluorescence (right) in an islet graft prior to intravenous VEGF-A injection. (**G**) Normalized baseline GC3 fluorescence fluctuations throughout the diet regimen in CD- (n=8-11) and WD-fed (n=8-15) EC-GC3 animals. Scale bars: 50 µm. Data are shown as mean ± SEM. Statistics are based on two-way ANOVA, ns (*p>0.05).

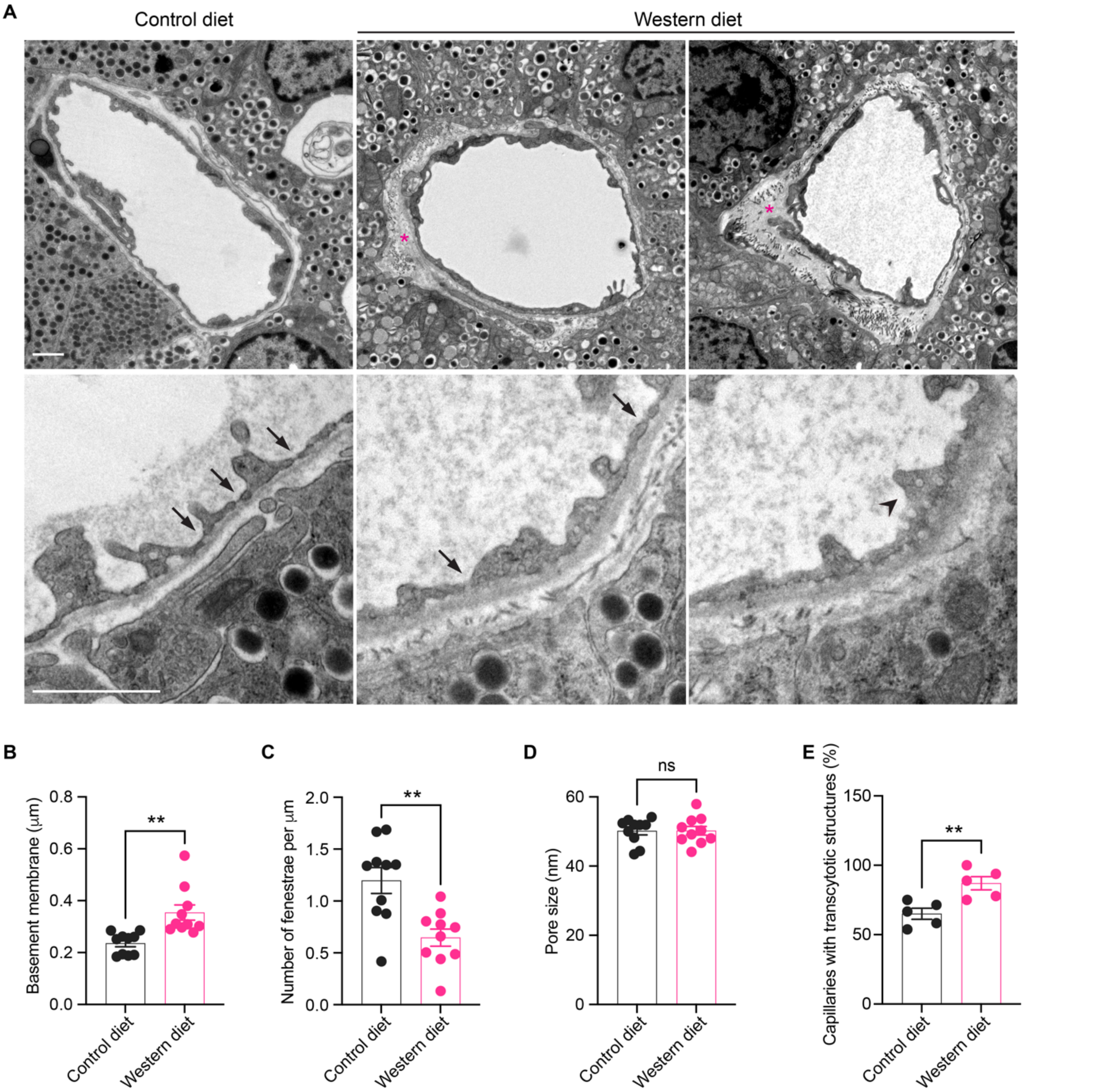

Supplemental Figure 4. Ultrastructural alterations in pancreatic islet vessels elicited by WD consumption by week 12.

(**A**) Electron micrographs of pancreatic islet samples from CD- and WD-fed animals at week 12. Scale bars: 1 µm. (**B-E**) Basement membrane thickness (**B**), number of fenestrae (**C**), pore sizes of fenestrae (**D**) and percentage of capillaries with transcytotic structure (**E**) in pancreatic islet vessels of CD- (n=5) and WD-fed (n=5) animals at week 12. Data are shown as individual points. Statistics are based on unpaired, two-tailed student's *t*-tests, $**p<0.01$ and ns ($*p>0.05$).

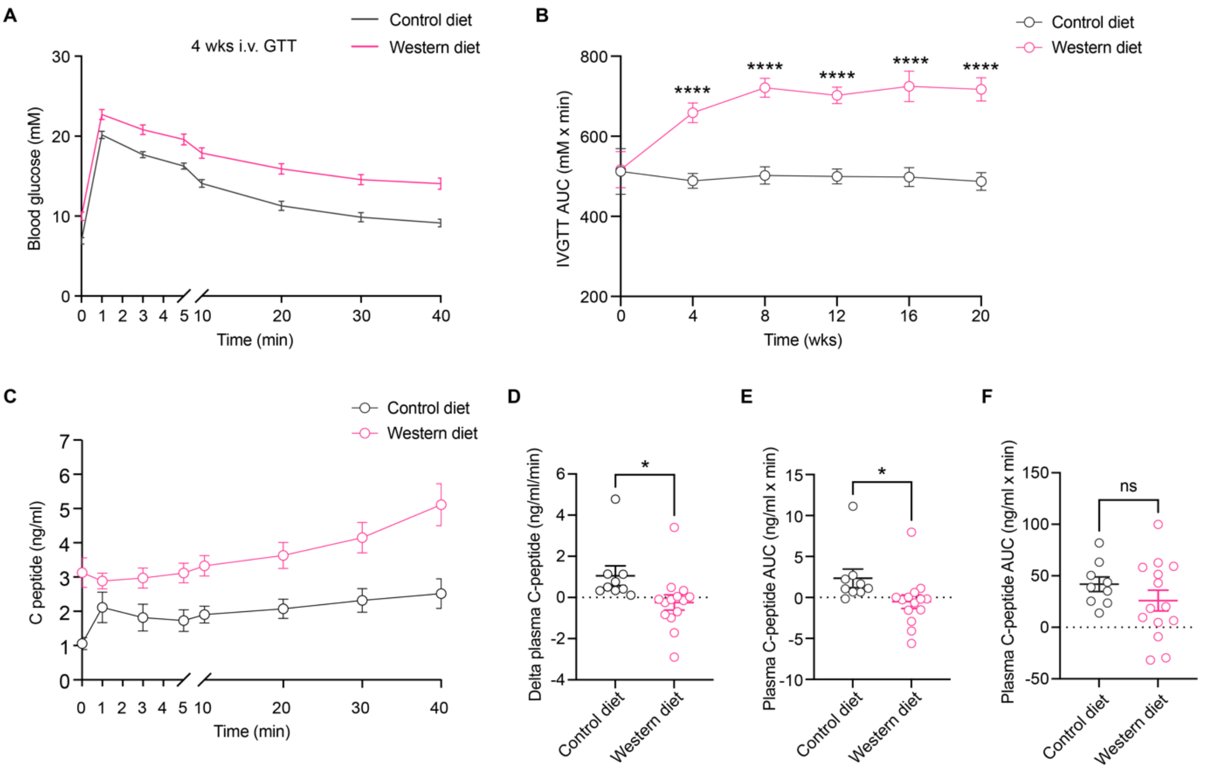

Supplemental Figure 5. WD-fed mice show impaired glucose clearance and c-peptide secretion during IVGTTs

(**A**) IVGTT in CD- (n=18) and WD-fed (n=18) animals at week 4. (**B**) AUC for IVGTTs in CD- (n=7-18) and WD-fed (n=6-23) animals from week 0 to week 20. (**C**) Plasma c-peptide excursions during IVGTT in CD- (n=9) and WD-fed (n=14) animals at week 12. (**D**) Increase in plasma c-peptide concentrations during the first minute of IVGTT in CD- (n=9) and WD-fed (n=14) animals at week 12. (**E-F**) AUCs for c-peptide excursions during the first 3 minutes (**E**) and by the end of the test (**F**) in CD- (n=9) and WD-fed (n=14) animals at week 12. Data are shown as mean ± SEM (**A-C**) or individual points (**D-F**). Statistics are based on one-way ANOVA (**B**) or unpaired, two-tailed student's *t*-tests (**D-F**), *$p<0.05$, ****$p<0.0001$ and ns (*$p>0.05$).

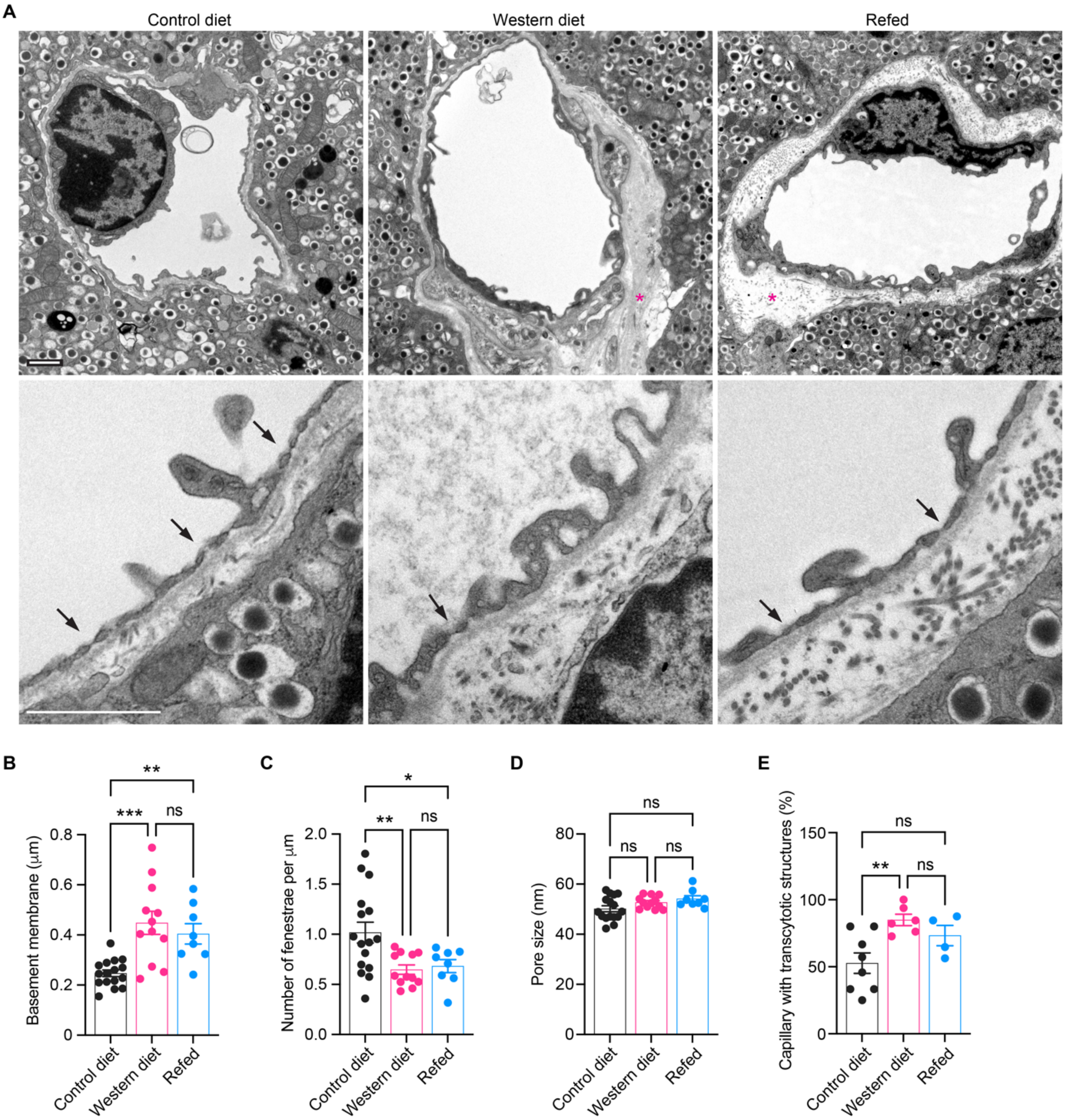

Supplemental Figure 6. Ultrastructural alterations in pancreatic islet vessels in WD-fed and refed mice by the end of diet regimen.

(**A**) Electron micrographs of pancreatic islet samples from CD-, WD-fed and refed animals at week 48. Scale bars: 1 µm. (**B-E**) Basement membrane thickness (**B**), number of fenestrae (**C**), pore sizes of fenestrae (**D**) and percentage of capillaries with transcytotic structure (**E**) in pancreatic islet vessels of CD (n=8), WD (n=6) and refed (n=4) groups of animals at week 48. Data are shown as individual points. Statistics are based on one-way ANOVA, *p<0.05, **p<0.01, ***p<0.001 and ns (*p>0.05).

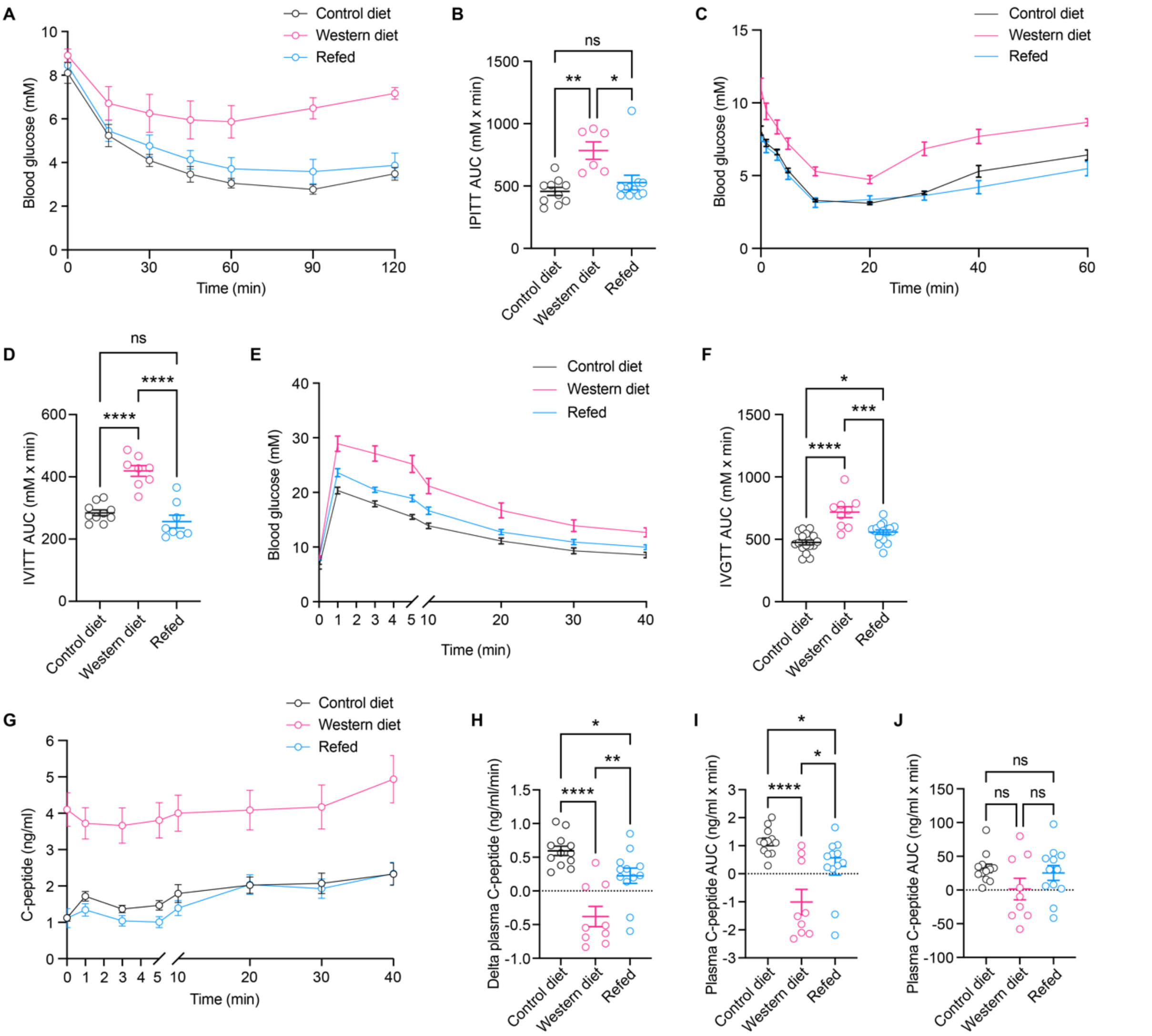

Supplemental Figure 7. Insulin sensitivity recovers while glucose clearance and c-peptide outflow remain compromised in refed animals.

(**A-B**) Intraperitoneal insulin tolerance test (IPITT) in animals from CD (n=10), WD (n=6) and refed (n=11) groups of animals at week 48 (**A**) and AUC for the test (**B**). (**C-D**) Intravenous insulin tolerance test (IVITT) in animals from CD (n=10), WD (n=8) and refed (n=8) groups of animals at week 48 (**C**) and AUC for the test (**D**). (**E-F**) IVGTT in animals from CD (n=16), WD (n=9) and refed (n=17) groups at week 48 (**E**) and AUC for the test (**F**). (**G**) Plasma c-peptide excursions during IVGTT in CD- (n=12), WD-fed (n=9) and refed (n=12) animals at week 48. (**H**) Increase in plasma c-peptide concentrations during the first minute of IVGTT in CD- (n=12), WD-fed (n=9) and refed (n=12) animals at week 48. (**I-J**) AUCs for c-peptide excursions during the first 3 minutes (**I**) and by the end of the test (**J**) in CD- (n=12), WD-fed (n=9) and refed (n=12) animals at week 48. Data are shown as mean ± SEM (**A**, **C**, **E** and **G**) or individual points (the rest). Statistics are based on one-way ANOVA, *p<0.05, **p<0.01, ***p<0.001, ****p<0.0001 and ns (*p>0.05).

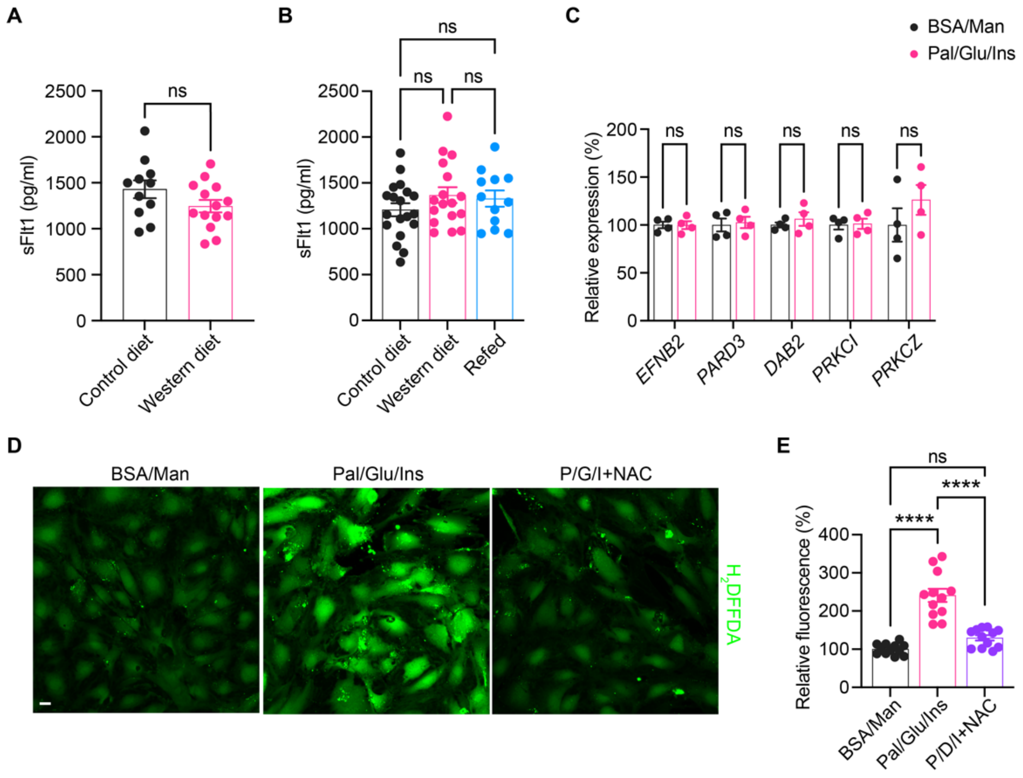

Supplemental Figure 8. Assessment of potential factors underlying impaired endothelial cell VEGFR2 signaling activity caused by WD elements.

(**A**) Plasma sFlt-1 levels in CD (n=11) and WD (n=14) groups of animals at week 24. (**B**) Plasma sFlt-1 levels in CD (n=19), WD (n=17) and refed (n=12) groups of animals at week 48. (**C**) Relative gene expression levels of VEGFR2 internalization regulators in HDMECs cultured under BSA/Man or Pal/Glu/Ins conditions (n=4). (**D**) Intracellular levels of ROS production in HDMECs cultured under indicated conditions as measured by H_2DFFDA. Scale bar: 20 µm. (**E**) Quantification of H_2DFFDA fluorescence intensity under above-mentioned conditions (n=4). Data are shown as individual points. Statistics are based on unpaired, two-tailed student's *t*-tests (**A**-**C**) or one-way ANOVA (**E**), ****p<0.0001 and ns (*p>0.05).

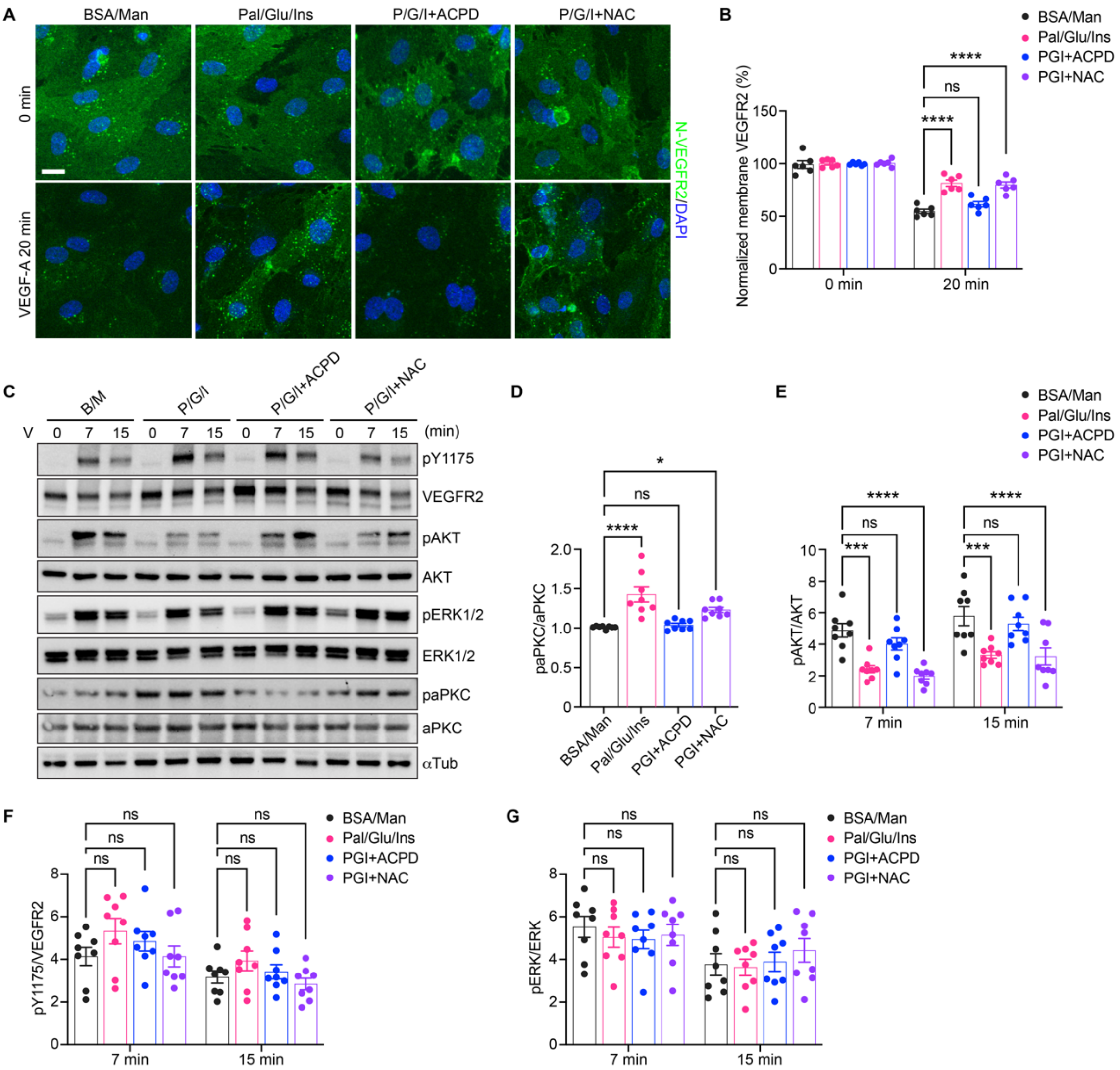

Supplemental Figure 9. ROS accumulation under diet-mimicking condition does not lead to diminished VEGF-A–induced VEGFR2 internalization and AKT phosphorylation

(**A**) Immunofluorescence staining of HDMECs cultured under indicated conditions, showing cell surface VEGFR2 abundance before and 20 minutes after VEGF-A stimulation. Scale bar: 20 μm. (**B**) Quantification of relative fluorescence intensity from cell surface VEGFR2 staining (n=3). (**C**) Western blots showing VEGF-A–induced phosphorylation of downstream signaling molecules in HDMECs cultured under indicated conditions. (**D**) Normalized aPKC phosphorylation at baseline level before stimulation in HDMECs cultured under indicated conditions (n=4). (**E-G**) Normalized AKT (**E**), VEGFR2Y1175 (**F**) and ERK1/2 (**G**) phosphorylation levels during VEGF-A stimulation in HDMECs cultured under indicated conditions (n=4). Statistics are based on two-way ANOVA, *$p<0.05$, ***$p<0.001$, ****$p<0.0001$ and ns (*p>0.05).

Supplemental Table 1. Donor information for human islets.

**Donor number**	**Age (years)**	**Sex**	**Height (cm)**	**Weight (kg)**	**BMI (kg/m^2)**	**HbA1c (mmol/mol)**	**Other comments**
H2668	31	Female	175	73	23.8	36.0	Healthy
H2673	72	Male	179	75	23.4	36.0	Healthy
H2694	54	Male	169	68	23.8	39.0	Healthy
H2709	71	Female	165	60	22.0	36.0	Healthy
H2678	50	Female	155	85	35.4	48.0	Obese/prediabetic
H2681	59	Female	160	90	35.2	37.0	Obese
H2690	44	Male	188	126	35.6	37.0	Obese
H2696	40	Female	190	109	30.2	35.0	Obese
H2711	64	Male	185	110	32.1	46.0	Obese/prediabetic
H2716	62	Male	180	106	32.7	40.0	Obese/prediabetic
H2726	64	Male	170	81	28.0	52.0	Prediabetic
H2689	48	Male	180	87	26.9	47.0	Prediabetic
H2691	54	Female	156	58	23.8	47.0	Prediabetic
H2698	62	Male	185	98	28.6	63.0	T2D

Supplemental Table 2. Sequences of qPCR primers used in this study.

Gene	Sequence
Tbp	L:5'-TGC TGT TGG TGA TTG TTG GT -3'<br>R:5'-CTG GCT TGT GTG GGA AAG AT -3'
Vegfa	L:5'-CAG GCT GCT GTA ACG ATG AA -3'<br>R:5'-GCA TTC ACA TCT GCT GTG CT -3'
Kdr	L:5'-GGC GGT GGT GAC AGT ATC TT -3'<br>R:5'-GTC ACT GAC AGA GGC GAT GA -3'
Pecam1	L:5'-CTG AGC CTA GTG TGG AAG GC -3'<br>R:5'-GTC TCT GTG GCT CTC GTT CC -3'
L19	L:5'-GGC ACA TGG GCA TAG GTA AG -3'<br>R:5'-CCA TGA GAA TCC GCT TGT TT -3'
TBP	L:5'- ACA ACA GCC TGC CAC CTT AC-3'<br>R:5'- GCC ATA AGG CAT CAT TGG AC-3'
VEGFA	L:5'- CCC ACT GAG GAG TCC AAC AT-3'<br>R:5'- AAA TGC TTT CTC CGC TCT GA-3'
KDR	L:5'-AAC GGC GCT TGG ACA GCA -3'<br>R:5'-CAT GCC CTT AGC CAC TTG GAA -3'
PECAM1	L:5'-TCC GGA TCT ATG ACT CAG GG -3'<br>R:5'-ACA GTT GAC CCT CAC GAT CC -3'
EFNB2	L:5'-GTA CCG GAG GAG ACA CAG GA -3'<br>R:5'-CCT TCT CGT AGT GAG GGC AG -3'
PARD3	L:5'-CAG CCT TCC CAC TCT CTG GA -3'<br>R:5'-TTC TCG GGC TTC AGT TTG GC -3'
DAB2	L:5'-CGG GTG TTG ACC AGA TGG ATT -3'<br>R:5'-AAC AGG AGG TGA TGC CGT TT -3'
PRKCI	L:5'- GGG TGA AAG CCT ACT ACC GC -3'<br>R:5'- TTC TCC TGG ACA AGG CAT CC-3'
PRKCZ	L:5'- CCC AAG ATG GAA GGG AGC G-3'<br>R:5'- GAA AGC CTC TTC CAG CTC CA-3'

Supplemental Methods

Animal models and diet intervention

C57BL/6J (#000664), *Ai38* (#014538) and *Cdh5-Cre* mouse strains (#017968) were obtained from the Jackson laboratory (Maine, USA), the latter two strains were both backcrossed into C57BL/6J genetic background. At 6-8 weeks of age, male animals were given control diet (#EF CD88137, Ssniff Spezialdiäten, Germany) when they received islet transplantation into their eyes. Animals were then divided randomly into CD and WD groups, the control group continued to receive control diet, while the WD group was given high sugar/high fat western diet instead (#EF TD88137 mod, 0.21% cholesterol, Ssniff Spezialdiäten, Germany).

Human islets

Human pancreata were procured from DBD (Donation after Brain Death) donors and provided by the Nordic Network for Clinical Islet Transplantation (www.nordicislets.org). Human islets were isolated and distributed by the human islet isolation facility at Uppsala University Hospital. Written informed consent to donate organs for medical and research purposes was obtained from donors or relatives of donors, by the National Board of Health and Welfare (Socialstyrelsen), Sweden. Available information on islet donors is listed in Supplemental Table 1. The islets were lysed directly for RNA extraction upon arrival without further cultivation.

Mouse islet isolation and transplantation

Mouse islets were isolated by cannulation of the common bile duct of donor animals and infusion of 3 ml collagenase P, 0.8 mg/ml in HBSS containing 25 mM HEPES and 0.5% BSA (Sigma-Aldrich, USA). Inflated pancreata were carefully dissected out and digested in 37°C water bath for 10 minutes, and individual islets were hand-picked and further cultured in RPMI-1640 Medium (11mM D-glucose) supplemented with 10% fetal bovine serum, 100 IU/ml penicillin, 100 µg/ml streptomycin and 2 mM L-Glutamine (all from Thermo Fisher Scientific, USA).

For transplantation, recipient mice were anesthetized with 2% isoflurane (Baxter, USA) and fixed with a custom-made head and eye holder (Narishige, Japan) as previously described (1). A small

incision was made in the cornea with a 25 G needle and the tip of a glass cannula containing islets was inserted through the opening into the anterior chamber of the eye. 4-5 islets (150-200 μm in diameter) were carefully positioned on the iris around the pupil. Post-operative analgesia was done by subcutaneous injection of 0.1 ml/kg Temgesic (RB Pharmaceuticals Limited, UK). For $[Ca^{2+}]_i$ imaging experiments, 3-4-month-old male C57BL/6J mice were used as islet donors, and islets were kept in culture for a week before transplantation. 6-8-week-old male EC-GC3 mice were used as recipients. For the other *in vivo* imaging experiments, 3-4-month-old and 6-8-week-old male C57BL/6J mice were used as islet donors and recipients respectively, and islets were transplanted after overnight culture.

Glucose tolerance test

Animals were fasted for 6 hours prior to the tests. For intraperitoneal glucose tolerance tests, an amount of 2 g/kg body weight D-glucose (20% solution in PBS+/+, Merck, USA) was injected. Blood was taken from tail veins and blood glucose levels were measured at specified time points using an Accu-Chek Aviva system (Roche, Switzerland). For intravenous glucose tolerance tests, animals were anesthetized with a mixture of 0.5 mg/kg fentanyl, 16 mg/kg fluanisone (Hypnorm, Roche, Switzerland) and 8 mg/kg midazolam (Hameln, Sweden) after fasting. They were then placed on a heating pad (37 °C) under a heating lamp, and oxygen was supplied during the experiments through nose masks at 250 ml/min. 0.5 g/kg D-glucose (10% solution in PBS+/+) was injected through the tail vein, and blood was collected for the measurement of both glucose (Accu-Chek) and insulin levels (Insulin AlphaLISA detection kit, Revvity, USA).

Insulin tolerance test

For intraperitoneal insulin tolerance tests, animals were fasted for 6 hours, 0.25 U/kg insulin (Actrapid, diluted in saline, Novo Nordisk, Denmark) was injected. For intravenous insulin tolerance tests, animals were anesthetized with a mixture of 0.5 mg/kg fentanyl, 16 mg/kg fluanisone (Hypnorm, Roche, Switzerland) and 8 mg/kg midazolam (Hameln, Sweden) without fasting. They were oxygenated and kept warm in the same way as mentioned above, and 0.5 U/kg insulin was injected. Blood was taken

from tail veins and blood glucose levels were measured at specified time points using an Accu-Chek Aviva system (Roche, Switzerland).

In vivo imaging of islet grafts

Imaging was performed at specified time points post-transplantation by confocal microscopy. Animals were anesthetized with either 2% isoflurane inhalation (Baxter, USA) for visualization of islet grafts, or a mixture of 0.5 mg/kg fentanyl, 16 mg/kg fluanisone (Hypnorm, Roche, Switzerland) and 8 mg/kg midazolam (Hameln, Sweden) for measurement of islet vessel physiology. During imaging sessions, animals were kept on a heating pad (37 °C) with a head holder in the heated microscope chamber, and oxygen was supplied throughout the experiments at 250 ml/min. Images of islet grafts were obtained with Leica DM6000 CFS/TCS SP5 confocal microscope equipped with a 25× objective (N.A. 0.95, Leica Microsystems, Germany). Pre-warmed viscotears (Laboratoires Théa, France) was used as an immersion medium between the lens and the mouse eyes. Morphology of islet graft was visualized by obtaining backscatter signal in all the imaging sessions (Ex.: 633 nm, Em.:630-636 nm).

For the visualization of islet vasculature, 100 µl of 2.5 mg/ml of 40-kDa FITC-conjugated dextran (Thermo Fisher Scientific, USA) in PBS+/+ was injected intravenously prior to imaging. Z-stacks of 2 µm thickness per step were acquired for every islet graft (Ex.: 488 & 633 nm, Em.: 500-540 nm & 630-636 nm).

For $[Ca^{2+}]_i$ imaging, a tail vein catheter containing 100 IE/ml heparin and 10 µg/ml mouse VEGF-A in sterile saline was inserted after anesthesia and prior to imaging. 10 µl heparin was directly injected, while 100 µl/30 g VEGF-A was injected after 1 minute of baseline signal collection. Z stacks of 140 µm thickness in total (4 µm per step) were acquired with resonance scanner for every islet graft (Ex.: 488 & 633 nm, Em.: 500-550 nm & 630-636 nm).

For quantification of vascular leakage, a tail vein catheter containing 100 IE/ml heparin and 10 mg/ml of 3-5 kDa FITC-conjugated dextran in PBS+/+ was inserted after anesthesia and prior to imaging. 10 µl heparin was directly injected, while 100 µl/ 30 g of FITC-dextran was injected 1-2

minutes later. Z stacks of 40 μm thickness in total (2 μm per step) were acquired with resonance scanner for every islet graft (Ex.: 488 & 633nm, Em.: 500-540 nm & 630-636 nm).

For monitoring blood flow dynamics, RBCs were labelled as previously described (2). Briefly, 35 μl blood was extracted from the tail vein, and 70 μl binding buffer (128 mM NaCl, 15 mM glucose, 10 mM HEPES, 4.2 mM $NaHCO_3$, 3 mM KCl, 2 mM $MgCl_2$, and 1 mM KH_2PO_4, pH adjusted to 7.4) was added. RBCs were purified by centrifugation at 1500 rpm (220 x g) for 5 minutes and resuspended in 140 μl binding buffer, and further labelled with 3.5 μl carbocyanide 1,1′-dioctadecyl-3,3,3′,3′-tetramethylindodicarbocyanine, 4-chlorobenzenesulfonate salt (DiD solid, Thermo Fisher Scientific, USA) for 10 minutes at 37 °C. The fluorescent RBCs were then washed with binding buffer three times before intravenous injection. Afterwards, a tail vein catheter containing 100 IE/ml heparin and 10 μg/ml mouse VEGF-A in sterile saline was inserted after anesthesia onset and prior to imaging. 10 μl heparin was directly injected, while 100 μl/30 g VEGF-A was injected after 1 minute of baseline flow recording. Fast *xyt* imaging was performed with resonance scanner for every islet graft (Ex.: 561 & 633 nm, Em.: 558-564 nm & 650-750 nm). Around 20 RBCs were randomly picked at each time point for each islet, and individual RBC flow velocity was calculated by comparing two consecutive images.

Image analysis

Unprocessed original images were used in the quantification of islet morphology and blood flow rate. Graft volume was estimated from backscatter signals, and dextran-labelled islet capillary structures were used for the estimation of vascular volume and diameters (Volocity, Perkin Elmer, USA), with same thresholds used for all groups at all time points. Relative islet volume of each graft in percentage was calculated from the values measured at individual time points and divided by the value at week 0. Relative vascular volume in percentage was calculated by dividing the volume of islet vessels by the estimated islet volume at each time point.

Time-lapse confocal image stacks were analyzed, and fluorescence intensity was quantified using Fiji (3) with the plugin Time Series Analyzer V3. For $[Ca^{2+}]_i$ imaging, movements were corrected with a custom MATLAB program (Mathworks, USA), and all visible vessel segments were hand-picked for

each islet. Dextran leakage measurement was performed as described before (4). Islet borders were identified by backscatter signals, and average fluorescence intensity was measured in a ring structure of 30 pixels thickness outside each islet.

VEGF-A and sFlt-1 measurement

For measuring ex vivo VEGF-A production, isolated islets were cultured overnight. 30 islets from each group were picked into 4-well plates containing 450 µl culture medium per well. 72 hours later, supernatants were collected, briefly centrifuged and stored at -80 ºC before measurement (Mouse VEGF Quantikine ELISA Kit, R&D Systems, USA). Islets were collected and lysed in 50 µl T-PER buffer (Thermo Fisher Scientific, USA) to quantify the DNA contents for normalization (Quant-iT PicoGreen dsDNA Assay Kit, Thermo Fisher Scientific, USA). 15 µl blood was taken from the tail vein from non-fasted animals around 9 A.M. for the measurement of plasma sFlt-1(Mouse VEGFR1/Flt-1 Quantikine ELISA Kit, R&D Systems, USA) levels. Secreted VEGF-A in supernatants, plasma sFlt-1and islet DNA contents were all measured according to manufacturers' instructions.

VEGFR-2 internalization

1×10^5 HDMECs (PromoCell, Germany) were plated on coverslips in 6 well plates and treated with 0.075% BSA and 25 mM Mannitol (BSA/Man) in the control group, or 100 µM palmitate (conjugated to BSA at a ratio of 4:1) , 25 mM D-glucose and 1 µM human insulin (Pal/Glu/Ins) in the diet-mimicking group for 6 days. The recovery group was treated with Pal/Glu/Ins for 3 days and BSA/Man for another 3 days. In some experiments, 10 mM ACPD (Merck, USA) or 0.5 mM NAC (Merck, USA) was used to pretreat the HDMECs during cultivation under Pal/Glu/Ins condition. On day 7, confluent cells were starved for 6 hours with MV2 (PromoCell, Germany) supplemented with 0.5% FBS. Starved cells were gently washed twice with PBS, and half of the cells were stimulated with 50 ng/ml human VEGF-A (Peprotech, Sweden) for 20 minutes at 37ºC. VEGF-A containing medium was quickly removed afterwards and cells were washed twice in ice-cold PBS. Subsequently, cells were fixed in 4% paraformaldehyde solution at room temperature for 15 minutes and blocked with 1% BSA in PBS for

1 hour without permeabilization. Further incubations with anti-VEGFR2 N-terminal antibody (rabbit, 1:100, #AF357, R&D Systems, USA) and goat anti-rabbit IgG H+L Alexa Fluor 488 (1:1000, #A-11008, Thermo Fisher Scientific, USA) were carried out at room temperature for 1 hour each, and cells were washed 3 times with PBS+/+ in between.

Lentiviral production and endothelial cell transduction

We used a commercial lentiviral system from Merck for designing and construct shRNA containing lentiviral vectors. Two verified sequences per gene of interest (Homo sapiens) were selected from the database (https://www.sigmaaldrich.com/SE/en/semi-configurators/shrna?activeLink=productSearch). For PKCi: 5'-CCAGTCTAGGTCTTCAGGATT-3' (shPRKC1-1) and 5'-CTTCATGAGCGAGGG ATAATT-3' (shPRKC1-2). For PKCζ: 5'-CTGGTGCGGTTGAAGAAGAAT-3' (shPRKCZ-1) and 5'-GCCTCCAGTAGACGACAAGAA-3' (shPRKCZ-2). Pre-made pLKO.1-puro vectors containing the corresponding oligonucleotides were ordered and used for lentivirus production (Merck, USA). Lentivirus was produced in 293FT cells (Thermo Fisher Scientific, USA) and harvested following the manufacturer's guidelines (https://www.sigmaaldrich.com/life-science/functional-genomics-and-rnai/shrna/trc-shrnaproducts/lentiviral-packaging-mix.html). 2×10^5 HDMEC cells were plated in 25 cm^2 flasks and 1 ml of crude virus containing medium was used for transduction. Transduced cells were further screened in 1.0 µg/ml puromycin (Merck, USA) for a week before use, and the knockdown efficacy was assessed by western blots. Taking into consideration of both knockdown efficiency and viability of silenced cells, we used shPRKC1-1 and shPRKCZ-2 in the assays for VEGF-A signal transduction.

ROS measurements

1×10^5 HDMECs were plated on coverslips in 35 mm culture dishes and incubated with BSA/Man or Pal/Glu/Ins for 6 days as previously described. 0.5 mM NAC (Merck, USA) was used to eliminate ROS production in HDMECs cultured under Pal/Glu/Ins condition. On the day of imaging, cells were washed once with D-PBS, loaded with 40 µM Carboxy-H_2DFFDA (Thermo Fisher Scientific, USA) for 60

minutes, washed twice with D-PBS and then allowed equilibration for another 10 minutes before imaging. Excitation at 488 nm and emission at 500-540 nm was used to detected H_2DFFDA fluorescence.

Pancreatic sections and immunofluorescence staining

For staining of pERK1/2, animals were anesthetized with a mixture of 0.5 mg/kg fentanyl, 16 mg/kg fluanisone (Hypnorm, Roche, Switzerland) and 8 mg/kg midazolam (Hameln, Sweden), and were kept on a heating pad (37 °C) with oxygen supply throughout the experiment at 250 ml/min for 10 minutes. Afterwards, 100 µl/30 g mouse VEGF-A at 10 µg/ml was injected through the tail vein and animals were kept on the heating pad for another 5 minutes before being sacrificed quickly by cervical dislocation. Pancreata were swiftly removed from euthanized animals and rinsed in cold PBS+/+, which took an additional minute before fixation by 4% paraformaldehyde in PBS+/+ at room temperature for 2 hours. Tissues were then washed with PBS+/+ twice and cryopreserved stepwise in 10%, 20% and eventually 30% sucrose in PBS+/+ at 4 ºC overnight. They were embedded in O.C.T. freezing medium (Thermo Fisher Scientific, USA) at -80 ºC and subsequently cut into 20 µm thick sections for staining. Sections or fixed cells were incubated with primary antibodies, including anti-Phospho-PKCζ/λ (Thr410/403) antibody (rabbit, 1:100, #9378, Cell Signaling Technology, USA), anti-VEGF-A antibody (goat, 1:50, #AF-493-NA, R&D Systems, USA), anti-insulin antibody (rabbit, 1:500, #ab282459, Abcam, UK), anti-PECAM-1 antibody (goat, 1:400, #AF3628, R&D Systems, USA), anti-VEGFR2 (rabbit, 1:200, #9698, Cell Signaling Technology, USA), and phospho-p44/42 MAPK Thr202/Tyr204 antibody (rabbit, 1:200, #4370, Cell Signaling Technology, USA), at 4 ºC overnight. After washing with PBS+/+ 3 times, samples were further incubated with secondary antibodies (donkey anti-rabbit IgG H+L Alexa Fluor 546 (#A10040) and donkey anti-goat IgG H+L Alexa Fluor 488 (#A-11055, both at 1:1000, Thermo Fisher Scientific, USA) at 4 ºC overnight and mounted with fluorescence mounting medium (Agilent, USA).

Western Blots

Confluent HDMECs were stimulated by 50 ng/ml human VEGF-A for indicated lengths of time and lysed in M-PER buffer (Thermo Fisher Scientific, USA) supplemented with protease and phosphatase inhibitors (Mini Complete/PhosphoStop, Roche Diagnostics, Germany). Protein contents were determined using the BCA Protein Assay Kit (Pierce, USA). Protein extracts were separated on 4-15% stain-free precast gels (Bio-Rad, USA) and transferred to 0.22 μm PVDF membranes (GE Healthcare, USA). Blots were probed with primary antibodies, including phospho-VEGFR2Y1175 antibody (rabbit, 1:1000, #2478), VEGFR2 antibody (rabbit, 1:1000, #2479), Phospho-PKCζ/λ (Thr410/403) antibody (rabbit, 1:1000, #9378), PKCι/λ antibody (rabbit, 1:1000, #2998), PKCζ antibody (rabbit, 1:1000, #9368), phospho-Akt Thr308/473 antibody (rabbit, 1:1000, #4060), Akt1/2 antibody (rabbit, 1:1000, #4691), phospho-p44/42 MAPK Thr202/Tyr204 antibody (rabbit, 1:2000, #4370), Erk1/2 antibody (rabbit, 1:2000, #4695; all from Cell Signaling Technology, USA), and α-tubulin antibody (mouse,1:1000, #T6199, Merck, USA), and subsequently probed with secondary anti-mouse/-rabbit IgG (H+L)-HRP conjugates (1:6000, #NA931 and #NA934, GE Healthcare, USA). Quantification of phosphorylated protein was carried out on original scanned images using Image Lab software (Bio-Rad, USA) and normalization to respective total protein amount.

In vitro perifusion of islets

Freshly isolated islets were kept in culture for 2 hours, and 70-80 islets from each group were put in an automated perifusion system (BioRep Perifusion System, BioRep, USA) for the evaluation of dynamic insulin release *in vitro*, as described before (5). Briefly, islets were immobilized in a gel (Bio-Gel P-4, Bio-Rad, USA) in flow chambers, and were perifused with specified solutions at a speed of 50 μl/min at 37 ºC for indicated periods. Perfusates were continuously and automatically collected into 96-well plates for the detection of insulin (Insulin AlphaLISA Detection Kit, Revvity, USA). Insulin measurement was performed according to the manufacturer's instructions.

Transmission electron microscopy (TEM)

Islet grafts were dissected out from the eyes of euthanized recipient animals, and peri-islet iris tissues were removed carefully with micro scissors. Pancreata were swiftly removed from euthanized animals and cut into around 1 mm^3 pieces. Tissues were then fixed in 2.5% glutaraldehyde and 1% paraformaldehyde in 0.1 M phosphate buffer (pH 7.4) at 4 °C overnight. Samples were further washed in 0.1 M phosphate buffer (pH 7.4) and post-fixed in 2% osmium tetroxide 0.1 M phosphate buffer (pH 7.4) at 4 °C for 2 hours, dehydrated in ethanol and acetone, and embedded in LX-112 (Ladd Research Industries, Burlington, USA). Ultrathin sections of 50–60 nm thickness were made by Leica EM UC6 ultramicrotome (Leica Microsystems, Germany) and contrasted with uranyl acetate, followed by lead citrate treatment and examined in Tecnai 12 Spirit BioTWIN transmission electron microscope (FEI company, USA) at 100 kV. Images were acquired by 2kx2k Veleta OSiS CCD camera (Olympus Soft Imaging Solutions, Germany).

Gene expression analysis

Total RNA samples were extracted from freshly harvested mouse islets, human islets and HDMECs with GeneJET RNA purification kit (Thermo Fisher Scientific, USA), and extracted mRNA was transcribed into cDNA using the Maxima First Strand cDNA Synthesis Kit (Thermo Fisher Scientific, USA), all according to manufacturer's instructions. Mouse and human gene transcripts were measured using PowerUp SYBR Green Master Mix (Thermo Fisher Scientific, USA) in QuantStudio™ 5 Real-Time PCR System (Applied Biosystems, USA). Relative gene expression levels were quantified against the housekeep genes *Tbp, TBP* or *L19* messages and normalized to those of control islets or HDMECs respectively. All primer sequences are listed in Supplemental Table 2. Single cell RNA sequencing data from the Human Pancreas Analysis Program (HPAP) (6) was used for human islet β cell and endothelial cell gene expression. Gene expression plots were generated using CELLxGENE (7).

References

1. Speier S, et al. Noninvasive high-resolution in vivo imaging of cell biology in the anterior chamber of the mouse eye. *Nat Protoc.* 2008;3(8):1278-86.
2. Kornfield TE, and Newman EA. Regulation of blood flow in the retinal trilaminar vascular network. *J Neurosci.* 2014;34(34):11504-13.
3. Schindelin J, et al. Fiji: an open-source platform for biological-image analysis. *Nat Methods.* 2012;9(7):676-82.
4. Xiong Y, et al. Islet vascularization is regulated by primary endothelial cilia via VEGF-A-dependent signaling. *Elife.* 2020;9.
5. Molina J, et al. Control of insulin secretion by cholinergic signaling in the human pancreatic islet. *Diabetes.* 2014;63(8):2714-26.
6. Shapira SN, et al. Understanding islet dysfunction in type 2 diabetes through multidimensional pancreatic phenotyping: The Human Pancreas Analysis Program. *Cell Metab.* 2022;34(12):1906-13.
7. Megill C, et al. Cellxgene: a performant, scalable exploration platform for high dimensional sparse matrices [preprint]. https://doi.org/10.1101/2021.04.05.438318. Posted on bioRxiv April 5, 2021.

Supplemental Video 1. Representative movie showing intra-islet vessel Ca^{2+} response to VEGF-A bolus in CD-fed animals at week 12. Time series of confocal images are presented as maximum intensity projections. The movie is accelerated, and real time duration is 8 min. VEGF-A was injected at 1 min as indicated. Scale bar: 50 μm.

Supplemental Video 2. Representative movie showing intra-islet vessel Ca^{2+} response to VEGF-A bolus in WD-fed animals at week 12. Time series of confocal images are presented as maximum intensity projections. The movie is accelerated, and real time duration is 8 min. VEGF-A was injected at 1 min as indicated. Scale bar: 50 μm.

Supplemental Video 3. Representative movie showing the labeling of RBCs for in vivo quantification of islet blood flow velocity in a control animal. Scale bar: 50 μm.
