# Supplementary figures and images for: Diet-induced obesity promotes endothelial cell desensitization to VEGF-A and permanent islet vessel dysfunction in mice
Source: J Clin Invest. 2025 Jun 5;135(15):e177601. doi: 10.1172/JCI177601 (PMC12321391; doi:10.1172/JCI177601)

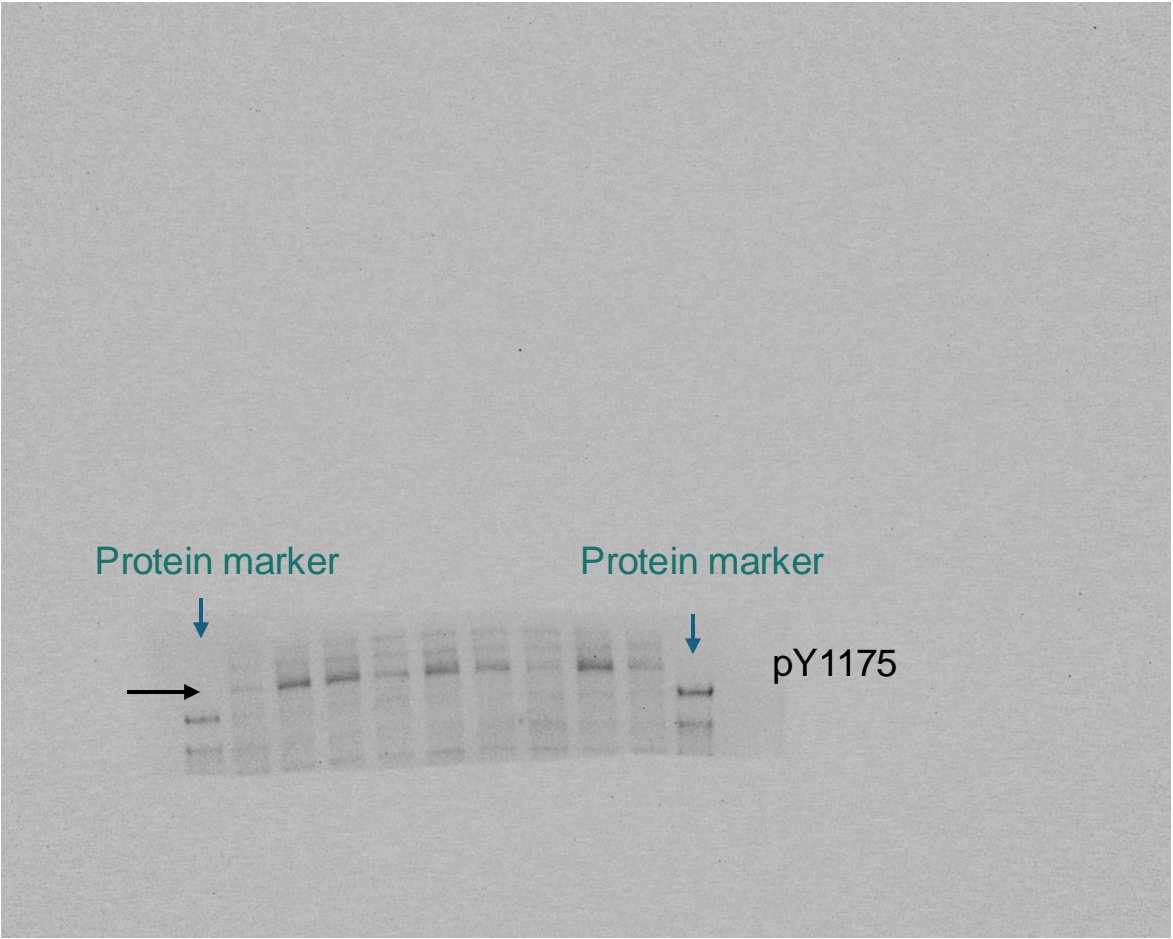

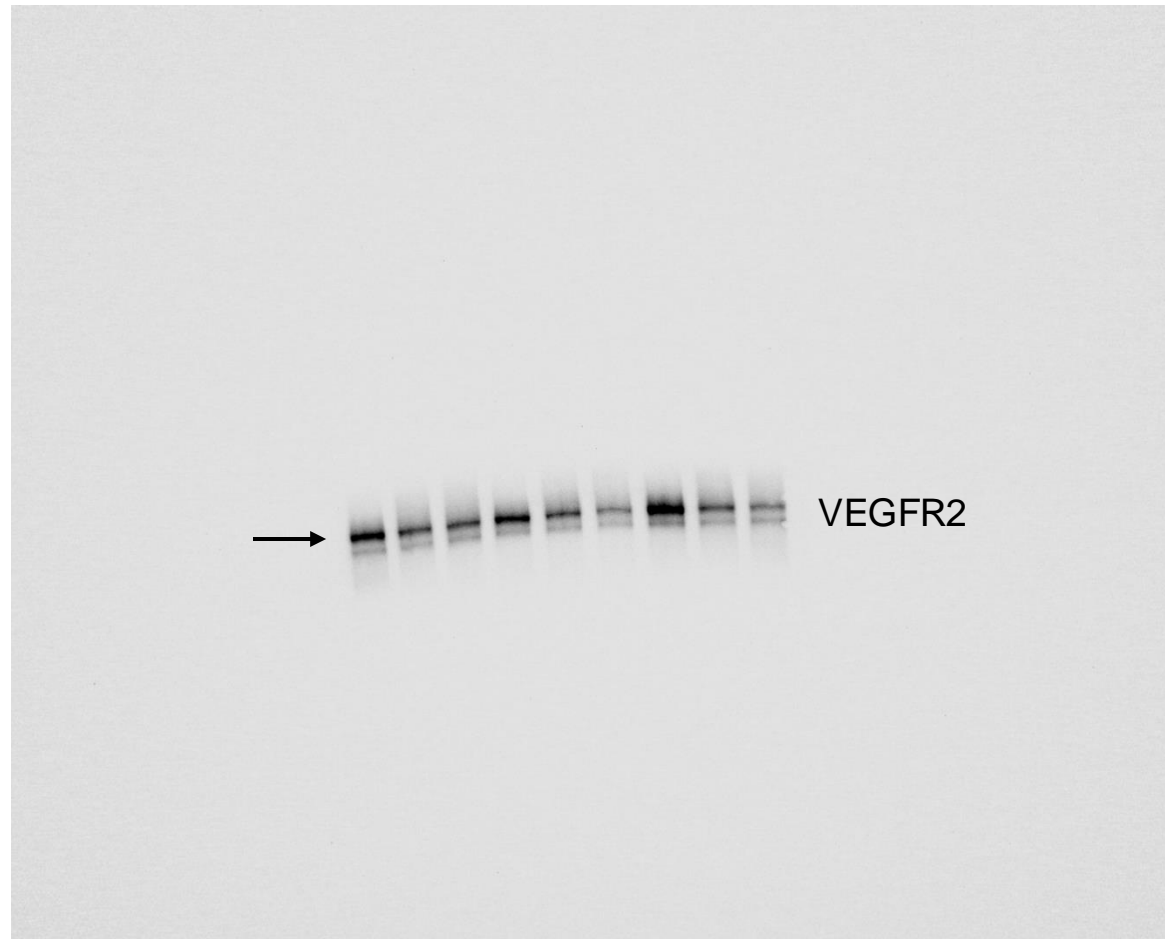

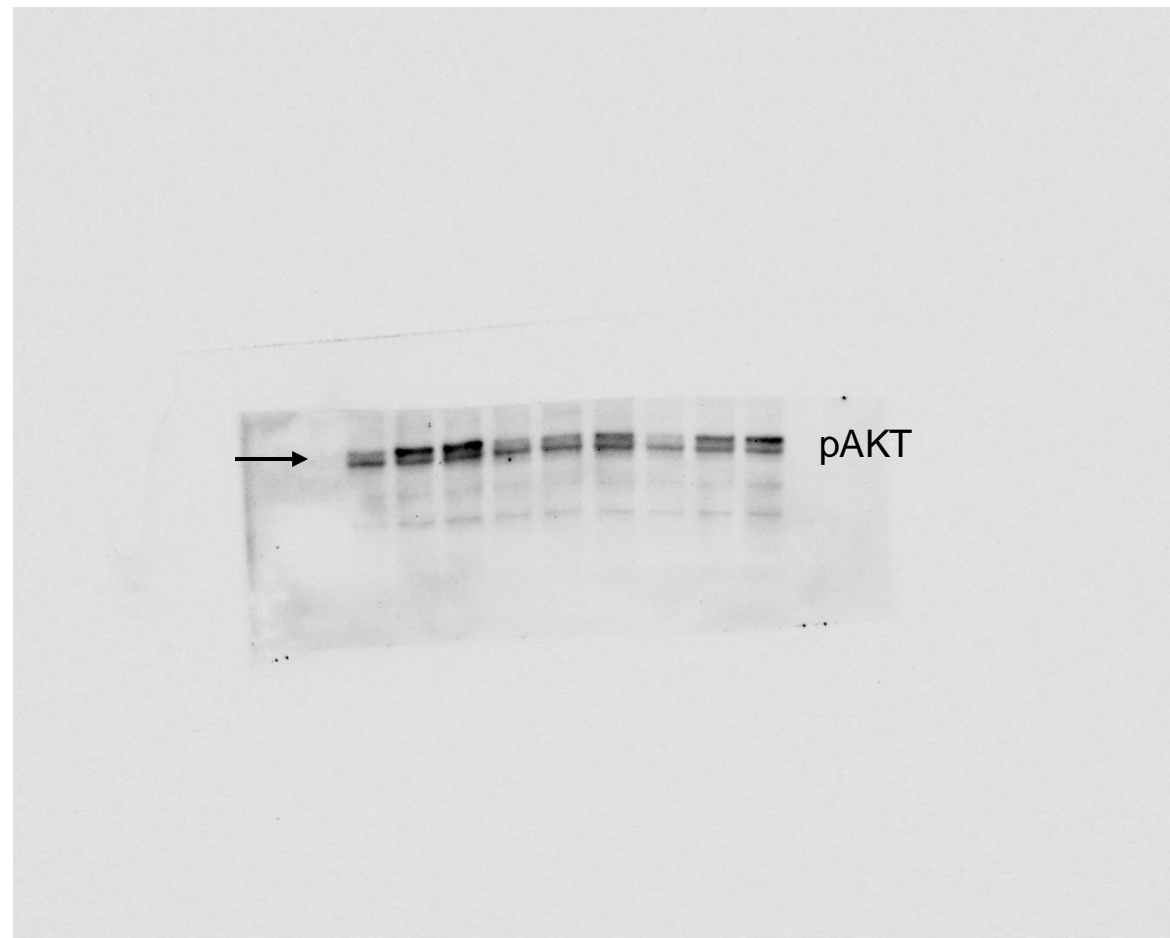

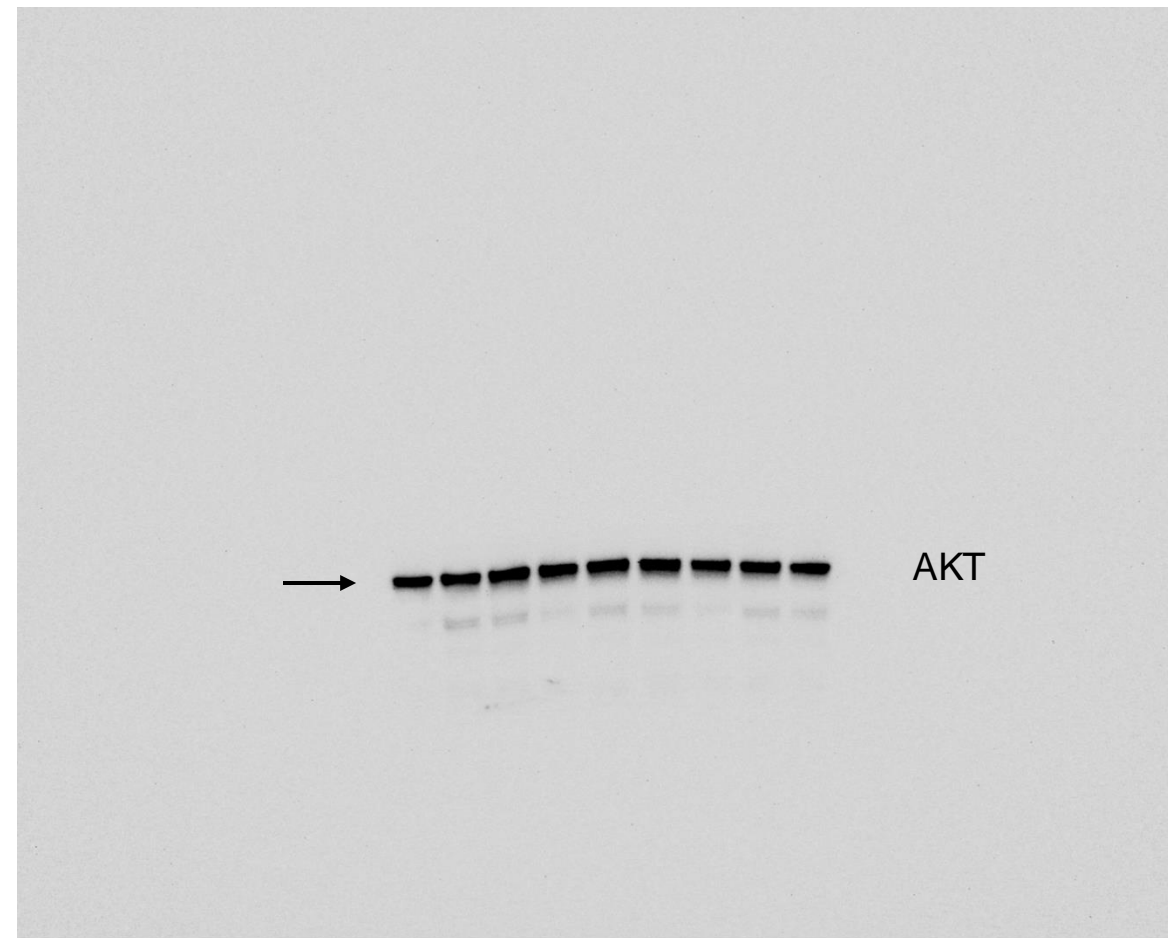

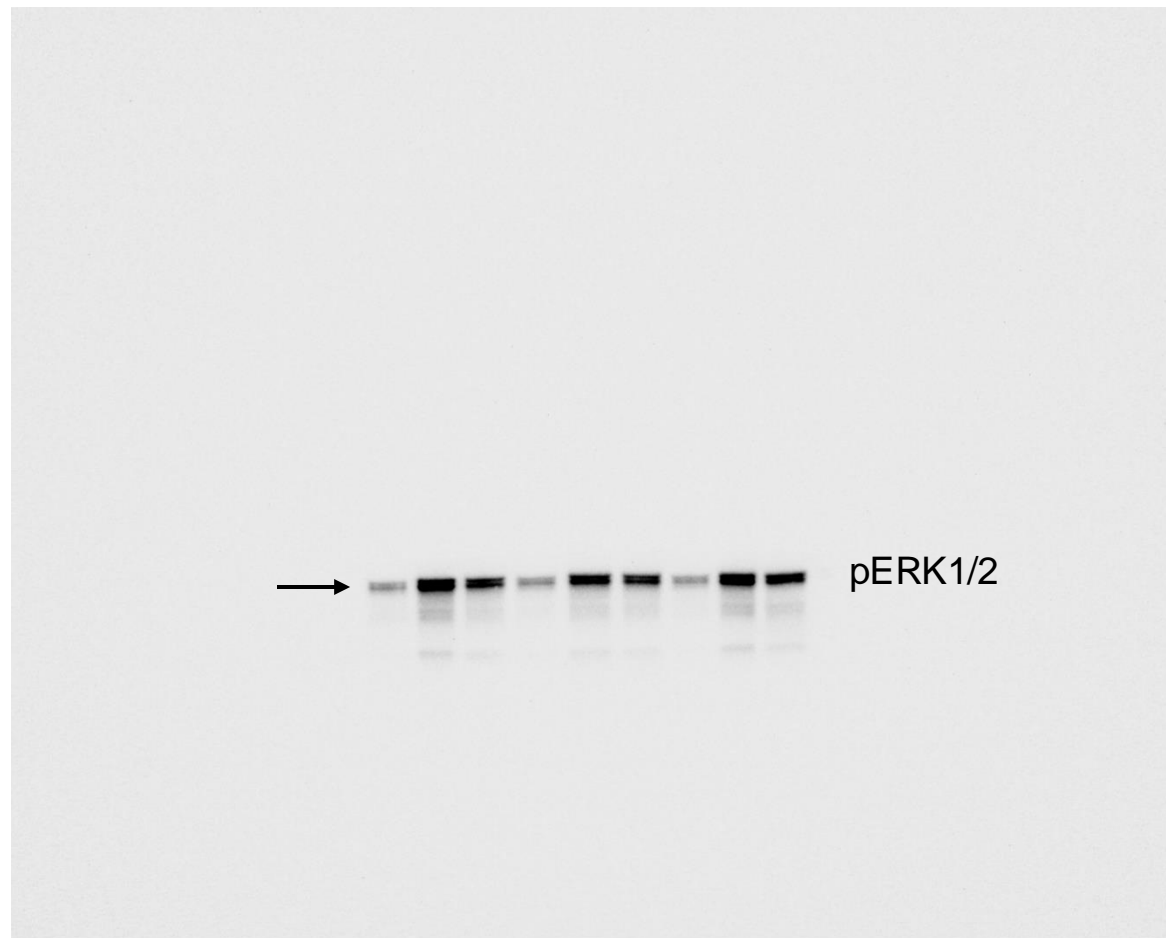

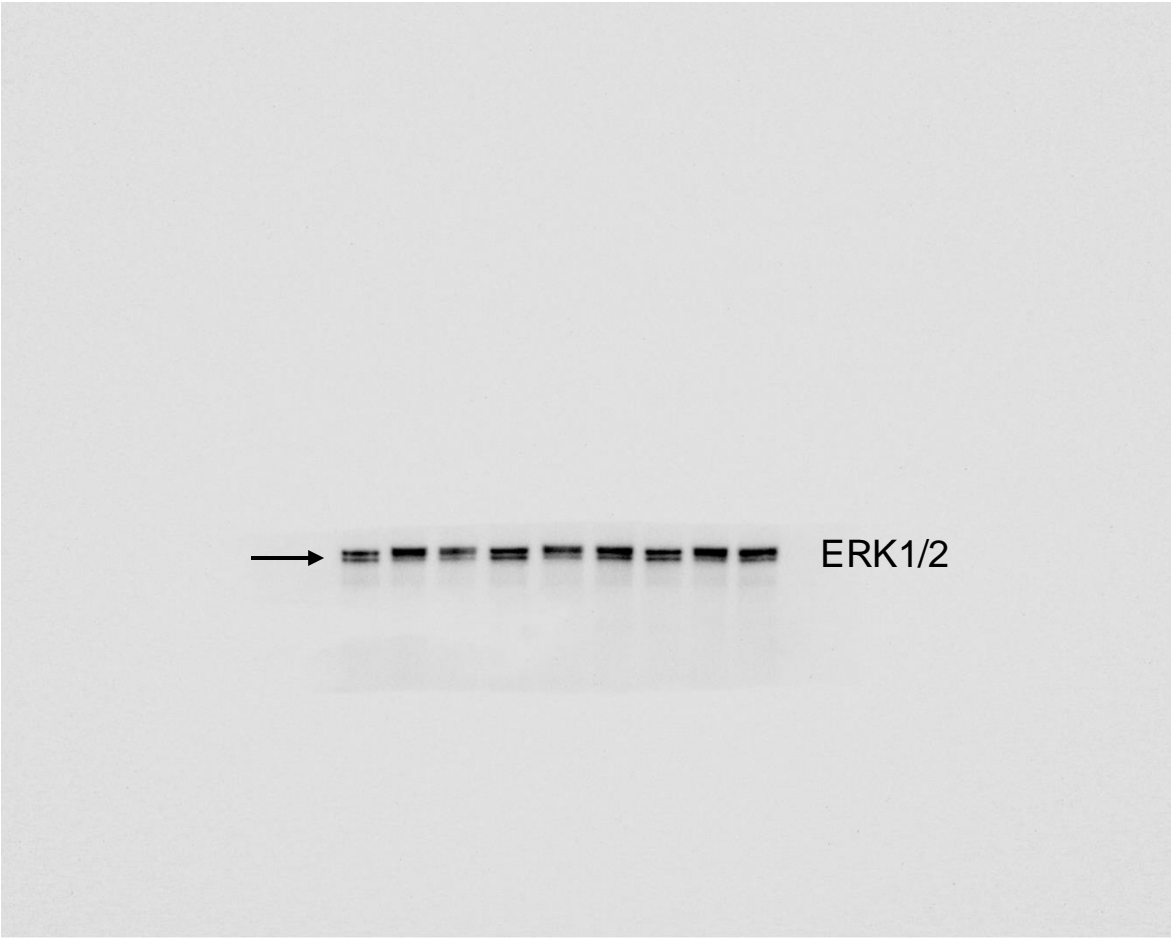

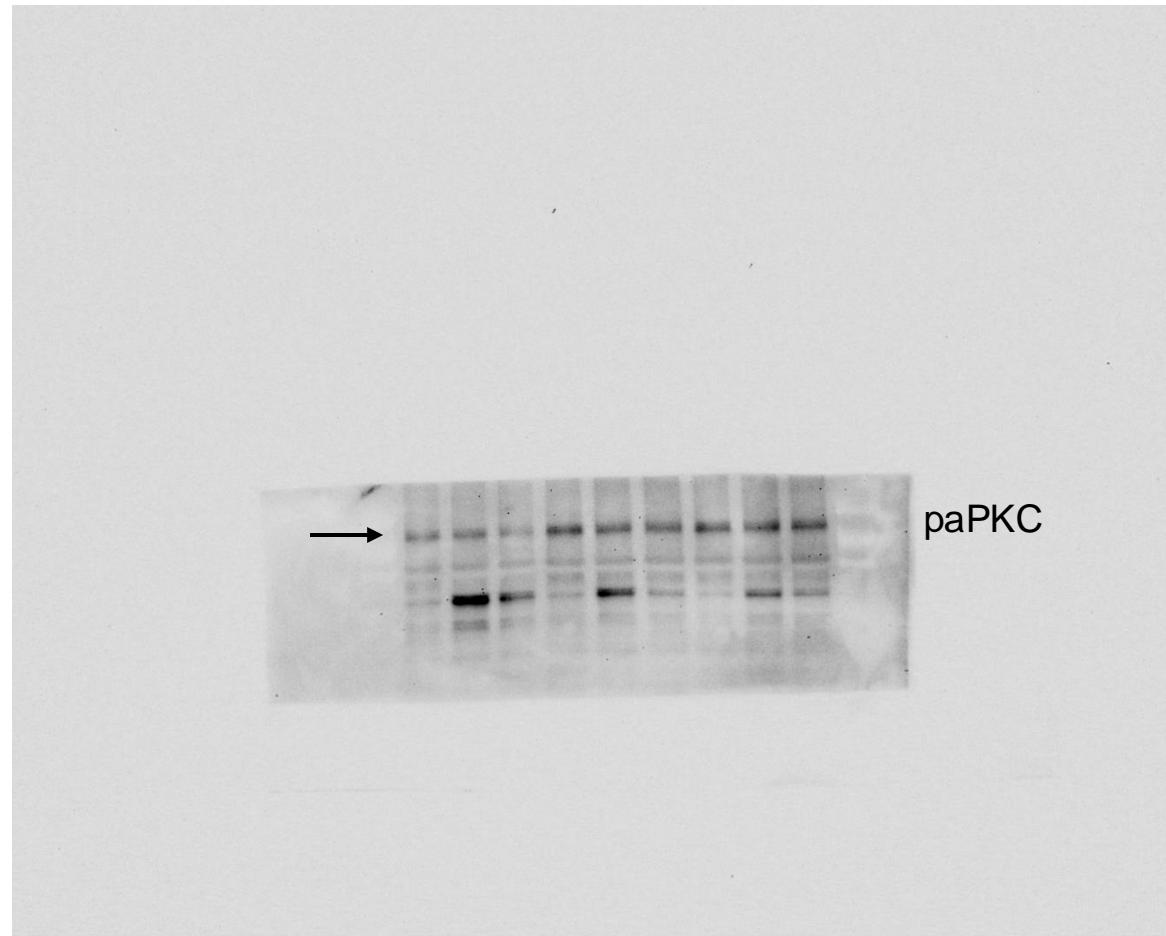

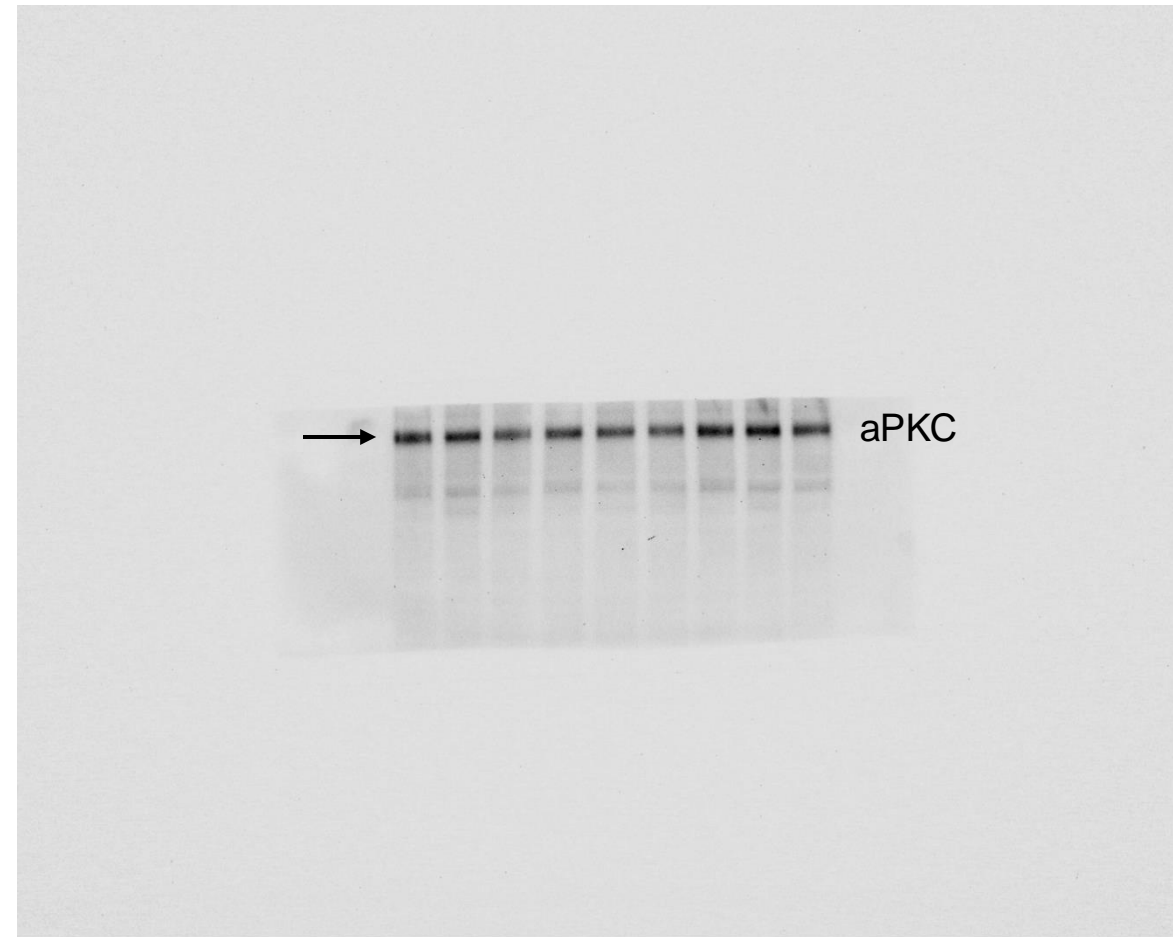

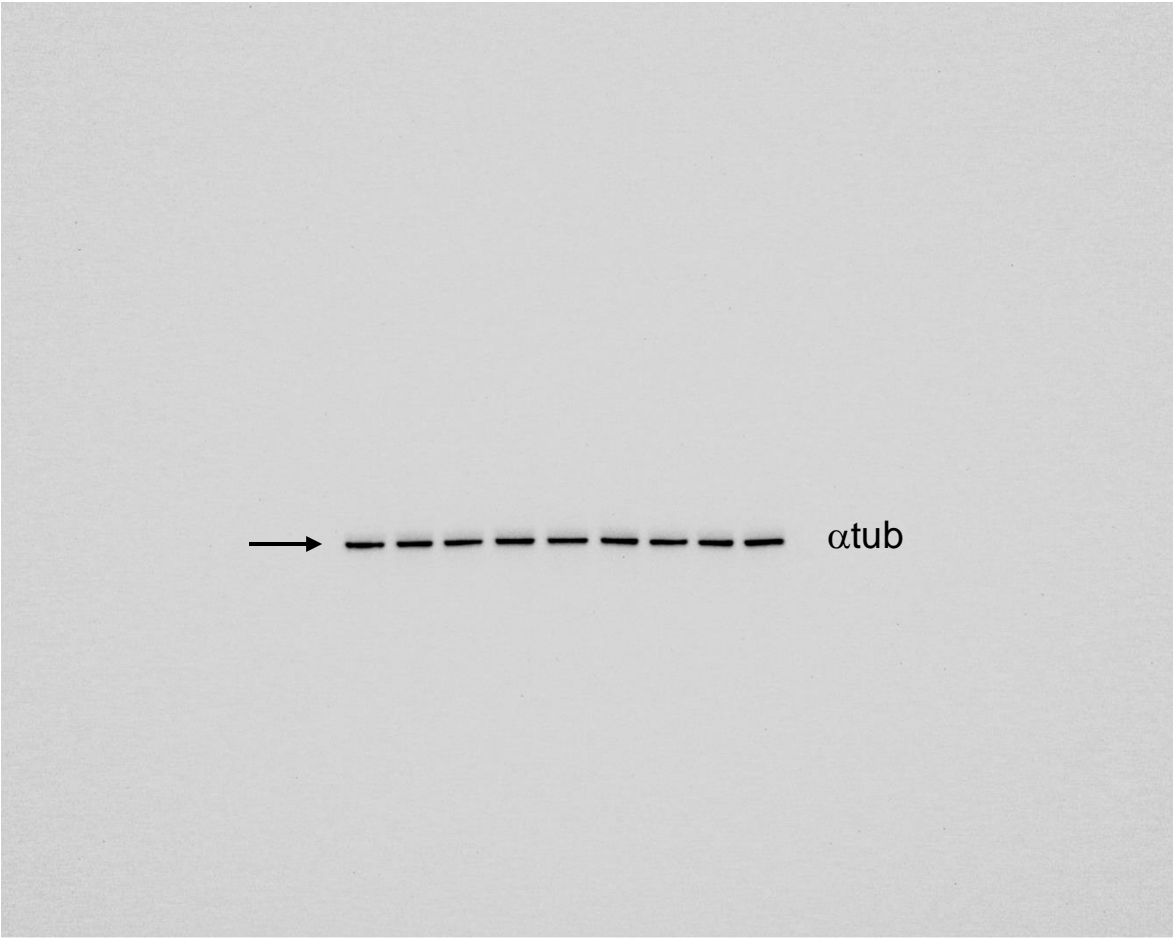

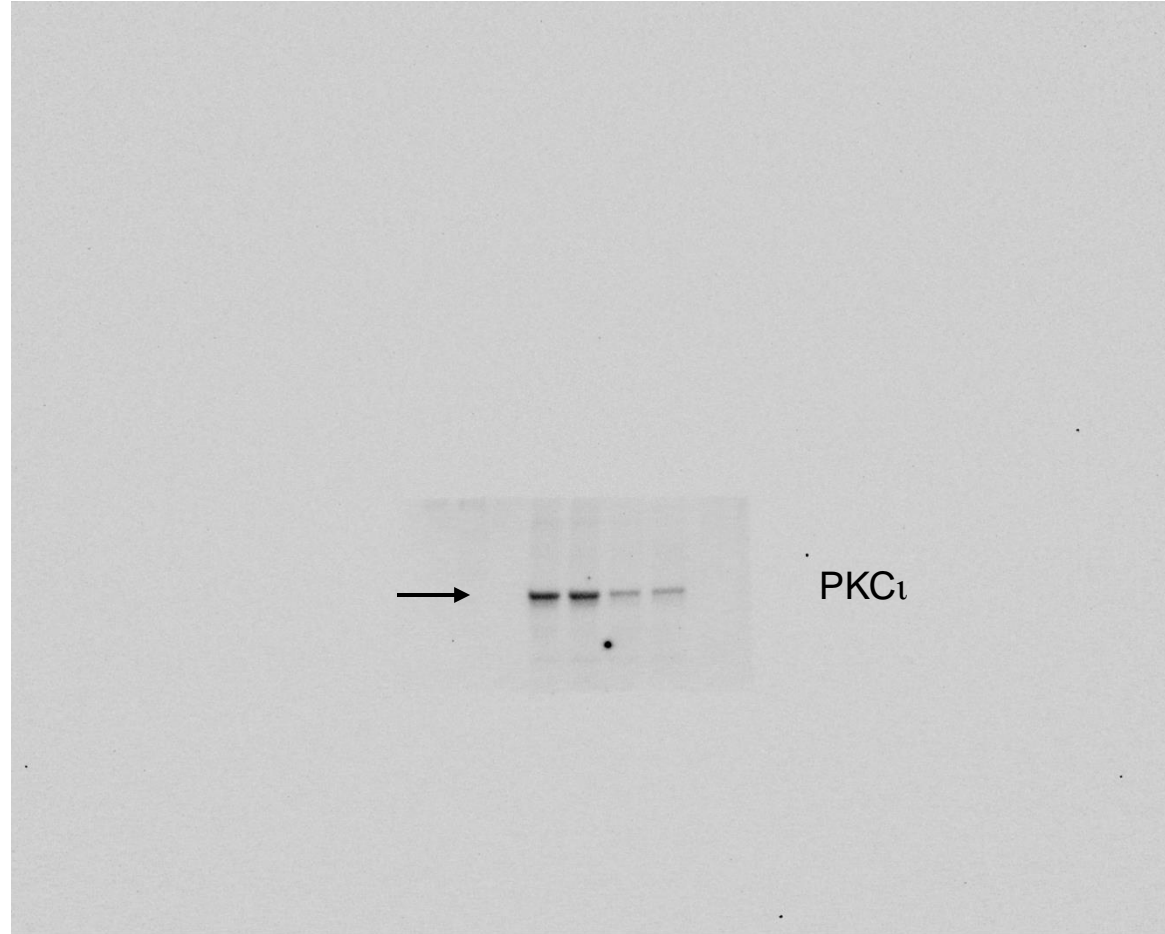

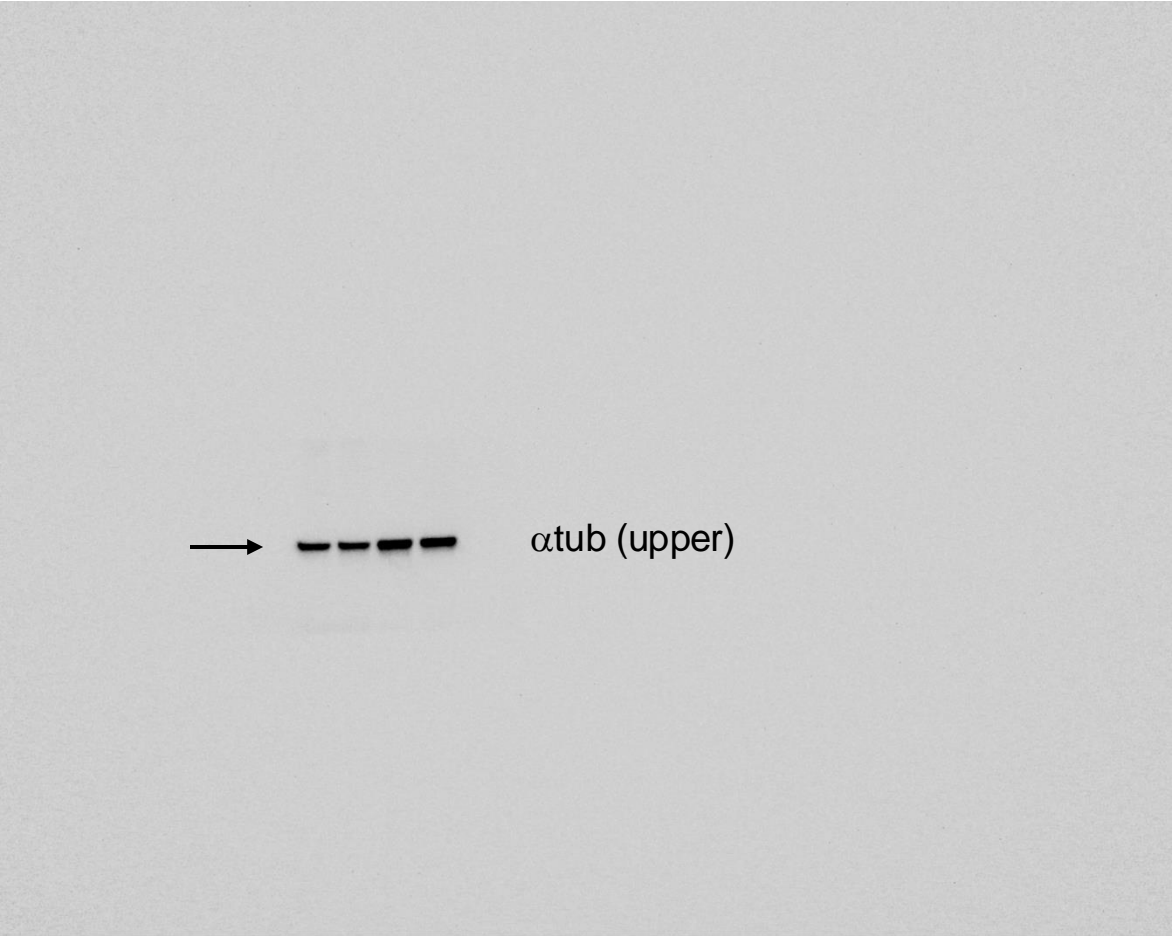

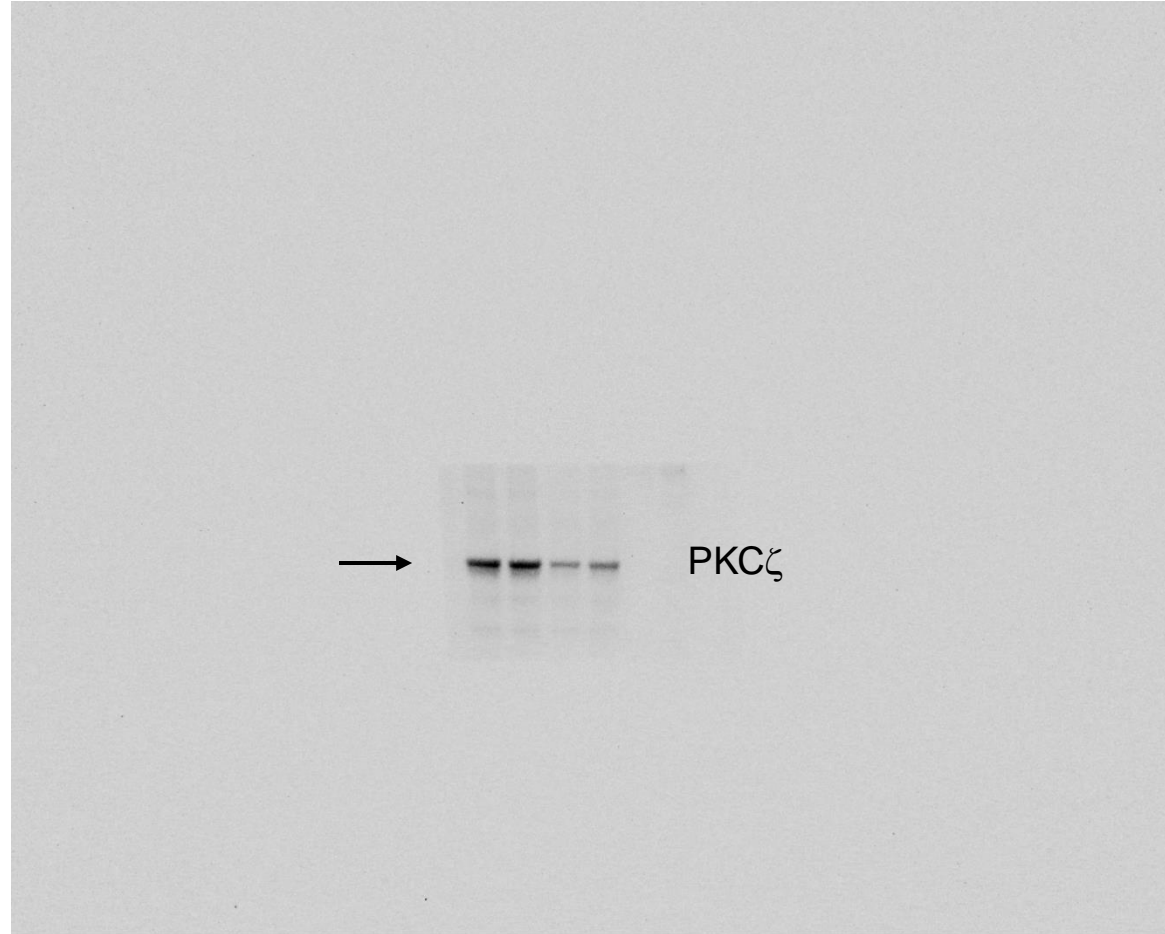

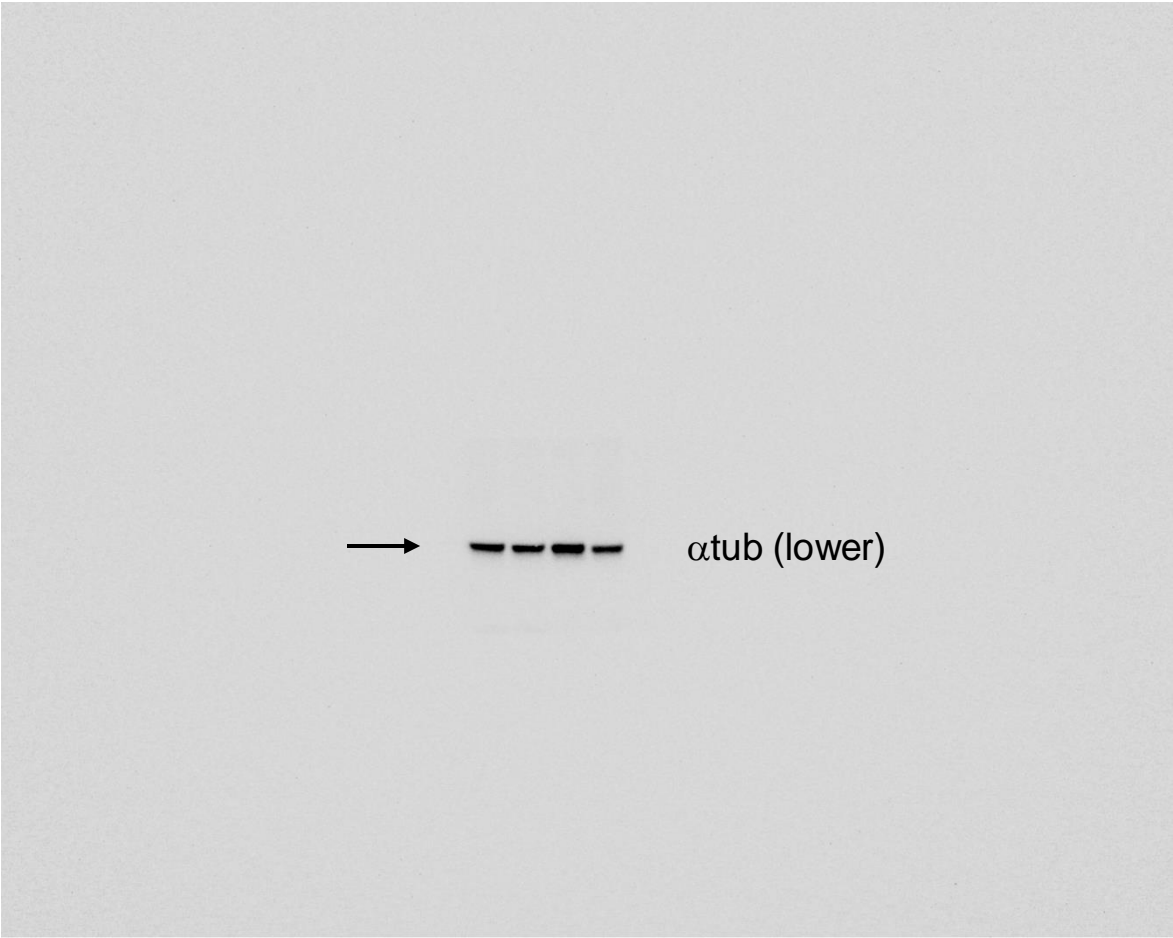

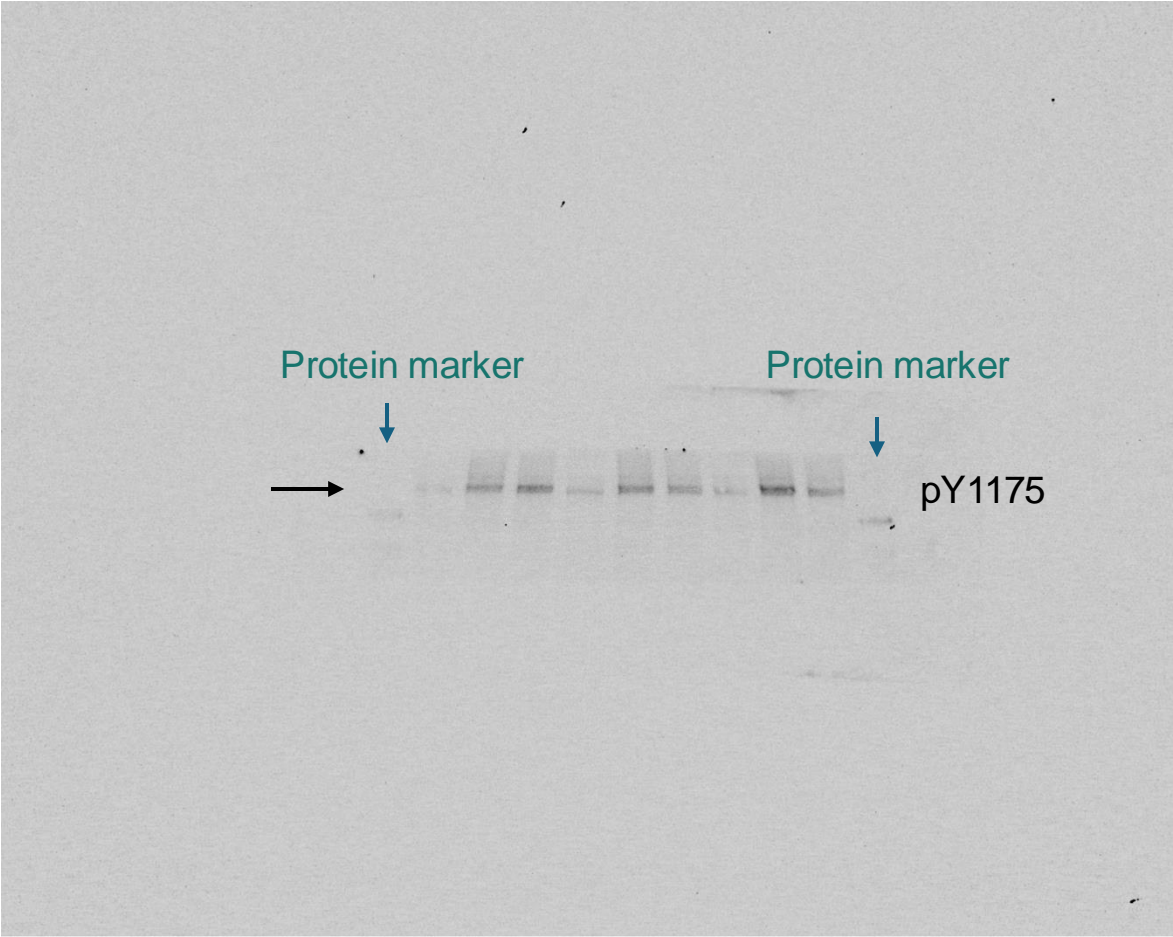

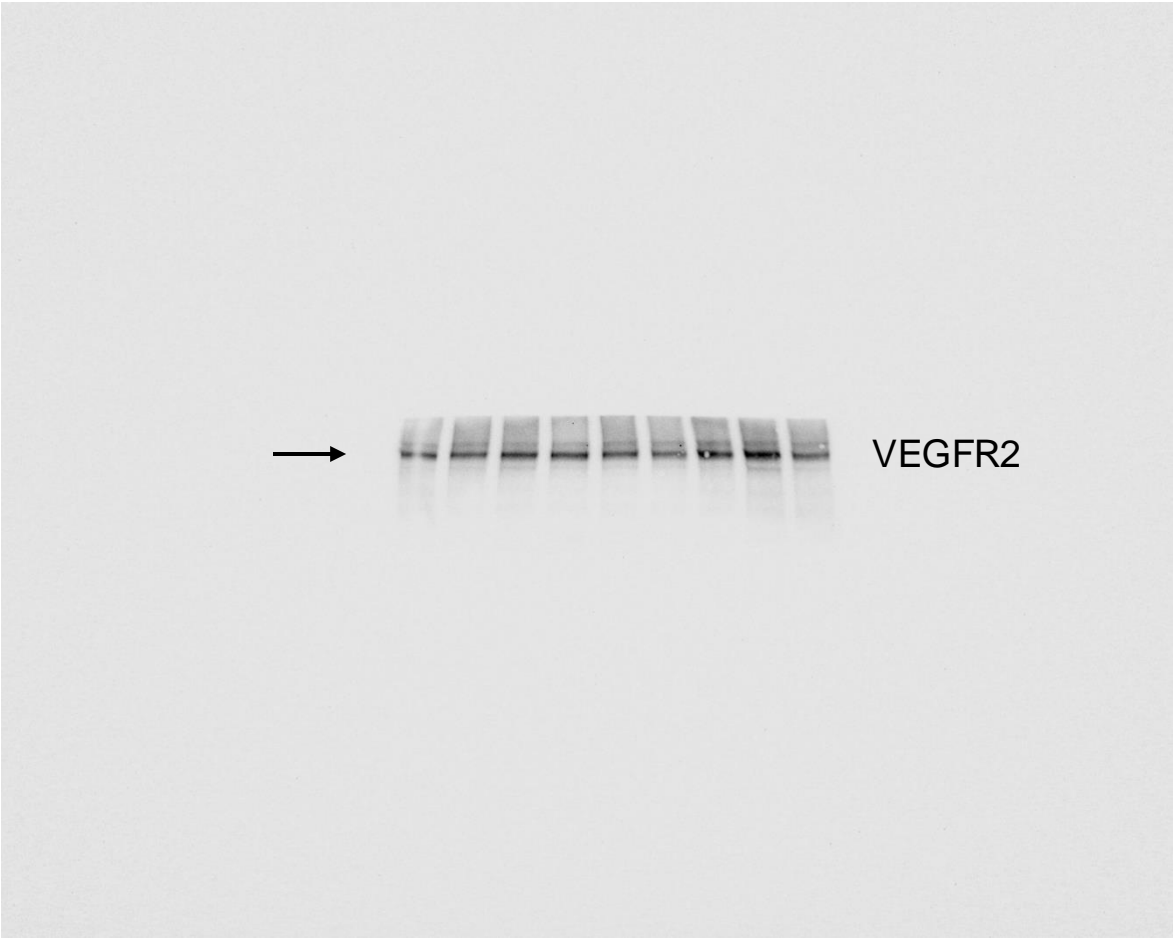

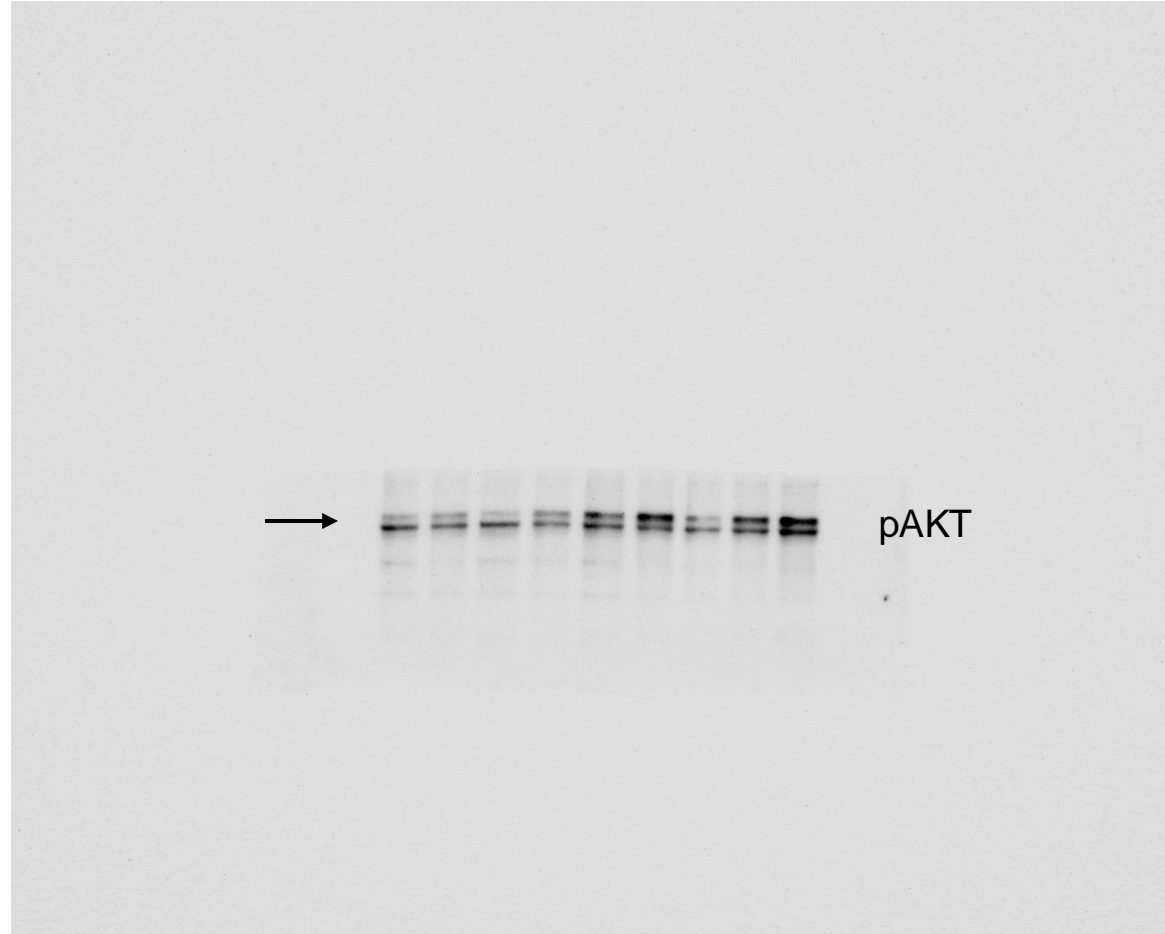

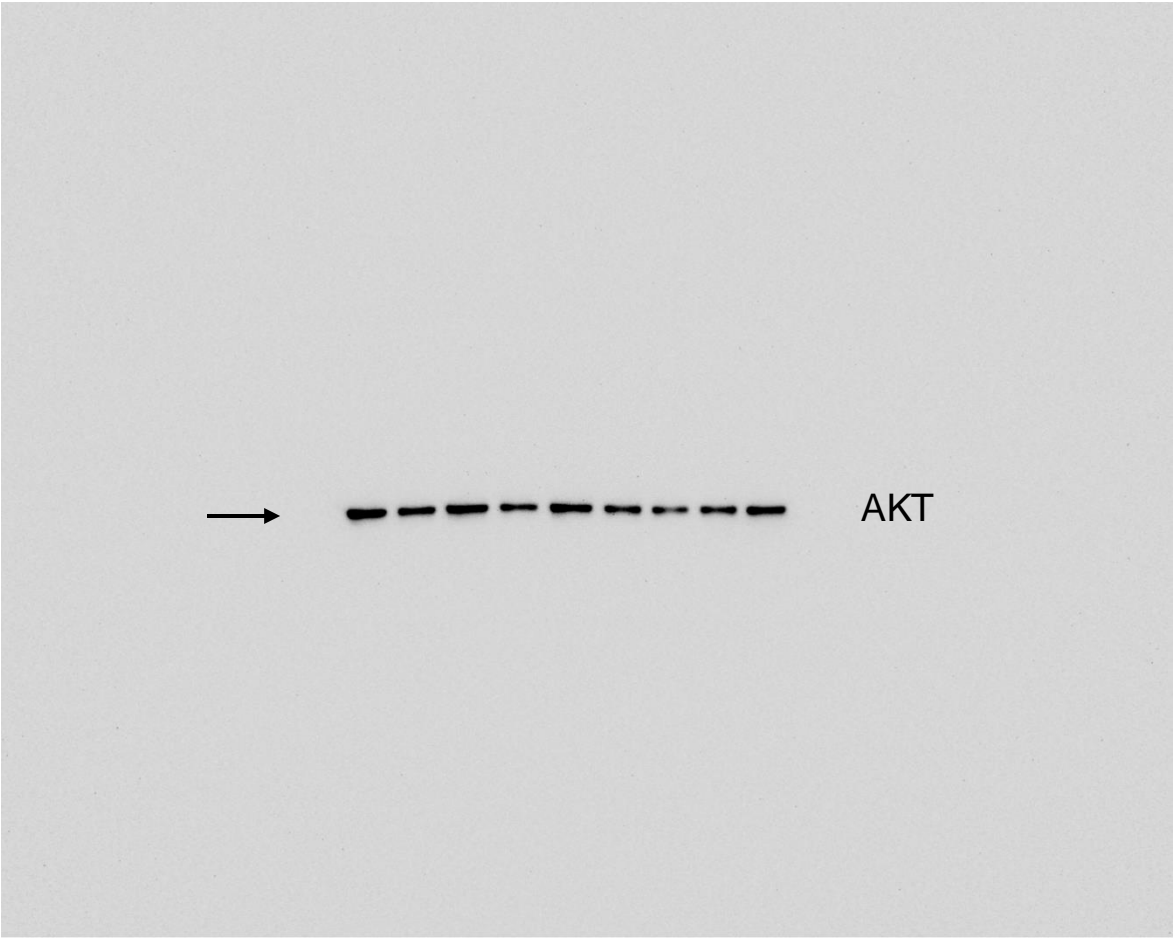

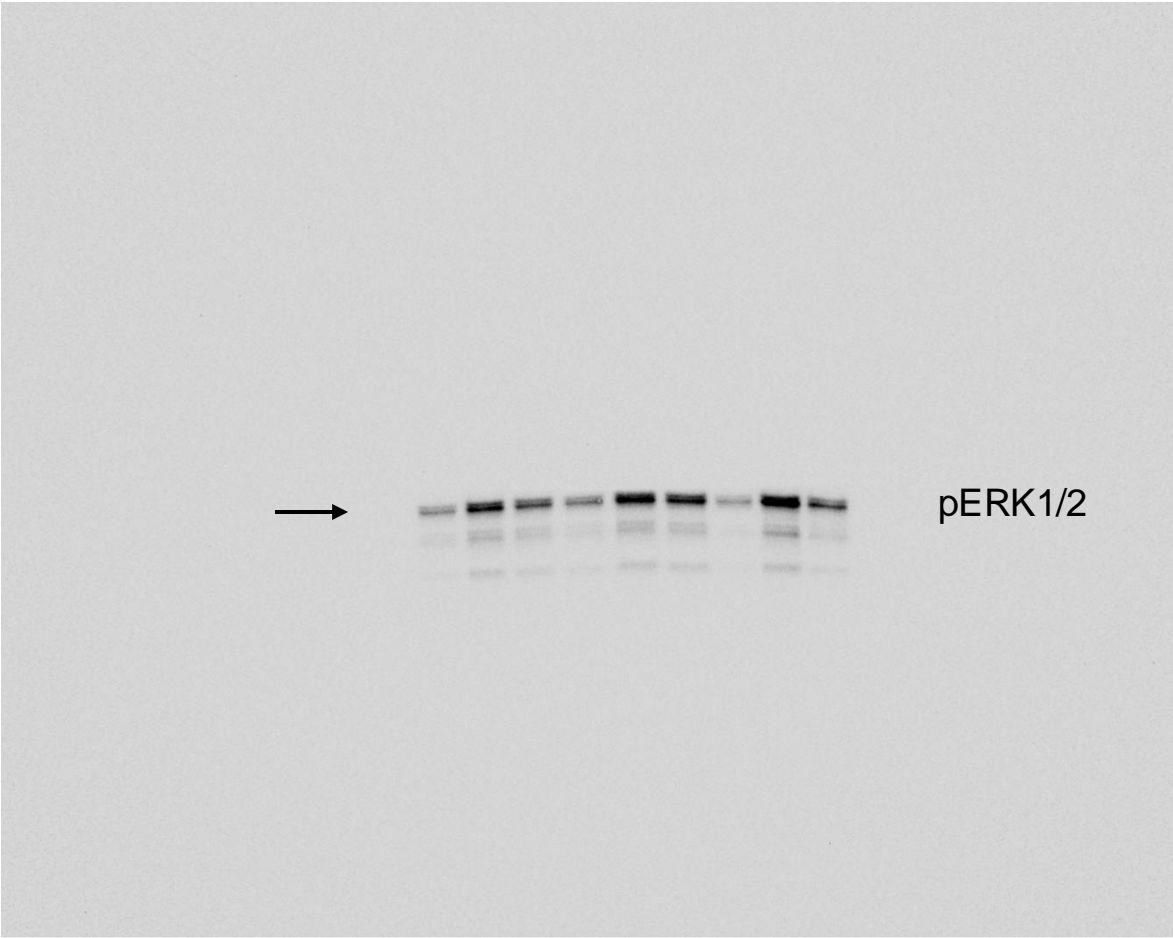

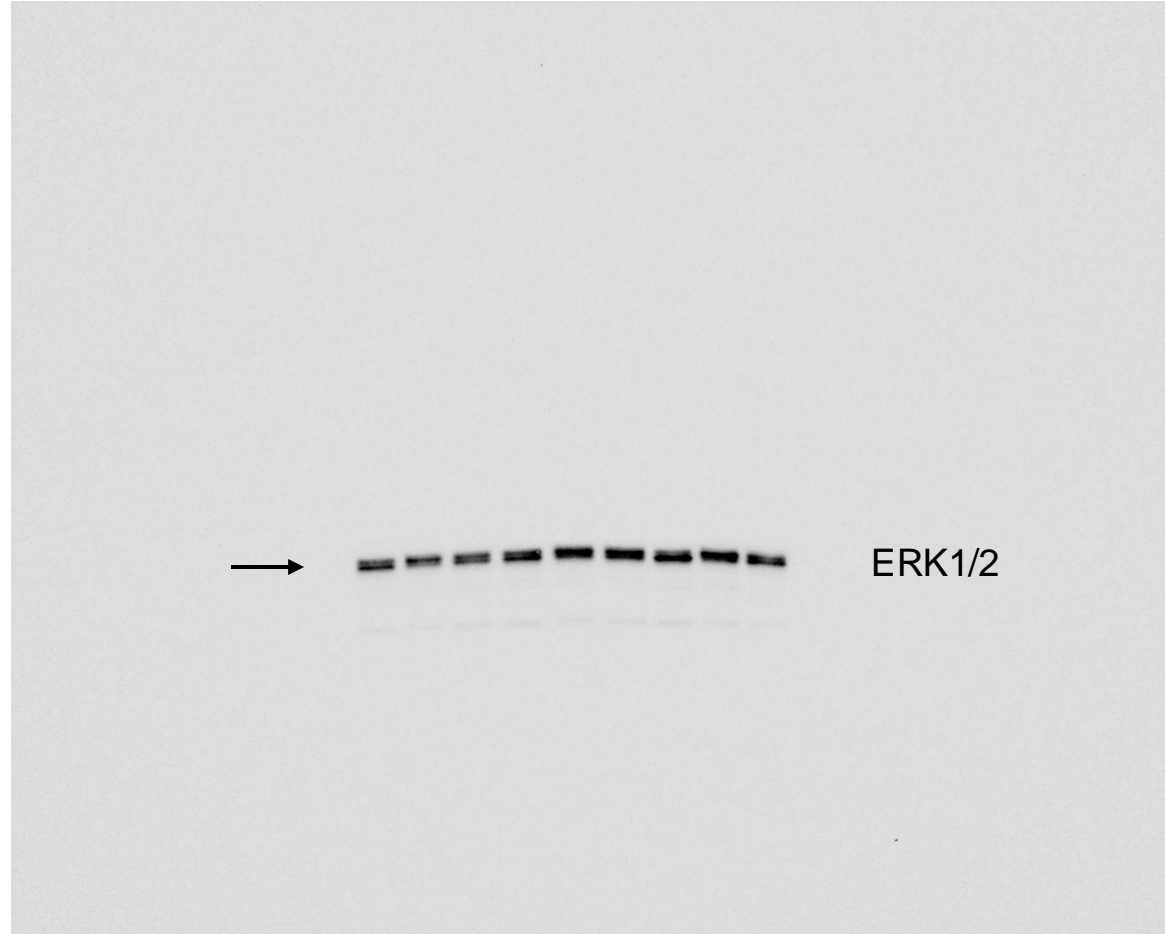

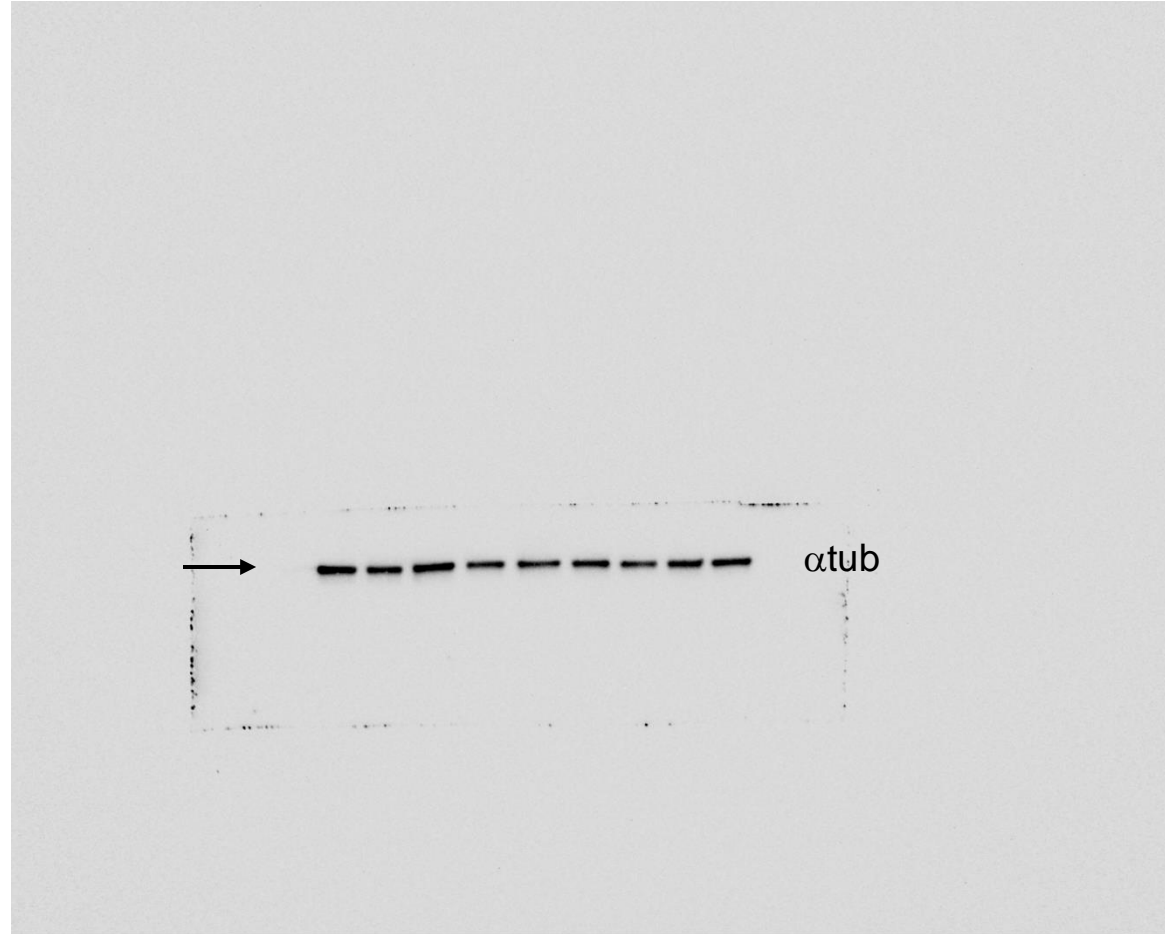

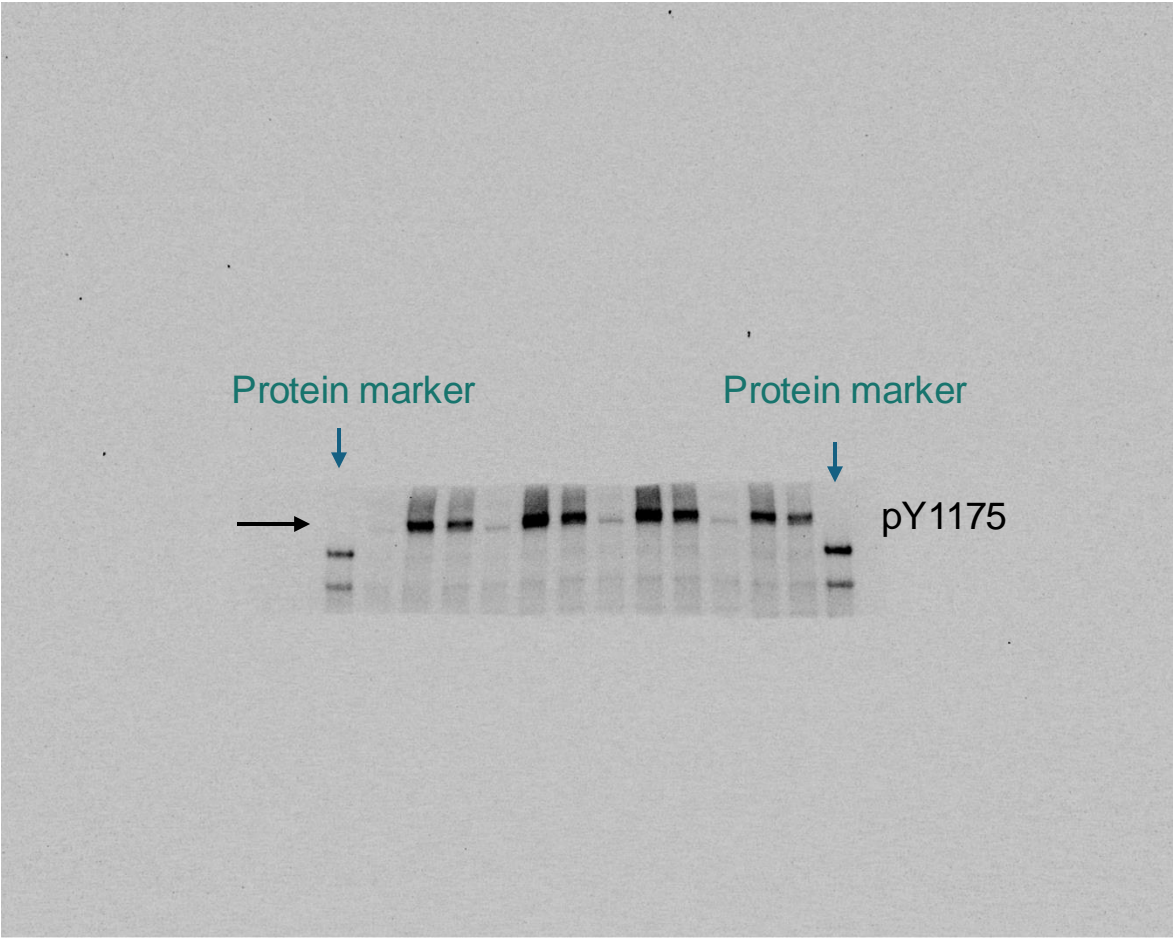

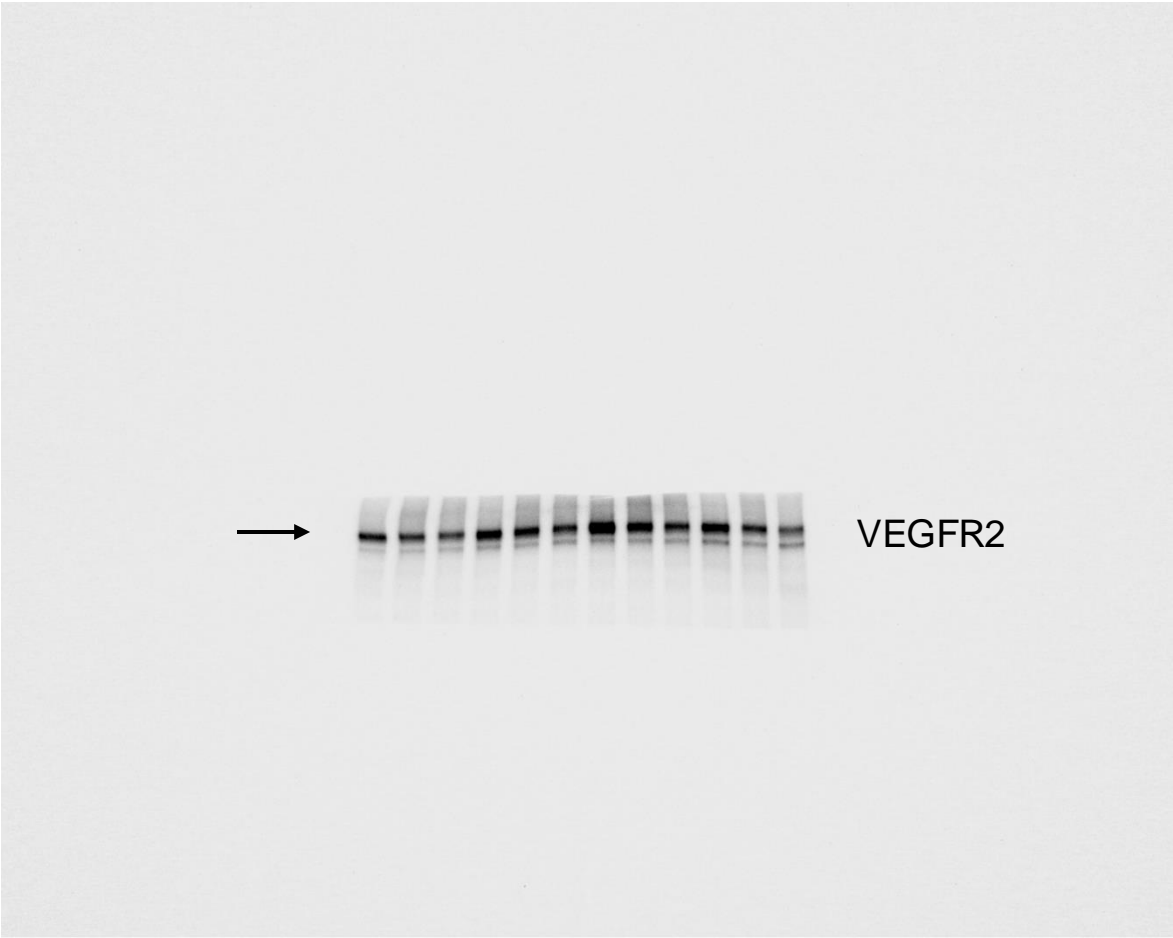

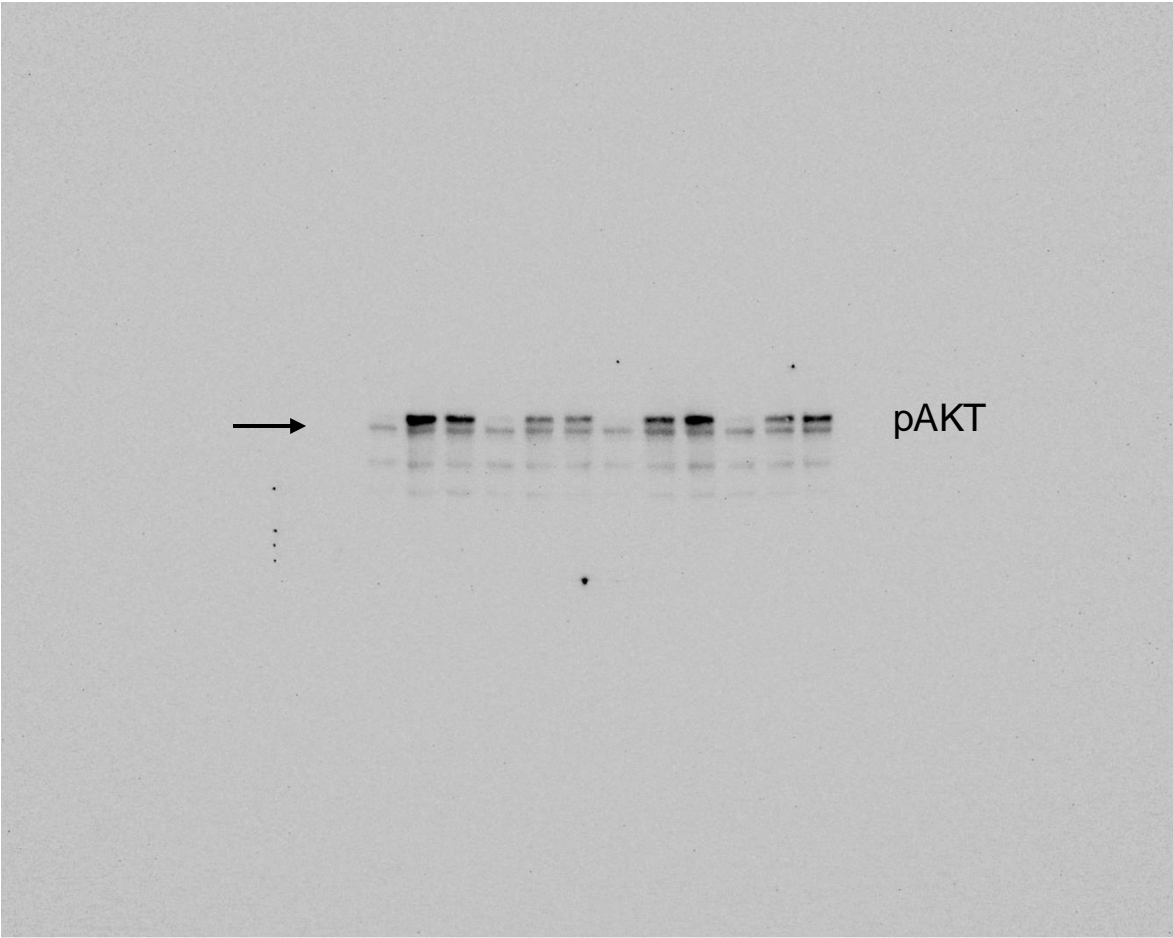

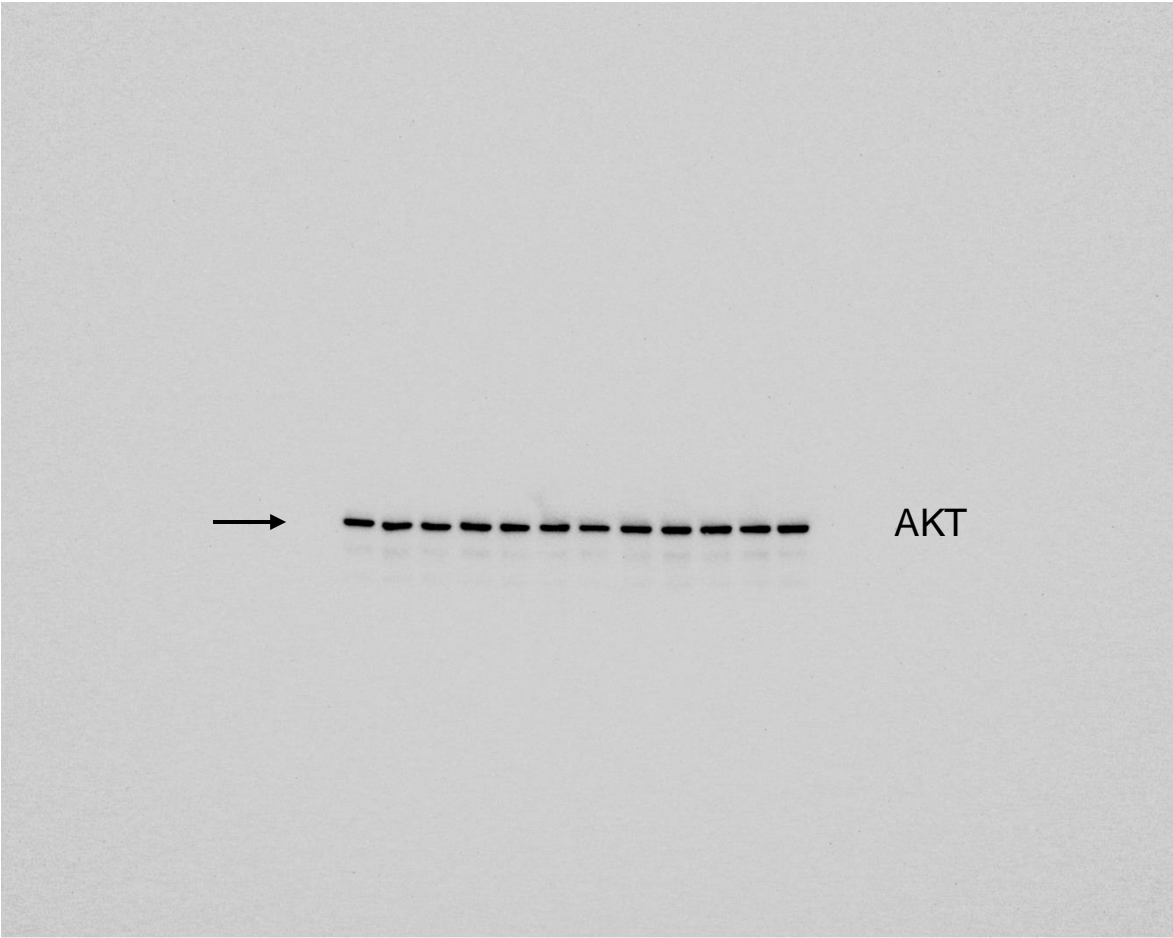

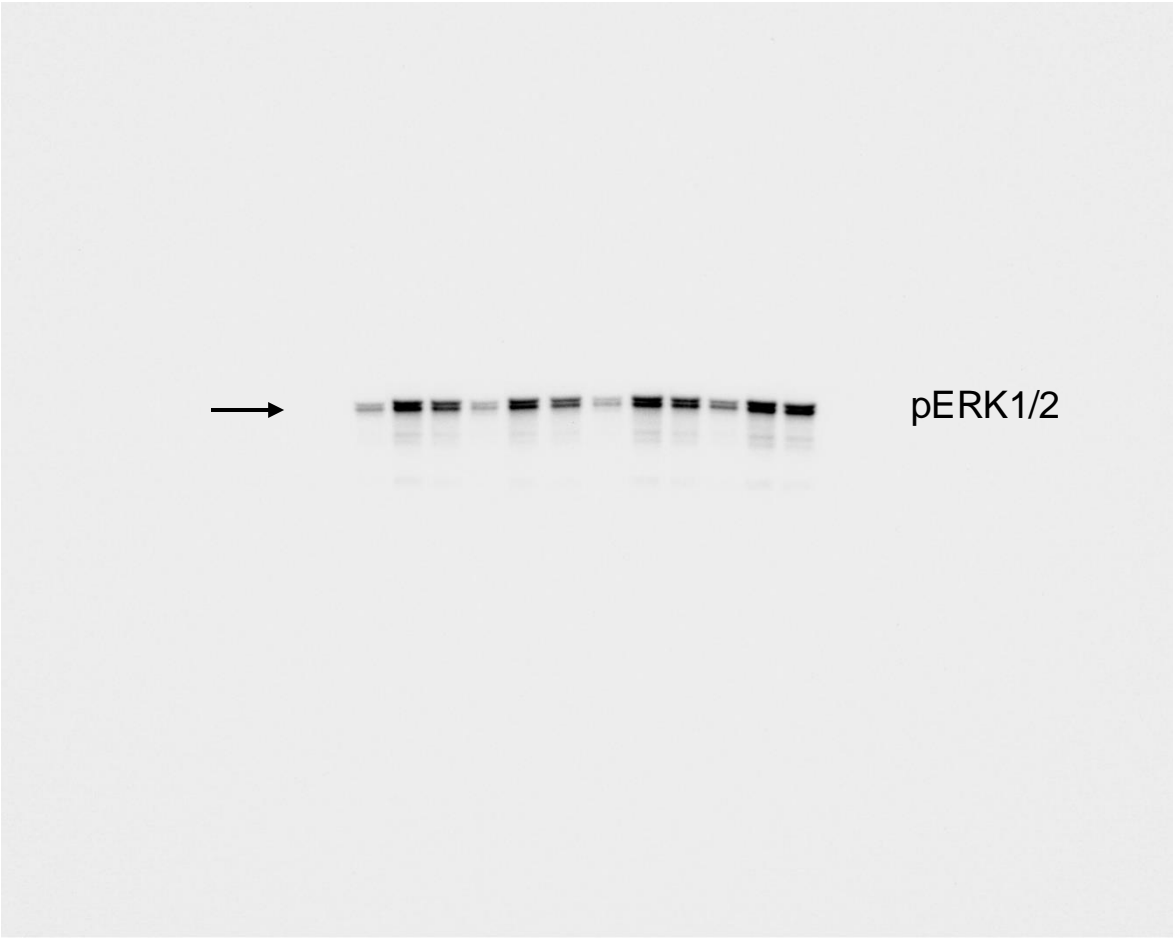

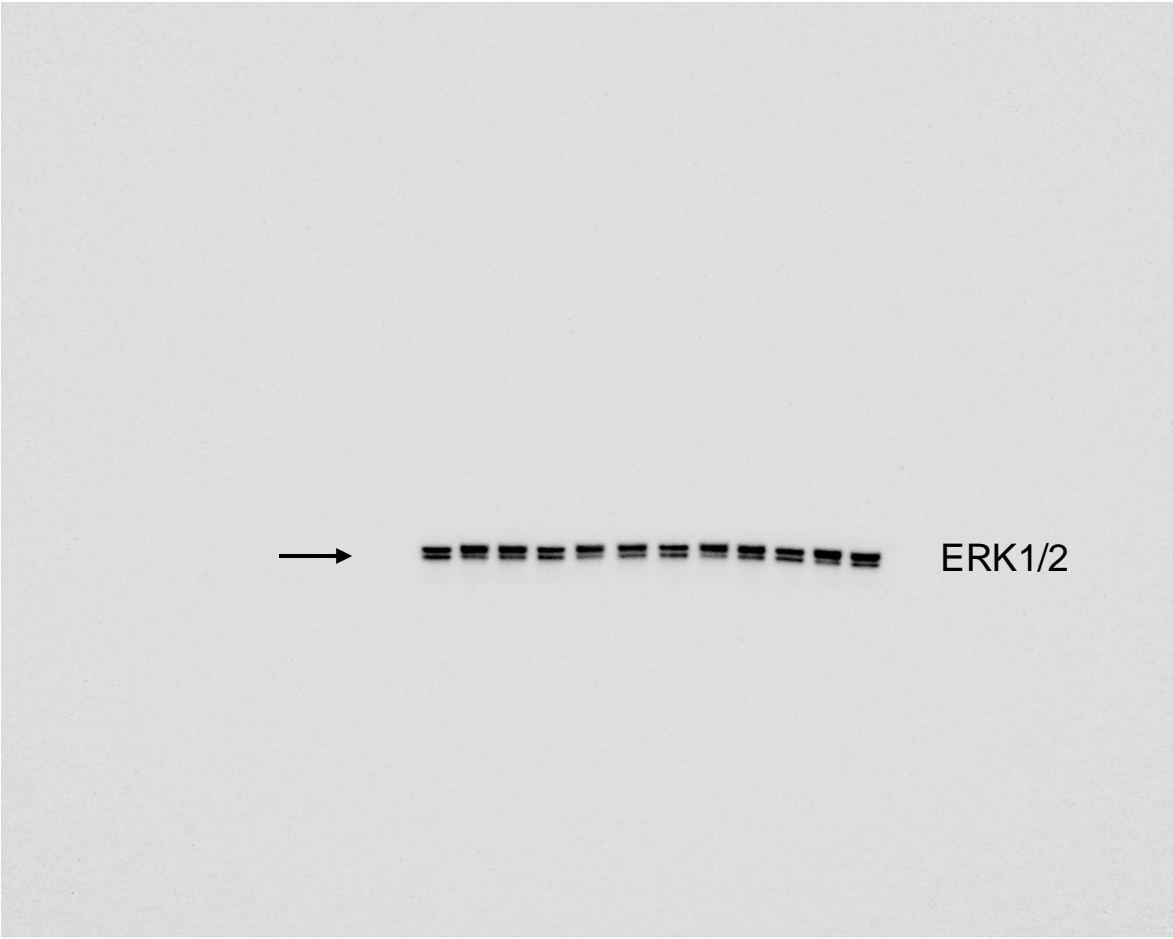

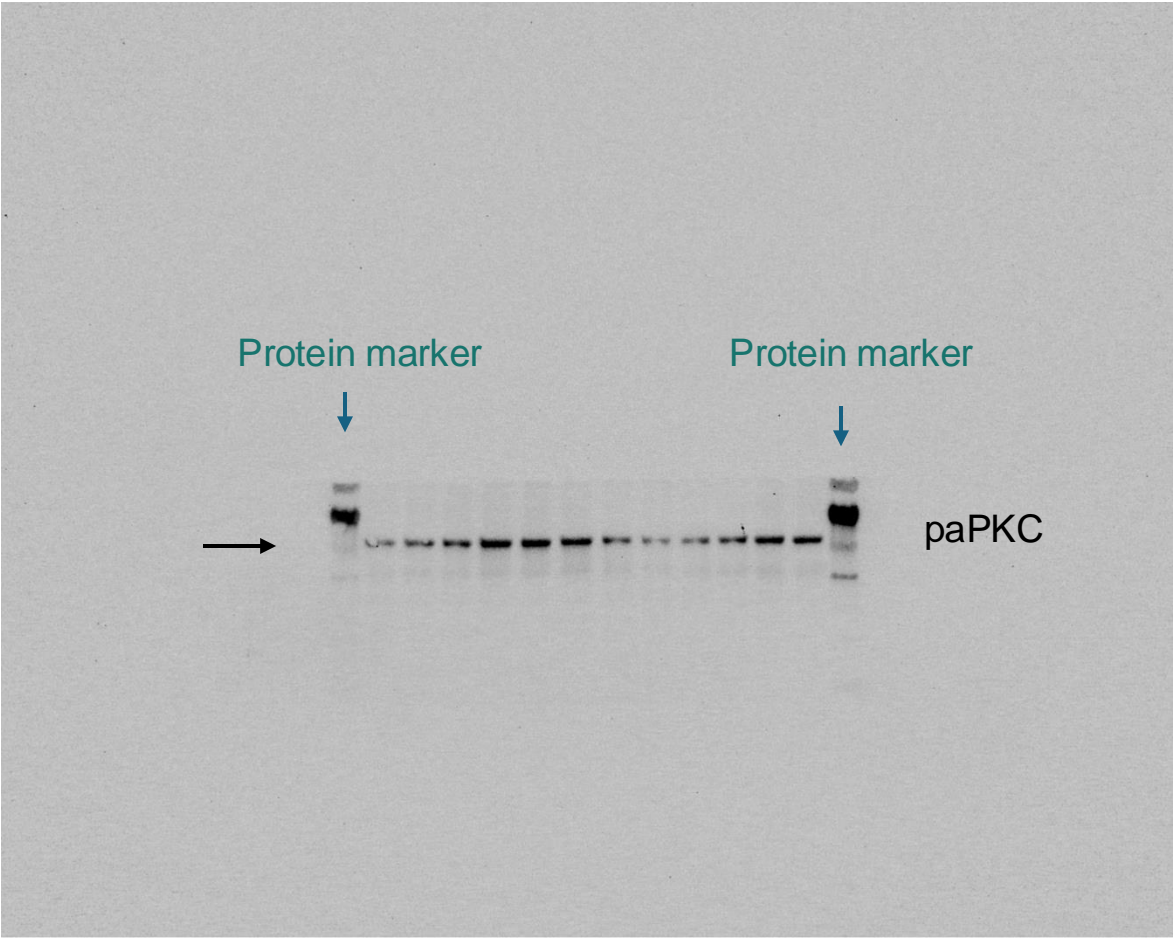

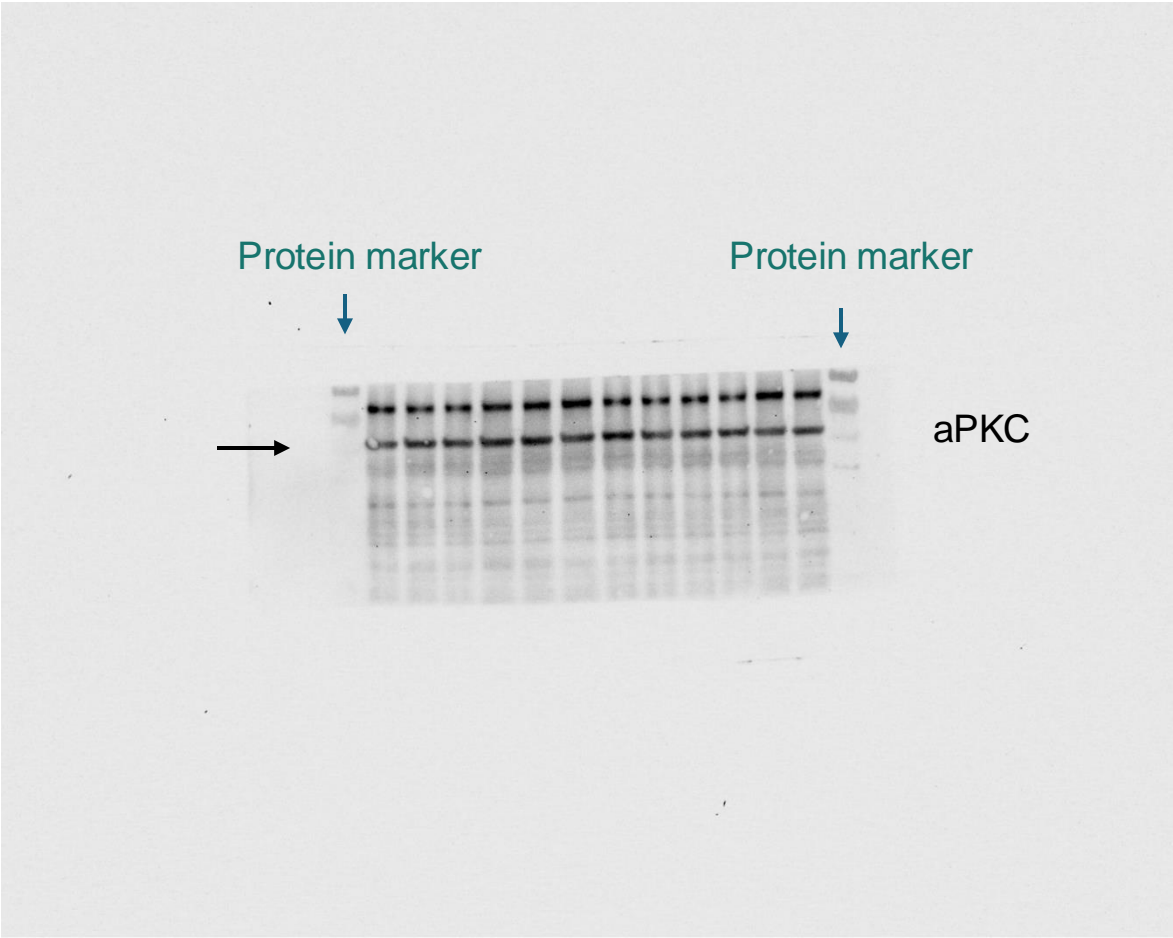

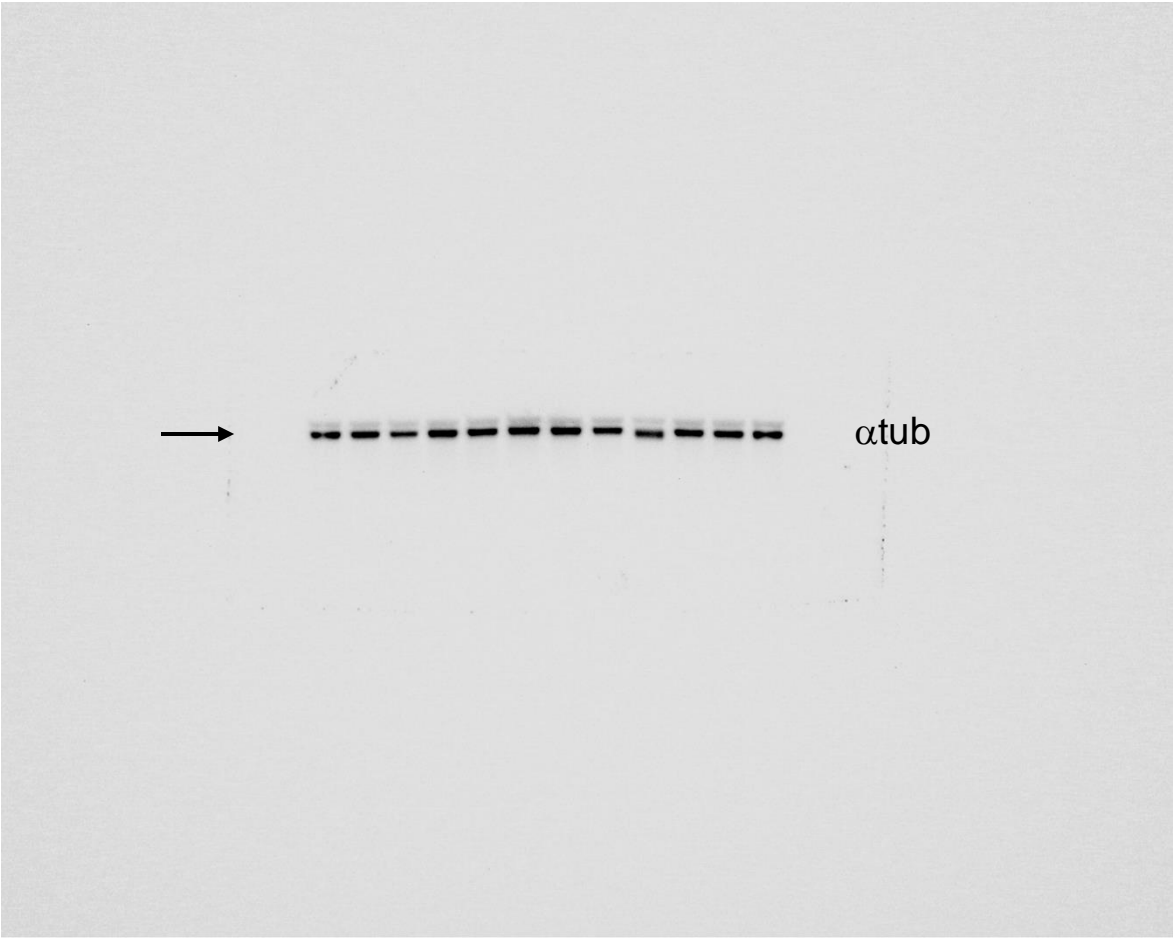

Supplement: Unedited blot and gel images [file jci-135-177601-s148.pdf]
